# Systematic mapping of bacteriophage gene essentiality with HIDEN-SEQ

**DOI:** 10.1038/s41564-026-02455-8

**Published:** 2026-09-25

**Authors:** Dorentina Humolli, Damien Piel, Jessica Ransome, Kathrin Bausch, Sarah Tschudin-Sutter, Monica Ortelli, Christoph Dehio, Jan-Willem Veening, Alexander Harms

**Affiliations:** 1 Department of Health Sciences and Technology, Institute of Food, Nutrition, and Health, ETH Zürich, Zurich, Switzerland; 2 Department of Urology, University Hospital Basel, Basel, Switzerland; 3 Division of Infectious Diseases, Department of Clinical Research, University Hospital Basel, Basel, Switzerland; 4 Biozentrum, University of Basel, Basel, Switzerland; 5 Department of Fundamental Microbiology, Faculty of Biology and Medicine, University of Lausanne, Lausanne, Switzerland

**Keywords:** Bacteriophages, Functional genomics, Phage biology

## Abstract

The arms race of bacteriophages and their bacterial hosts has inspired major breakthroughs in biotechnology and shaped phages as fierce predators with great clinical potential to fight multidrug-resistant bacterial pathogens. However, the large amount of genes of unknown function in phage genomes remains a major obstacle for the molecular understanding of phage–host interactions. Here we present HIDEN-SEQ (hidden Acr-enabled transposon-insertion sequencing), a transposon-insertion sequencing method for phages that systematically links viral genes to selectable phenotypes. Using model phage T4, we show that HIDEN-SEQ readily reproduces the gene essentiality map established over decades of research. Our method is easily portable across diverse non-model phages and reveals conditionally essential genes in multiple bacterial hosts and growth conditions, including previously unknown antidefence factors that we matched to specific antiviral defences. We anticipate that HIDEN-SEQ will be leveraged to reveal functions of viral genes with direct relevance for microbial ecology, biotechnology and phage therapy.

## Abstract (web summary)

HIDEN-SEQ (hidden Acr-enabled transposon-insertion sequencing) is a transposon-insertion sequencing method for phages that systematically links their genes to selectable phenotypes.

## Main

Bacteriophages, the viruses infecting bacteria, are the most abundant and genetically diverse biological entities on Earth^1,2^. Over billions of years, these viral predators and their hosts have co-evolved in a dynamic arms race that is constantly shaping microbial communities, balancing ecosystems and driving biogeochemical cycles around the globe^3–5^. Research on phage–host interactions using a few model phages has been crucial for our understanding of the basic molecular biology of life, for example, by unravelling the nature of the genetic code, and has provided the tools for critical technological breakthroughs such as molecular cloning^6^. Furthermore, the current crisis of antimicrobial therapy due to increasing rates of multidrug resistance is driving a renaissance of clinical applications of phages to combat bacterial infections (‘phage therapy’)^7^. However, a major limiting factor for phage-inspired biotechnology and rational applications of phage therapy remains the vast genetic ‘dark matter’ of enigmatic genes with no known biological function that dominate phage genomes^2,8^. We have therefore so far just scratched the surface of the true potential of these viruses to understand ecology, drive innovation in biotechnology and fight antibiotic-resistant infections in clinics.

Most tailed phages have genomes of 50–200 kb in size, of which one part is composed of operons of core genes encoding functions involved in phage replication, gene expression and virion assembly^2,9^. The other genes, largely coding for hypothetical proteins, are often dispensable under standard laboratory conditions and their proportion increases with genome size^9–11^. Based on a few previous studies, these accessory genes seem to provide functions that enhance the ability of the phage to survive and adapt to specific hosts or environmental conditions^11,12^. Well-known examples are T4 genes encoding inhibitors of specific antiviral defence systems such as *rIIA/rIIB* for RexAB or *dmd* for RnlAB and various anti-CRISPR proteins encoded in different phages^13,14^. Accessory genes of bacteriophages thus seem to largely form their arsenal of tools and tricks to prevail in the virus–host arms race. To systematically uncover the biological functions of these genes, scalable genome-wide approaches linking viral genes to specific phenotypes are required. Existing methods have offered valuable insights but remain laborious and not broadly applicable^15–20^. In bacteria, this challenge has been overcome by transposon-insertion sequencing (TnSeq), a technique that quantifies the fitness of all viable transposon insertions in a genome by comparative deep sequencing before and after growth under a given condition^21^. While TnSeq has revolutionized bacterial microbiology^22^, no analogous approach has so far been available for phages. Although transposition into phage genomes had occasionally been reported^23–25^, its scale and application have been limited by the lack of broadly applicable genetic selection markers.

Here, we present HIDEN-SEQ, a functional and easy-to-use TnSeq system for phages that combines highly efficient transposition into viral genomes with CRISPR-based selection for phages that have acquired a transposon. Using HIDEN-SEQ, we have built high-resolution gene essentiality maps for three different phages and demonstrate its utility for the identification of conditionally essential genes, including diverse new bona fide antidefence factors and genes that are crucial under specific growth conditions.

## HIDEN-SEQ enables TnSeq in phages

**Fig. 1:**
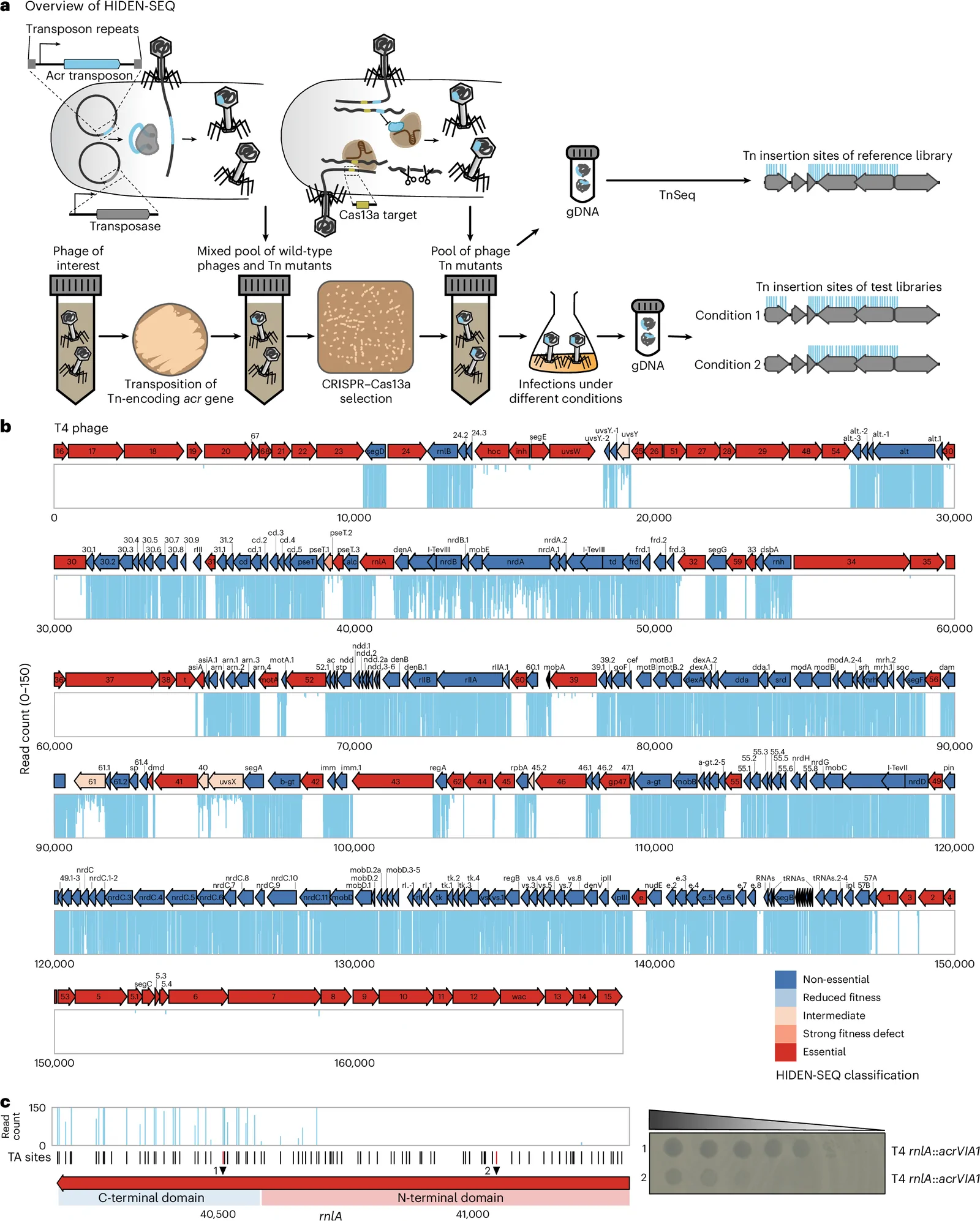
Combining transposition and CRISPR–Cas13a-based selection as HIDEN-SEQ for phage T4. **a**, Transposition introduces an anti-CRISPR gene (*acr*) randomly into TA dinucleotide sites within the phage genome. The *acr* expression inhibits CRISPR–Cas13a immunity and enables the selection of a library of phage transposon mutants. Deep sequencing of insertion sites is performed to quantify the abundance of transposon mutants before (as reference) and after exposing the library to different conditions. Tn, transposon. **b**, Phage T4 HIDEN-SEQ gene essentiality map with position 1 set to the start of the small terminase subunit gene. Arrows present the coding sequences with the orientation indicating the direction of transcription. Only the full-length gene products are shown. Arrow colours correspond to HIDEN-SEQ classification categories derived from transposon insertion profiles: non-essential (dark blue), reduced fitness (light blue), intermediate (light orange), strong fitness defect (orange) and essential (red). Vertical bars represent the number of transposon insertions at TA dinucleotide sites, with heights indicating total read counts (shown up to a maximum of 150). Genes containing five or fewer TA sites are shown in black and were not classified (Supplementary Data 4 (tabs S3 and S4)). **c**, Left: distribution of transposon insertions across the T4 *rnlA* gene with C-terminal and N-terminal domains indicated. Two TA sites disrupted in mutants used for validation are marked as site 1 and site 2. Right: tenfold serial dilution plaque assays of the T4 engineered mutants carrying insertions at sites 1 and 2 on the *E. coli* K-12 ΔRM strain, confirming the domain essentiality pattern observed in the HIDEN-SEQ data.

**Extended Data Fig. 1:**
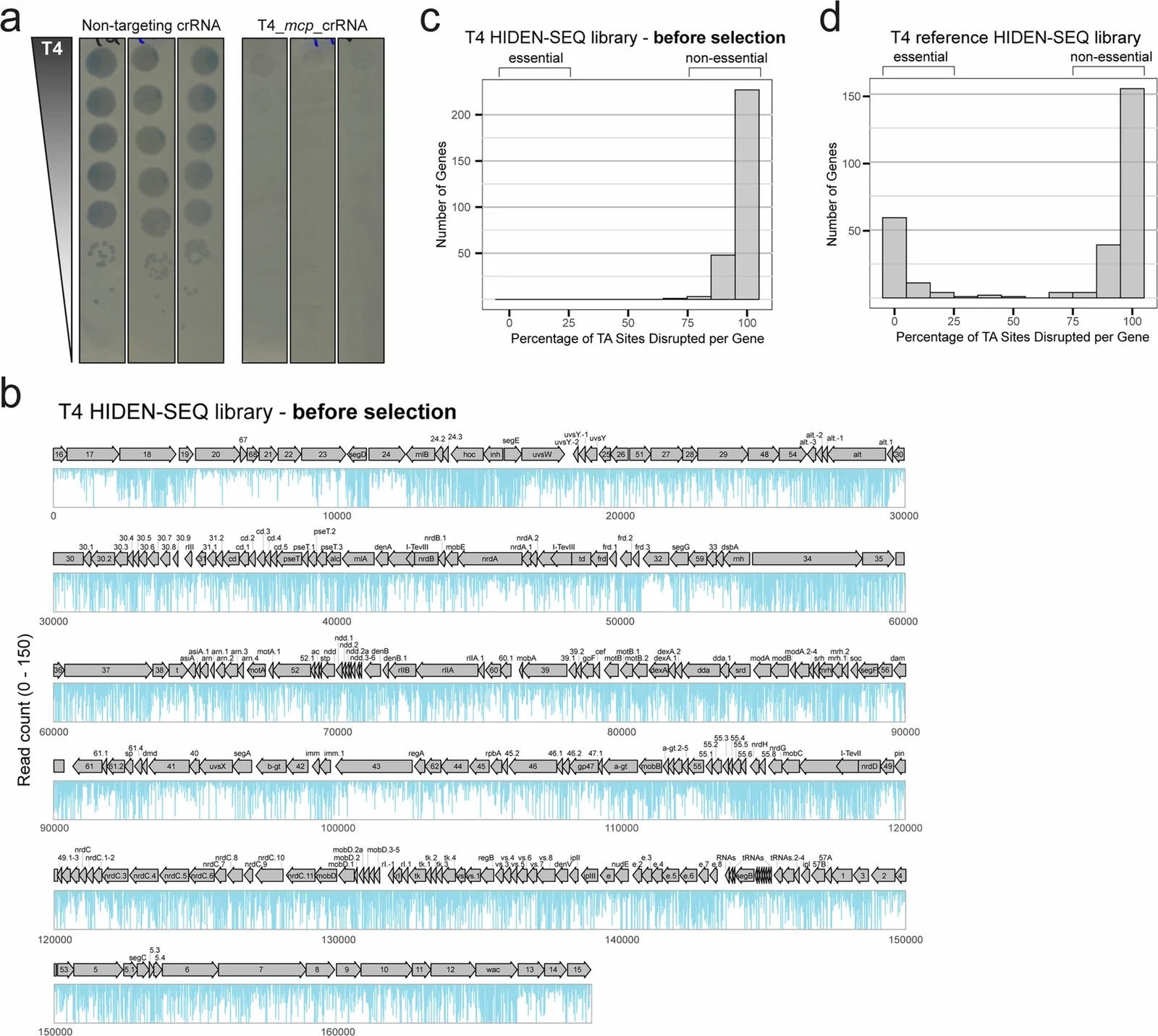
Genome-wide essentiality mapping of T4 phage. **a**, Ten-fold serial dilution plaque assays of T4 phage plated on *E. coli* K-12 ΔRM expressing *cas13a* with either a non-targeting crRNA control or a crRNA targeting the transcripts of the major capsid protein. **b**, Transposon insertion profiles of the T4 input HIDEN-SEQ library collected directly after the transposition step, that is, before strong selection against non-viable insertion mutants by viral replication. The start of the genome map has been set to the start of the small terminase subunit gene. Arrows present the coding sequences with the orientation indicating the direction of transcription. Only the full-length gene products are shown. Vertical bars represent the number of transposon insertions at TA dinucleotide sites with heights indicating total read counts (shown up to a maximum of 150). In total, 97.2% of TA sites have been hit. Read counts are not directly comparable across different phage input libraries because the fraction of informative transposon-genome junction reads varies between samples (Methods). **c**, **d**, Histograms illustrating the percentage of disrupted TA sites per gene for the T4 input HIDEN-SEQ library dataset (**c**) or the T4 reference HIDEN-SEQ library (**d**). The comparison shows that non-essential and essential genes are easily distinguishable in a highly saturated library (Supplementary Data 4 (tab S2 for **b** and **c**; tabs S3 and S4 for **d**)).

We began our study by setting the basics of TnSeq for bacteriophages using the well-studied model phage T4 infecting laboratory organism *Escherichia coli* K-12. For transposition, we built a new system based on mariner transposon Himar1 that is commonly used for TnSeq^26^ and inserts randomly at TA dinucleotide sites (Methods). In brief, the Himar1 transposon was engineered to encode the anti-CRISPR gene *acrVIA1*^27^ under the control of a strong constitutive promoter. For selection, we expressed the LseCas13a nuclease (type VI CRISPR effector) in the host with a potent CRISPR RNA (crRNA) targeting the transcripts of an essential T4 gene coding for the major capsid protein (Extended Data Fig. 1a). Only phage clones that have acquired the transposon, and thus express AcrVIA1, can evade Cas13a targeting and replicate^28^. CRISPR–Cas13a is well suited for selection of transposition across various phages because it recognizes and cleaves RNA^29^, not DNA, which inherently bypasses viral genome protection mechanisms of tailed phages that usually act directly at their dsDNA chromosome^28,30^. Importantly, target recognition of Cas13a also triggers non-specific degradation of phage and host mRNA, which aborts the viral infection^31^. We call this setup hidden Acr-enabled transposon-insertion sequencing (HIDEN-SEQ) because host-expressed CRISPR–Cas13a and the transposon-encoded Acr protein of the phage play ‘hide and seek’ in infected cells (Fig. 1a).

To assess whether transposition occurs efficiently in the phage genome and if these mutants can be selectively enriched, we plated T4, which had been targeted with the anti-CRISPR transposon or a non-protective control on the selection host (Supplementary Fig. 1a). As expected, CRISPR–Cas13a selection completely abolished T4 growth, but many viral clones were obtained after transposition with the anti-CRISPR transposon. Whole-genome sequencing of 13 individual clones confirmed that all carried unique transposon insertions (Supplementary Fig. 1b).

**Extended Data Fig. 2:**
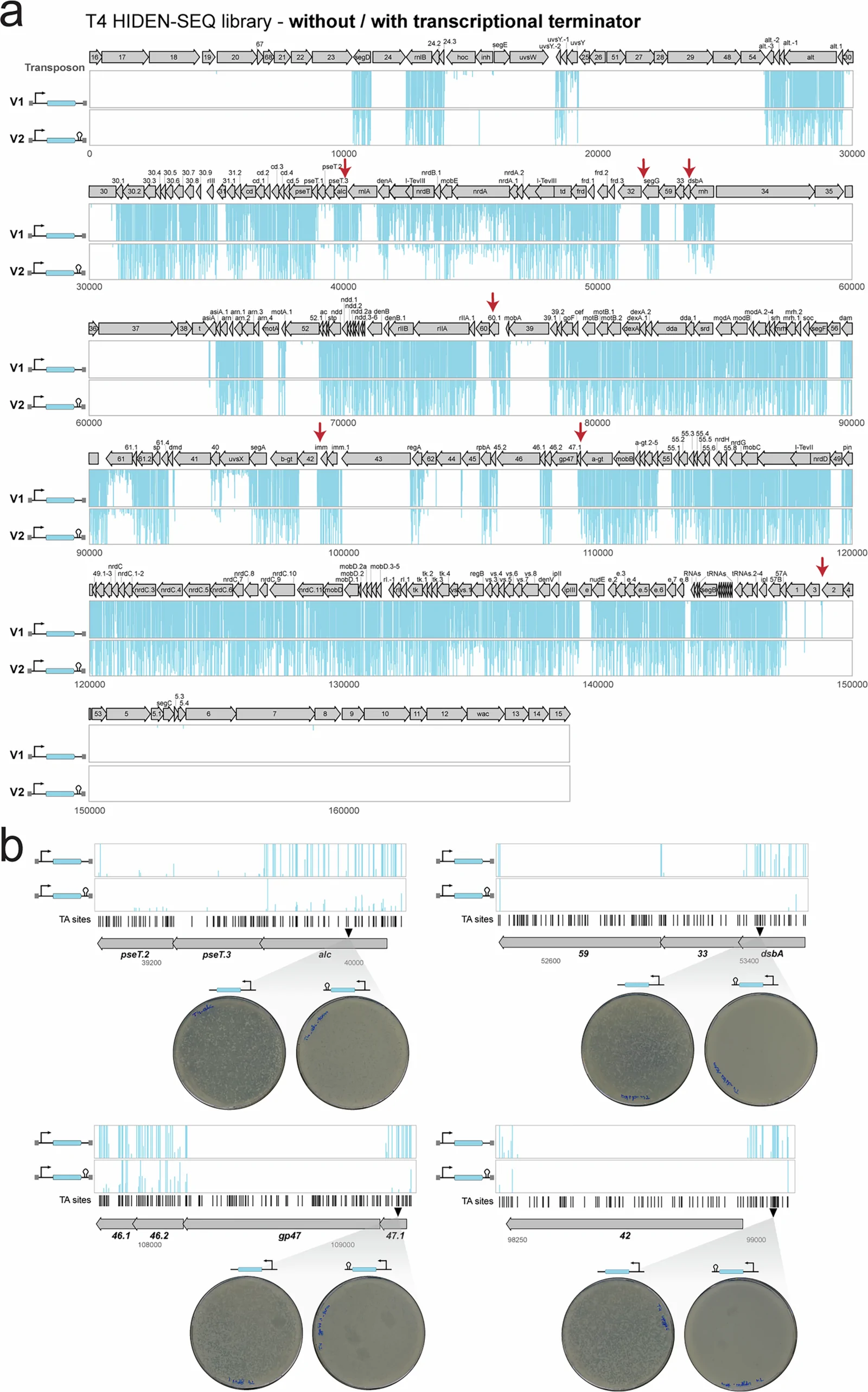
Impact of a transcriptional terminator on transposon insertion profiles in T4 HIDEN-SEQ libraries. **a**, Comparison of transposon insertion profiles in T4 HIDEN-SEQ libraries generated with the transposon lacking (V1, top track) or containing (V2, bottom track) a transcriptional terminator. The start of the genome map has been set to the start of the small terminase subunit gene. Arrows present the coding sequences with the orientation indicating the direction of transcription. Only the full-length gene products are shown. Vertical bars represent the number of transposon insertions at TA dinucleotide sites with heights indicating total read counts (shown up to a maximum of 150). Red arrows highlight some loci where insertion depletion upstream of essential genes is observed specifically in the library carrying the transcriptional terminator (Supplementary Data 4 (tabs S3 and S5)). **b**, Zoomed-in views of loci on phage T4 (*alc*, *dsbA*, *47.1*, and upstream of *42*) where targeted insertions were performed. Transposon insertion profiles are shown for libraries generated without (top track) or with (bottom track) the transcriptional terminator. Vertical bars represent the number of transposon insertions at TA sites (shown with black tick marks), with heights indicating total read counts (shown up to a maximum of 150). The targeted insertion sites are marked by black arrows. For each locus, the corresponding whole-plate infection on the Cas13a selection strain are shown below, comparing recovery of engineered insertion mutants with and without the transcriptional terminator downstream of the selection marker.

Subsequently, we applied HIDEN-SEQ to build a genome-wide gene essentiality map of model phage T4. This phage has a 168-kb genome encoding nearly 300 genes, of which around 130 have remained uncharacterized despite decades of research^10^. The T4 input HIDEN-SEQ library, collected before CRISPR–Cas13a selection, showed abundant insertions distributed across the entire genome (97.2% saturation of TA sites; Extended Data Fig. 1b,c), confirming efficient transposition. Insertions in essential genes can be readily detected at this step because the library still contains mutants from the initial viral burst after transposition with minimal subsequent replication. After outgrowth, transposon insertions in essential genes were strongly depleted, showing that 190 genes in the T4 genome are not essential for infection of *E. coli* K-12 laboratory strain under standard laboratory conditions, which is well in agreement with literature^10^ (Fig. 1b and Extended Data Fig. 1d). Only very few genes (*inh, hoc, segE, uvsW, segC* and *wac*) appeared essential in our screen despite being reported as not essential in at least some previous studies that had used other methods^10,17^. One possible reason for these minor discrepancies are polar effects of transposition as an inherent caveat of TnSeq^21,32^. However, we deliberately used a transposon without transcriptional terminator to minimize the disruption of local gene expression, and experimental introduction of such a terminator indeed caused strong polar effects (Extended Data Fig. 2a,b and Supplementary Discussion A).

Importantly, the high resolution enabled by HIDEN-SEQ allowed us to extend the essentiality classification beyond binary calls to include weakly, moderately and strongly reduced fitness of gene disruptions (Methods). As an example, five genes in the T4 genome (*40*, *45.2*, *61*, *uvsX* and *uvsY*) showed a low but still detectable tolerance to transposon insertions, indicating that their disruption imposed a fitness defect but did not completely abolish phage viability (Fig. 1b). Furthermore, HIDEN-SEQ enables subgene resolution of fitness analyses in some cases (Methods), as illustrated by T4 RNA ligase A (*rnlA)* that tolerates insertions only in its C-terminal domain, which had previously been shown to target this enzyme towards tRNA breaks in the anticodon loop^33^ (Fig. 1c). Taken together, these results establish HIDEN-SEQ as an effective tool for mapping phage gene essentiality at a very high depth and resolution.

## HIDEN-SEQ identifies known and previously undescribed conditionally essential genes in phage T4

**Fig. 2:**
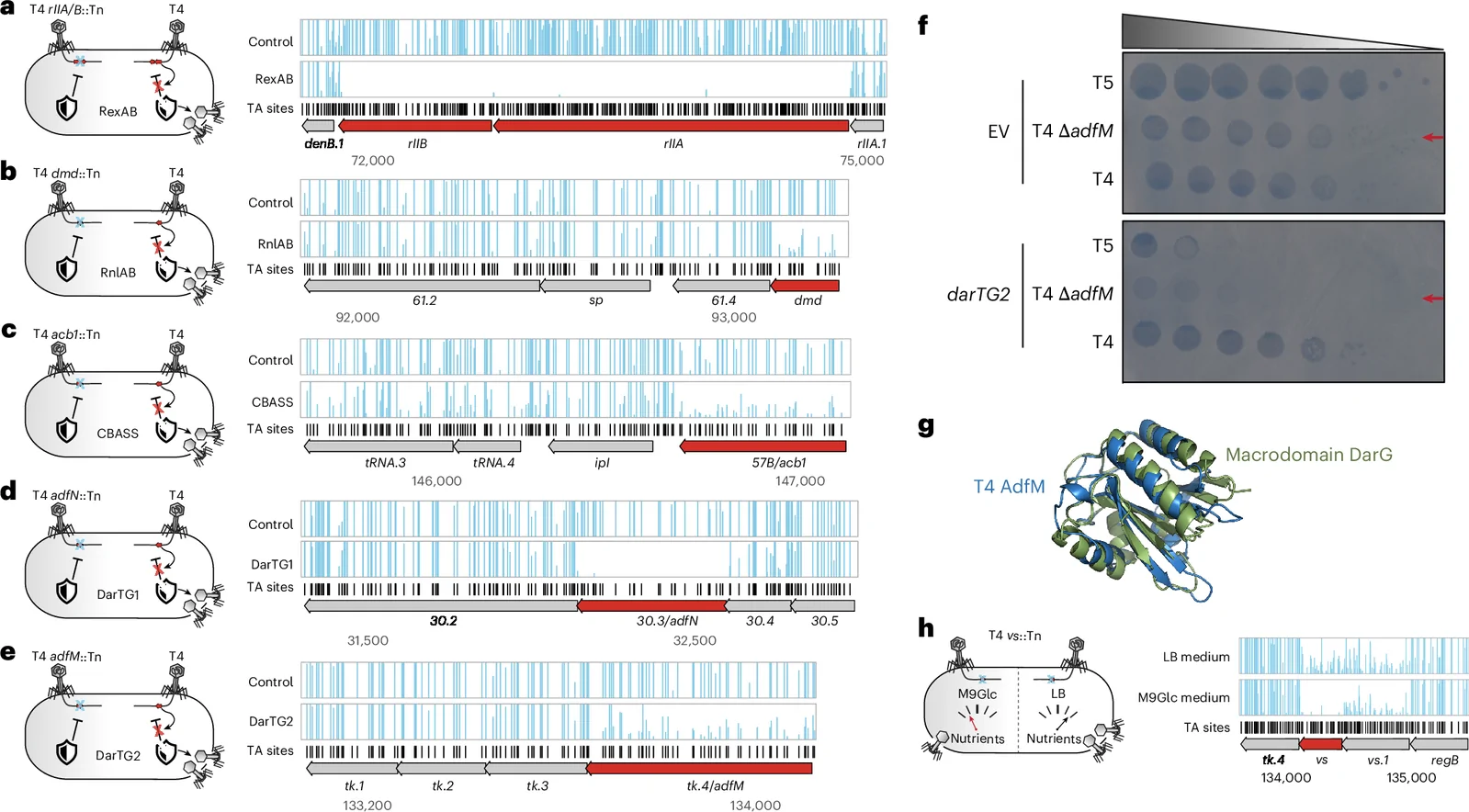
Identification of conditionally essential phage genes by HIDEN-SEQ. The T4 phage reference HIDEN-SEQ library was used to infect different variants of the *E. coli* K-12 BW25113 host strain to compare viral gene essentiality in the presence of different antiphage defence systems. **a**–**e**, For each panel, transposon insertion profiles obtained after infection in the presence of the defence system condition (bottom track) are compared with the respective control (top track). Vertical bars represent the number of transposon insertions at TA sites (shown with black tick marks) with heights indicating total read counts (shown up to a maximum of 150). Arrows indicate coding sequences with orientation showing direction of transcription. Genes highlighted in red were identified as conditionally essential in the presence of the indicated defence system and are shown with their surrounding genomic regions. Empty vector control compared with *E. coli* K-12 expressing *rexAB* (**a**). *E. coli* K-12 Δ*rnlA* compared with *E. coli* K-12 expressing *rnlAB* (**b**). *E. coli* K-12 carrying a plasmid encoding a catalytically inactive (control) or active CBASS operon (**c**). Empty vector control compared with *E. coli* K-12 expressing *darTG1* (**d**). Empty vector control compared with *E. coli* K-12 expressing *darTG2* (Supplementary Data 4 (tabs S7–S16)). (**e**). **f**, Tenfold serial dilution plaque assays on *E. coli* K-12 carrying an empty vector control or *darTG2* expression plasmid challenged with control phage T5 (sensitive to DarTG2), phage T4 engineered to remove AdfM (Δ*adfM*) or wild-type phage T4. Data are representative of three independent biological replicates. EV, empty vector. **g**, Structural alignment of AlphaFold 2 predicted structures of T4 AdfM with the macrodomain of *M. tuberculosis* DarG homologue (HHPred probability score 99.71%, e-value 2.6 × 10^−14^). **h**, Transposon insertion profiles obtained after infection of *E. coli* K-12 BW25113 with T4 reference HIDEN-SEQ library in M9Glc medium (bottom track) and LB medium (top track), highlighting in red the strongest depleted *vs* gene in the M9Glc condition. Vertical bars represent the number of transposon insertions at TA sites (shown with black tick marks), with heights indicating total read counts (shown up to a maximum of 150) (Supplementary Data 4 (tabs S17 and S18)).

**Extended Data Fig. 3:**
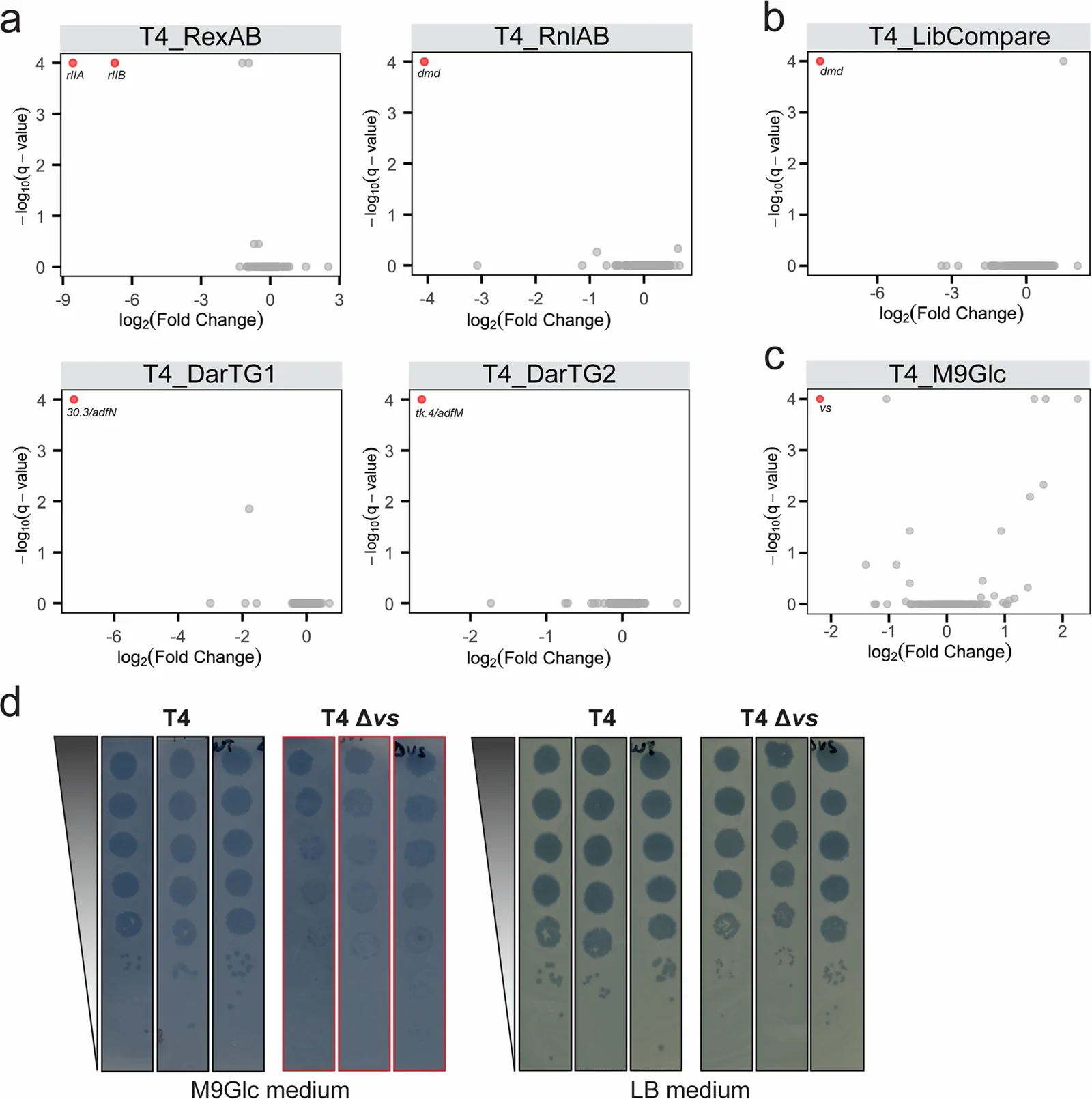
Context-dependent essentiality of T4 phage genes. **a**, Volcano plots comparing the depletion of gene disruptions in phage T4 after infection of *E. coli* K-12 BW25113 derivatives expressing *rexAB, rnlAB, darTG1* or *darTG2* relative to the corresponding control conditions. Each point represents a viral gene with the *x* axis showing the log_2_FC in normalized insertion counts and the *y* axis showing the –log_10_(*q* value) (Methods). Negative log_2_FC values indicate that mutants with insertions in that gene had reduced fitness in the presence of defence system relative to its control. No volcano plot is shown for CBASS system because toxicity of defence genes prevented robust statistical analysis despite the clear hit of *acb1* (Methods) (Supplementary Data 4 (tabs S24–S27)). **b**, Volcano plot comparing the depletion of gene disruptions in phage T4 between the reference HIDEN-SEQ library and the library generated on the *E. coli* K-12 *rnlA* knockout, that is, showing the effect of native *rnlAB* expression on *dmd* essentiality. Each point represents a viral gene; axes as in (**a**) (Supplementary Data 4 (tab S23)). **c**, Volcano plot comparing the depletion of gene disruptions in phage T4 after infection of *E. coli* K-12 BW25113 laboratory strain in rich LB medium and in M9Glc minimal medium. Each point represents a viral gene; axes as in (**a**). Negative log_2_FC values indicate that mutants with insertions in that gene had reduced fitness in the M9Glc medium condition compared to the LB medium (Supplementary Data 4 (tab S28)). **d**, Ten-fold serial dilution plaque assays of wildtype T4 and a *vs* knockout mutant (Δ*vs*) plated on *E. coli* K-12 BW25113 for growth on agar plates prepared with M9Glc or LB medium. The reduced growth of a viral *vs* mutant in the M9Glc is apparent.

To test whether HIDEN-SEQ can also identify conditional essential genes, we used our highly saturated T4 HIDEN-SEQ library to infect hosts expressing different bacterial defence systems. This allowed us to directly evaluate whether insertions in known antidefence genes would be selectively depleted when challenged by the targeted defence system. We first examined the *rIIA* and *rIIB* genes of T4, which counteract the RexAB defence system^13^. As expected, transposon insertions in *rIIA*/*rIIB* genes were significantly depleted only when the T4 library was grown in the presence of RexAB, while both genes were non-essential without it (Fig. 2a and Extended Data Fig. 3a). Subsequently, we focused on *dmd*, which encodes an inhibitor of the antiviral RnlAB toxin–antitoxin system^34^. Our initial experiments had identified the *dmd* gene of T4 phage as essential because the *E. coli* K-12 laboratory strain naturally expresses RnlAB^34^ (Fig. 1b). We thus generated another T4 transposon library in an *rnlA* knockout strain as host and used it to infect this mutant and the parental wild-type host. As expected, transposon insertions in *dmd* were strongly depleted in the host expressing RnlAB but not in the knockout control (Fig. 2b and Extended Data Fig. 3a). Importantly, when comparing the two independently generated T4 libraries, no other gene showed significant differences in the distribution of transposon insertions, highlighting both the specificity of bacterial immunity and the reproducibility of HIDEN-SEQ (Extended Data Fig. 3b).

In addition, T4 phage encodes an anti-CBASS protein (57B/Acb1) which degrades the cyclic nucleotides generated by this defence system as a second messenger in response to phage infection^35^. As expected, we observed a clear reduction in read counts for insertions in *57B/acb1* specifically when we challenged the transposon library with a host ectopically expressing CBASS, which is naturally absent from *E. coli* K-12^35^ (Fig. 2c). Finally, we used HIDEN-SEQ to probe the biology of an anti-DarTG gene of phage T4, *30.3/adfN*, that encodes an enzyme reverting the ADP-ribosylation of DNA, which is added by the DarT toxin to block viral replication^36^. As expected from previous work^36^, *30.3/adfN* was found to be specifically essential for growth on *E. coli* K-12 only in presence of the DarTG1 defence system (Fig. 2d and Extended Data Fig. 3a).

While *adfN* protects T4 against DarTG1, it does not act against DarTG2, probably because AdfN targets modified guanosine bases whereas DarTG2 modifies thymidines^36^. As T4 lacking *adfN* remains resistant to DarTG2^36^, we suspected that T4 may additionally encode an anti-DarTG2 factor. To test this, we used the T4 HIDEN-SEQ library to infect a host expressing DarTG2 and found a single significant hit, *tk.4*, which showed reduced read counts specifically in presence of this defence system (Fig. 2e and Extended Data Fig. 3a). Consistently, a knockout of *tk.4* sensitized phage T4 to DarTG2 (Fig. 2f). Interestingly, Tk.4 is homologous to the DarG antitoxin of *Mycobacterium tuberculosis* DarTG2^37^, and both proteins feature macrodomains that typically act as an ADP-ribosylglycohydrolase^38^ (Fig. 2g). We therefore conclude that *tk.4* encodes a previously unknown anti-DarTG2 factor of phage T4, which we name anti-DarT factor macrodomain (*adfM*). AdfM probably neutralizes DarT2 toxicity by enzymatic removal of ADP-ribose modifications from the DNA, as recently shown for another phage-encoded AdfM^39^.

To test whether conditionally essential genes can also be identified beyond the context of bacterial immunity, we compared the mutant fitness of the T4 HIDEN-SEQ library in rich Lysogeny Broth (LB) medium and defined M9 minimal medium (Methods). Under nutrient limitation, the strongest depletion was observed for the T4 *vs* gene encoding a modifier of valyl-tRNA synthetase (Fig. 2h and Extended Data Fig. 3c). While the biological role of this modification is not known^40^, deletion of *vs* impaired T4 plaque formation specifically in minimal medium (Extended Data Fig. 3d), confirming a fitness defect dependent on nutrient availability. Taken together, these results demonstrate that HIDEN-SEQ can identify conditionally essential genes, including known and previously undescribed factors required by phages to overcome bacterial defence systems and those enabling phage replication at different physiological states of the host.

## HIDEN-SEQ is applicable across different *E. coli* phages

**Fig. 3:**
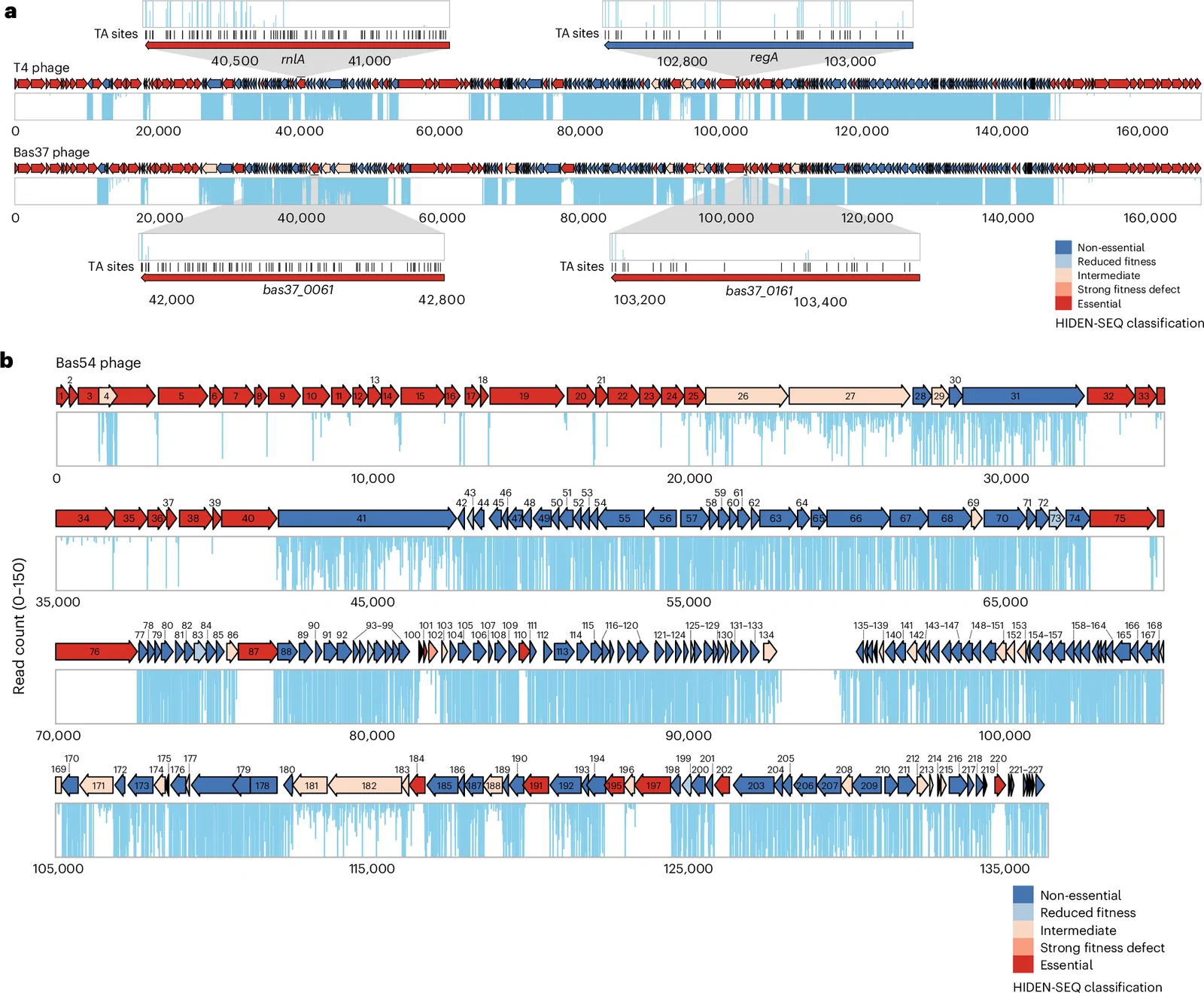
Genome-wide essentiality maps of different *E. coli* phages. **a**, HIDEN-SEQ essentiality maps of T4 and Bas37, two closely related T-even phages, showing broadly similar transposon insertion patterns across both genomes. Zoomed-in views highlight two genes in phage Bas37 that show different insertion patterns compared with their orthologues in phage T4 (Supplementary Data 4 (tabs S3 and S4) and 5 (tabs S3 and S4)). **b**, Phage Bas54 HIDEN-SEQ gene essentiality map with position 1 set to the start of the i-spanin gene at the beginning of the terminase operon. Colour coding as in Fig. 1b (Supplementary Data 6 (tabs S3 and S4)).

**Extended Data Fig. 4:**
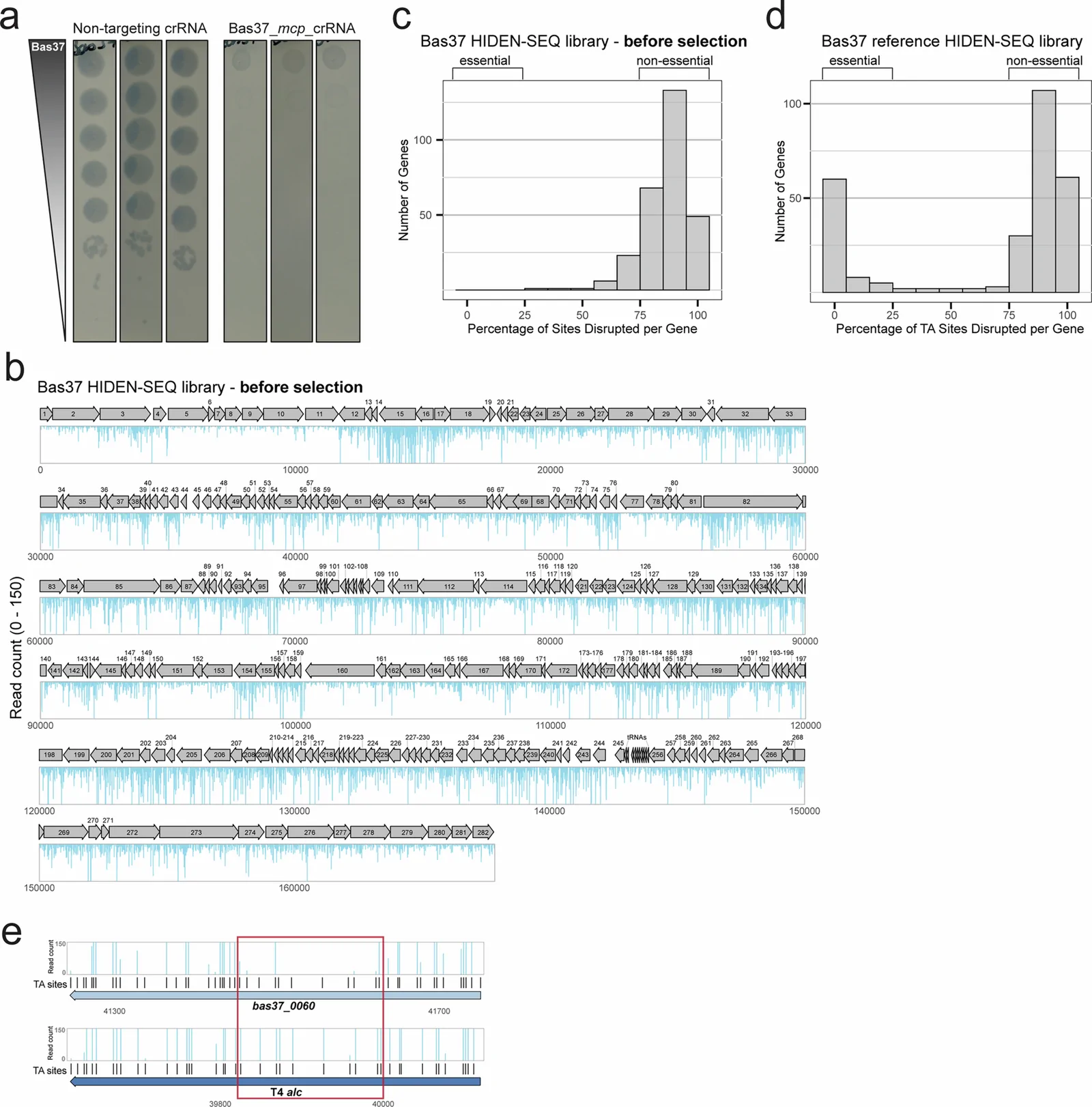
Genome-wide essentiality mapping of Bas37 phage. **a**, Ten-fold serial dilution plaque assays of phage Bas37 plated on *E. coli* K-12 ΔRM expressing *cas13a* with either a non-targeting crRNA control or a crRNA targeting the transcripts of the major capsid protein. **b**, Transposon insertion profiles of the Bas37 input HIDEN-SEQ library collected directly after the transposition step, that is, before strong selection against non-viable insertion mutants by viral replication. The start of the genome map has been set to the start of the small terminase subunit gene. Arrows present the coding sequences with the orientation indicating the direction of transcription. Vertical bars represent the number of transposon insertions at TA dinucleotide sites with heights indicating total read counts (shown up to a maximum of 150). In total, 87.1% of TA sites have been hit. Read counts are not directly comparable across different phage input libraries because the fraction of informative transposon-genome junction reads varies between samples (Methods). **c**, **d**, Histograms illustrating the percentage of disrupted TA sites per gene for the Bas37 input HIDEN-SEQ library dataset (**c**) or the Bas37 reference HIDEN-SEQ library (**d**). The comparison shows that non-essential and essential genes are easily distinguishable in a highly saturated library (Supplementary Data 5 (tab S2 for **b** and **c**; tabs S3 and S4 for **d**)). **e**, Transposon insertion profiles of *bas37_0060* (as an example) and its T4 ortholog *alc* gene highlighting that saturation differences (76% and 97%) shifted the classification to reduced fitness in Bas37 and non-essential in T4.

**Extended Data Fig. 5:**
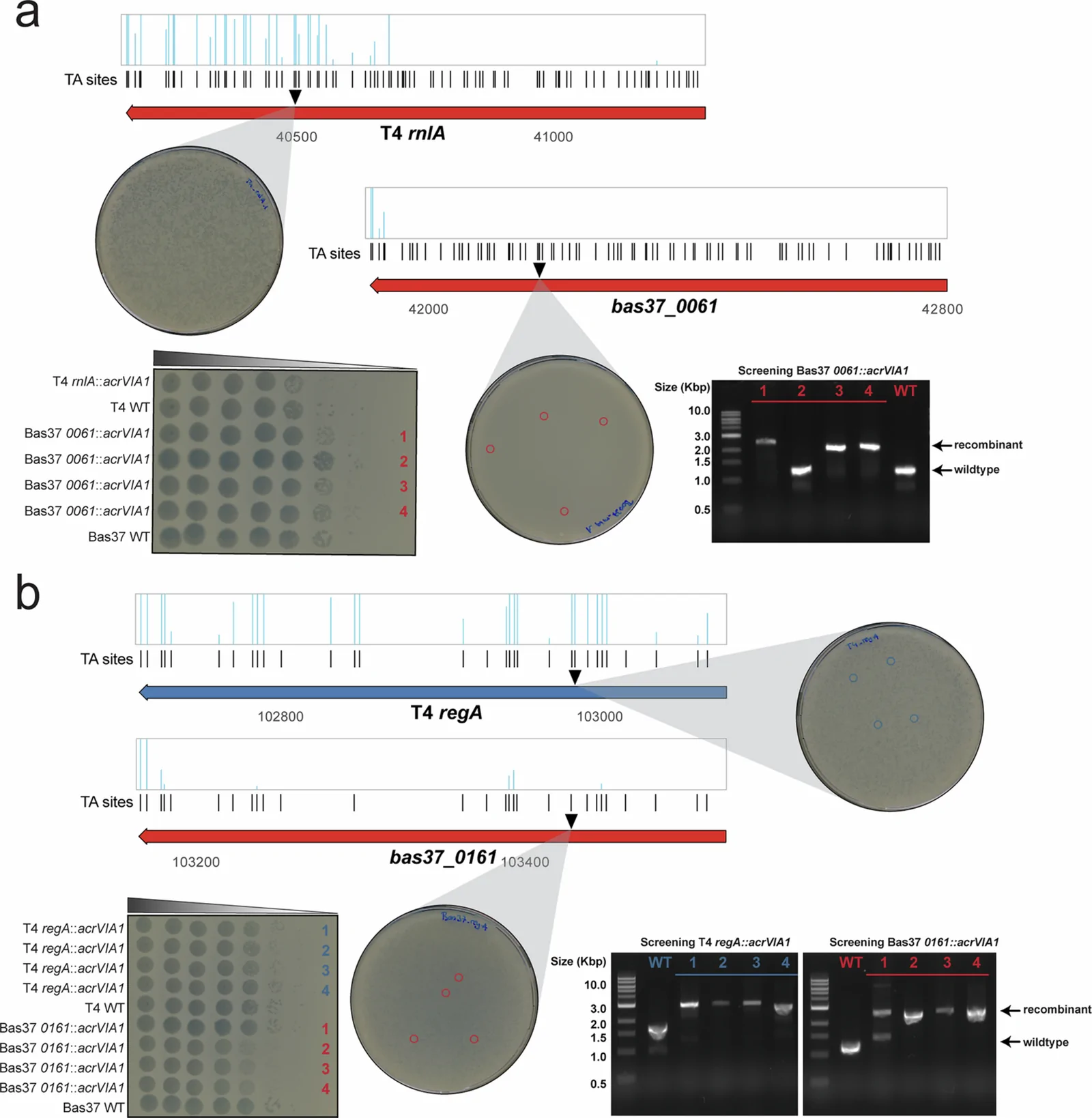
Validation of differential essentiality of *rnlA* and *regA* orthologs in T4 and Bas37. **a**, Transposon insertion profiles across RNA ligase A (*rnlA*) loci in T4 and Bas37 (*bas37_0061*). Vertical bars represent the number of transposon insertions at TA sites (shown with black tick marks), with heights indicating total read counts (shown up to a maximum of 150). The targeted insertion sites are marked by black arrows. Whole-plate infections show recovery of engineered insertion mutants after Cas13a selection, from which individual plaques were isolated and screened by PCR. While clone #2 carries a wildtype-sized *rnlA* locus (possibly with point mutations in the crRNA target sequence), the other clones are insertion mutants. Corresponding spot assays of these mutants are shown together with T4 *rnlA::acrVIA1* site 1 mutant (shown also in Fig. 1c). Bas37 shows markedly reduced recovery of insertion mutants at the *rnlA* locus compared to T4 (see plate images) and poor growth of these mutants as evidenced by smaller plaques of clones #1, #3, and #4. **b**, Transposon insertion profiles across *regA* loci in T4 and Bas37 (*bas37_0161*). Visualization as in (**a**). Bas37 insertion mutants (labelled in red, clones #2-4; note that clone #1 features a wildtype-like band in the PCR) display markedly weaker plaque growth compared to corresponding T4 mutants (labelled in blue, clones #1-4) indicating reduced fitness upon inactivation of *regA* / *bas37_0161* specifically in phage Bas37. Similar results were obtained in independent experiments and results for several independent clones of one experiment are shown in both figure panels. Source data

To explore the potential of HIDEN-SEQ beyond model phage T4, we next applied it to other phages infecting *E. coli*. First, we constructed a highly saturated transposon mutant library of phage Bas37 (Extended Data Fig. 4a–c and Supplementary Fig. 2), a relative of T4^41^, which allowed us to evaluate the robustness of HIDEN-SEQ results by comparing gene essentiality between related phages. Overall, Bas37 and T4 genomes showed highly similar gene essentiality patterns with few minor differences (Fig. 3a and Extended Data Fig. 4d), mostly owing to technicalities of the read count distribution and classification (Extended Data Fig. 4e). Beyond these, the RNA ligase A of Bas37 (*bas37_0061*) showed essentiality across both protein domains, whereas in T4, only the N-terminal domain appeared essential (Fig. 3a). Furthermore, we found that *bas37_0161*, the orthologue of *regA*, a translational repressor of early phage genes, was essential in Bas37 but non-essential in T4 for infection of the laboratory strain *E. coli* K-12. (Fig. 3a). We validated these observations by engineering targeted gene disruptions at the indicated loci, which caused stronger fitness defects in Bas37 than in T4, explaining their depletion in the Bas37 pooled library (Extended Data Fig. 5a,b). These results reveal specific differences in the interaction of T4 and Bas37 with *E. coli* K-12, which could, for example, be informative about different interactions with its repertoire of antiviral defences.

**Extended Data Fig. 6:**
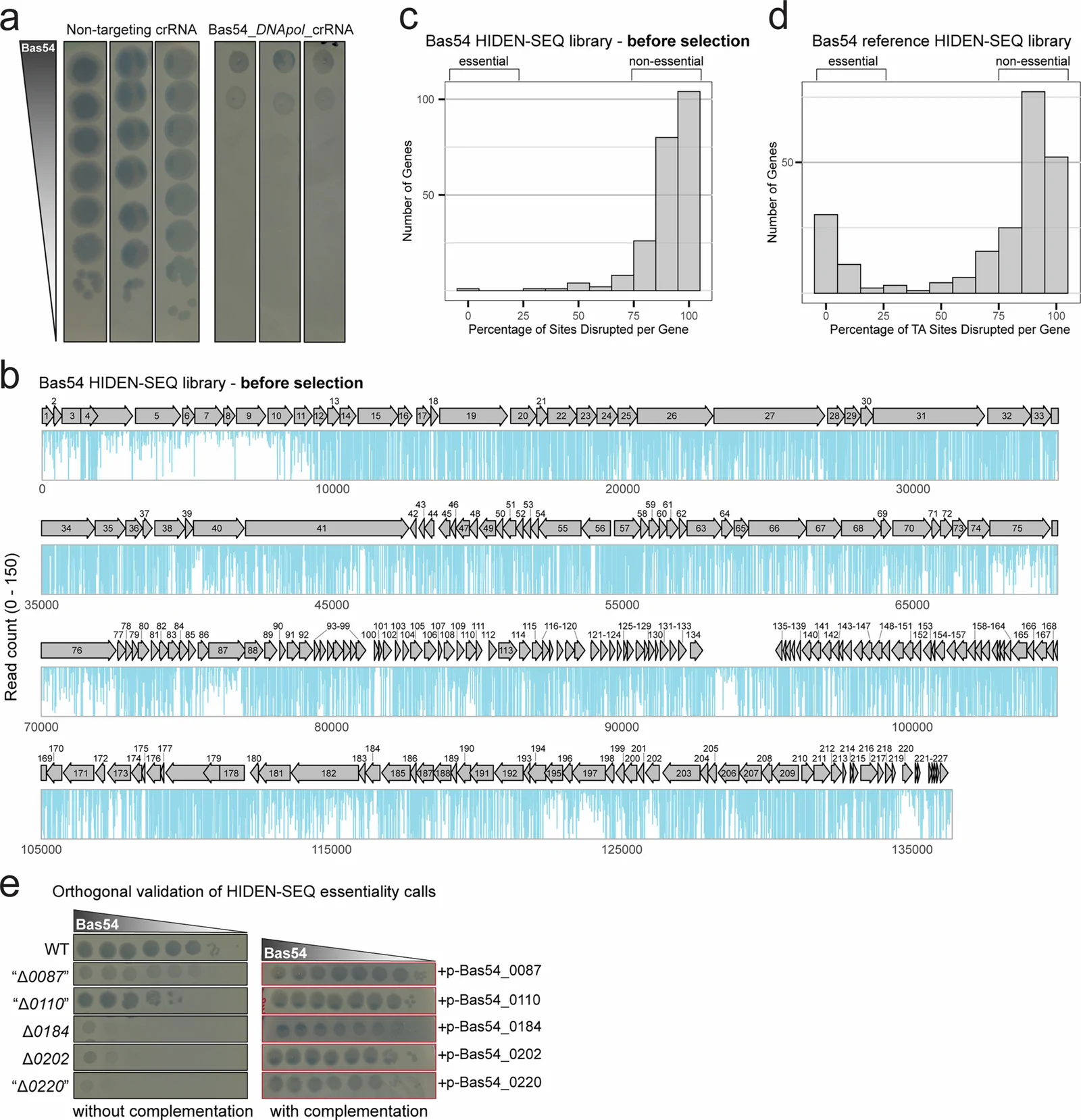
Genome-wide essentiality mapping of Bas54 phage. **a**, Ten-fold serial dilution plaque assays of phage Bas54 plated on *E. coli* K-12 ΔRM expressing *cas13a* with either a non-targeting crRNA control or a crRNA targeting the transcripts of the DNA polymerase gene. **b**, Transposon insertion profiles of the Bas54 input HIDEN-SEQ library collected directly after the transposition step, that is, before strong selection against non-viable insertion mutants by viral replication. The start of the genome map has been set to the start of the i-spanin gene at the beginning of the terminase operon. Arrows present the coding sequences with the orientation indicating the direction of transcription. Vertical bars represent the number of transposon insertions at TA dinucleotide sites with heights indicating total read counts (shown up to a maximum of 150). In total, 91.6% of TA sites have been hit. Read counts are not directly comparable across different phage input libraries because the fraction of informative transposon-genome junction reads varies between samples (Methods). **c**, **d**, Histograms illustrating the percentage of disrupted TA sites per gene for the Bas54 input HIDEN-SEQ library dataset **(c)** or the Bas54 reference HIDEN-SEQ library **(d)**. The comparison shows that non-essential and essential genes are easily distinguishable in a highly saturated library (Supplementary Data 6 (tab S2 for **b** and **c**; tabs S3 and S4 for **d**)). **e**, Ten-fold serial dilution plaque assays of phage Bas54 wildtype and CRISPR-Cas9 knockout mutants (‘Δ*0087*’, ‘Δ*0110*’, Δ*0184*, Δ*0202*, ‘Δ*0220*’) on *E. coli* K-12 ΔRM (left) or *E. coli* K-12 ΔRM expressing the corresponding gene *in trans* (right). For ‘Δ*0087*’, ‘Δ*0110*’ and ‘Δ*0220*’, only mutants carrying larger local deletions could be obtained (see also Supplementary Fig. 3b-d). Wildtype-like plaque formation is restored only upon complementation, confirming the essentiality of these genes. Expression of phage genes from plasmid was induced with IPTG (2 mM, except *bas54_0184*, induced with 0.5 mM due to toxicity). *bas54_0202* encodes for endolysin, while *bas54_0087*, *bas54_0110*, *bas54_0184*, and *bas54_0220* are genes of unknown function. Data are representative of 3 independent biological replicates.

After comparing these two T-even phages, we applied HIDEN-SEQ to Bas54, a phage of the poorly studied *Vequintavirinae* with a genome size of 136-kb encoding 227 predicted genes^41^ (Extended Data Fig. 6a–c). Similar to T4, the Bas54 input HIDEN-SEQ library showed high saturation of TA sites across the genome (91.6%; Extended Data Fig. 6b,c).

Following outgrowth, 145 genes in the Bas54 genome were found to be not essential under standard laboratory conditions, including six genes where disruptions caused markedly reduced fitness (Fig. 3b and Extended Data Fig. 6d). Similar to other *Vequintavirinae* and related phages, Bas54 encodes an extensive set of predicted tail fibres, which has been suggested to contribute to the broad host recognition of this phage group^41,42^. In our HIDEN-SEQ library, several bona fide tail fibre genes (*bas54_0026, bas54_0027, bas54_0028, bas54_0031* and *bas54_0041*) tolerated disruptive insertions, indicating that they are dispensable for infection of *E. coli* K-12 and for the formation of infective virions. Strikingly, 15 of 44 essential genes in the Bas54 genome lack any functional annotation, highlighting that a substantial portion also of the essential genome remains elusive in phages beyond classical models. To validate these findings, we generated knockouts of several of these genes, which indeed caused severe growth defects in the absence of complementation, confirming their essentiality (Extended Data Fig. 6e and Supplementary Fig. 3). These results demonstrate that HIDEN-SEQ is robust and broadly applicable across different viral groups.

## HIDEN-SEQ reveals new antidefence genes in different phages

**Fig. 4:**
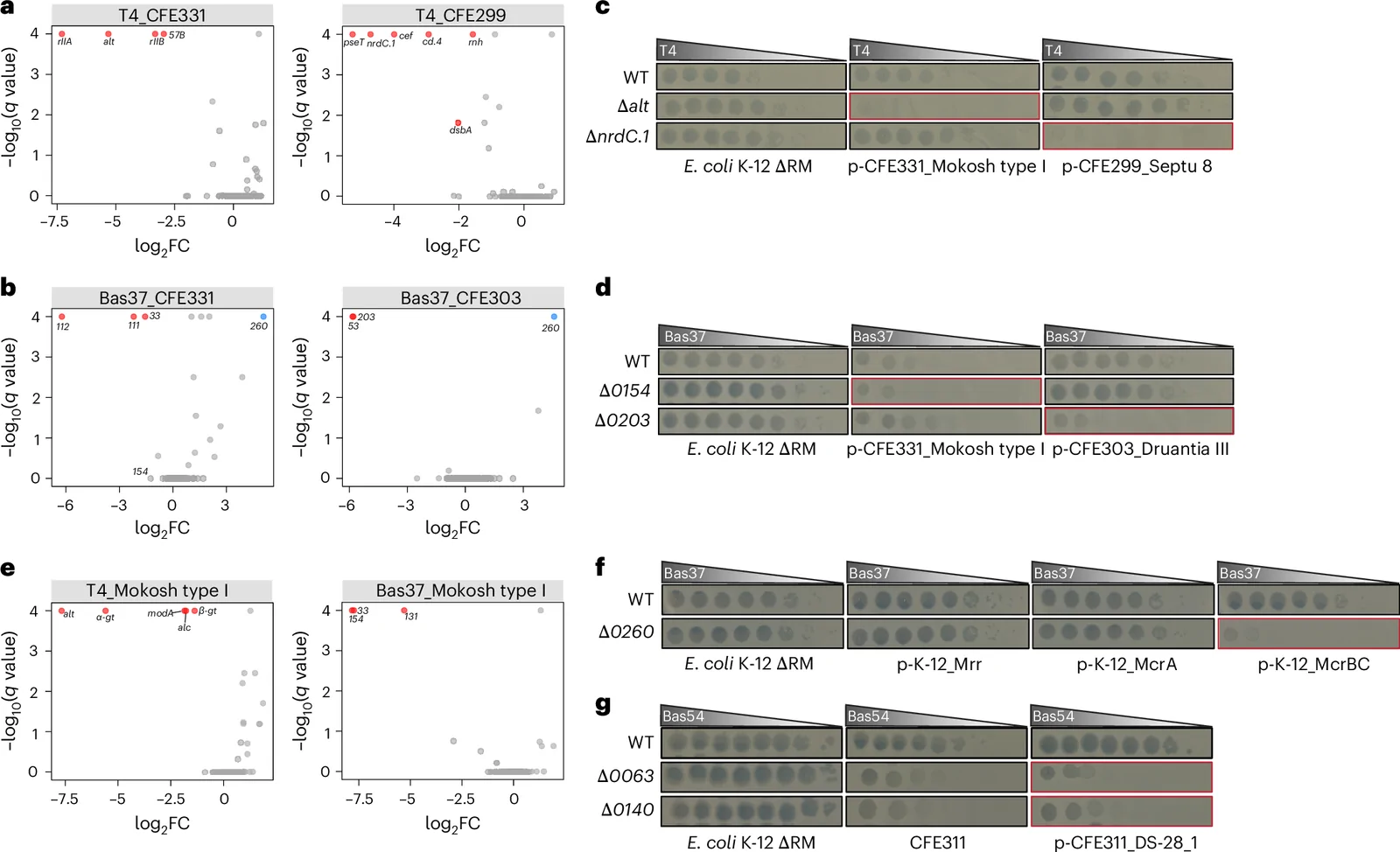
Identification of conditionally essential phage genes in clinical isolates by HIDEN-SEQ. **a**,**b**, Volcano plots comparing the depletion of gene disruptions in phages T4 (**a**) and Bas37 (**b**) after infection of laboratory strain *E. coli* K-12 BW25113 with infections of indicated uropathogenic *E. coli* isolates. Each point represents a viral gene with the *x* axis showing the log_2_FC in normalized insertion counts and the *y* axis showing the −log_10_(*q* value) (Methods). Negative log_2_FC values indicate that mutants with insertions in that gene had reduced fitness in the clinical isolate compared with the laboratory strain. Genes highlighted in red represent the most significant observed effects. In **b**, *bas37_0112* and *bas37_0111* encode RIIA and RIIB orthologues, respectively, and *bas37_0033* encodes for Alt. The single blue point represents disruptions in *bas37_0260*, which cause a strong fitness defect in *E. coli* K-12 BW25113 but not in the clinical isolates. In the Bas37_CFE331 plot, *bas37_0154* is also indicated despite not showing strong depletion because a phenotype with the knockout mutant was observed in the strain expressing the Mokosh type I of CFE331 isolate from a plasmid (Extended Data Fig. 8b and Supplementary Discussion B) (Supplementary Data 4 (tabs S29 and S30 for **a**) and 5 (tabs S15 and S16 for **b**)). **c**,**d**, Tenfold serial dilution plaque assays of T4 (**c**) and Bas37 (**d**) knockout mutants lacking candidate antidefence genes plated on *E. coli* K-12 ΔRM variants expressing predicted defence systems from uropathogenic *E. coli* strains. Data are representative of three independent biological replicates. **e**, Volcano plots comparing the depletion of gene disruptions in phages T4 and Bas37 phage after infection of the *E. coli* K-12 ΔRM with infections of *E. coli* K-12 ΔRM variant expressing the Mokosh type I defence system of *E. coli* CFE331. Each point represents a viral gene; axes as in **a** and **b**. Negative log_2_FC values indicate that mutants with insertions in that gene had reduced fitness in the Mokosh-expressing strain compared with the control. *bas37_0131* encodes the orthologue of ADP-ribosyltransferase ModA (Supplementary Data 4 (tab S31) and 5 (tab S18)). **f**,**g**, Tenfold serial dilution plaque assays of Bas37 (**f**) or Bas54 (**g**) knockout mutants lacking candidate antidefence genes plated on *E. coli* K-12 ΔRM variants expressing any of the three K-12 type IV restriction systems or DS-28 predicted defence system of *E. coli* CFE311. Data are representative of three independent biological replicates.

**Extended Data Fig. 7:**
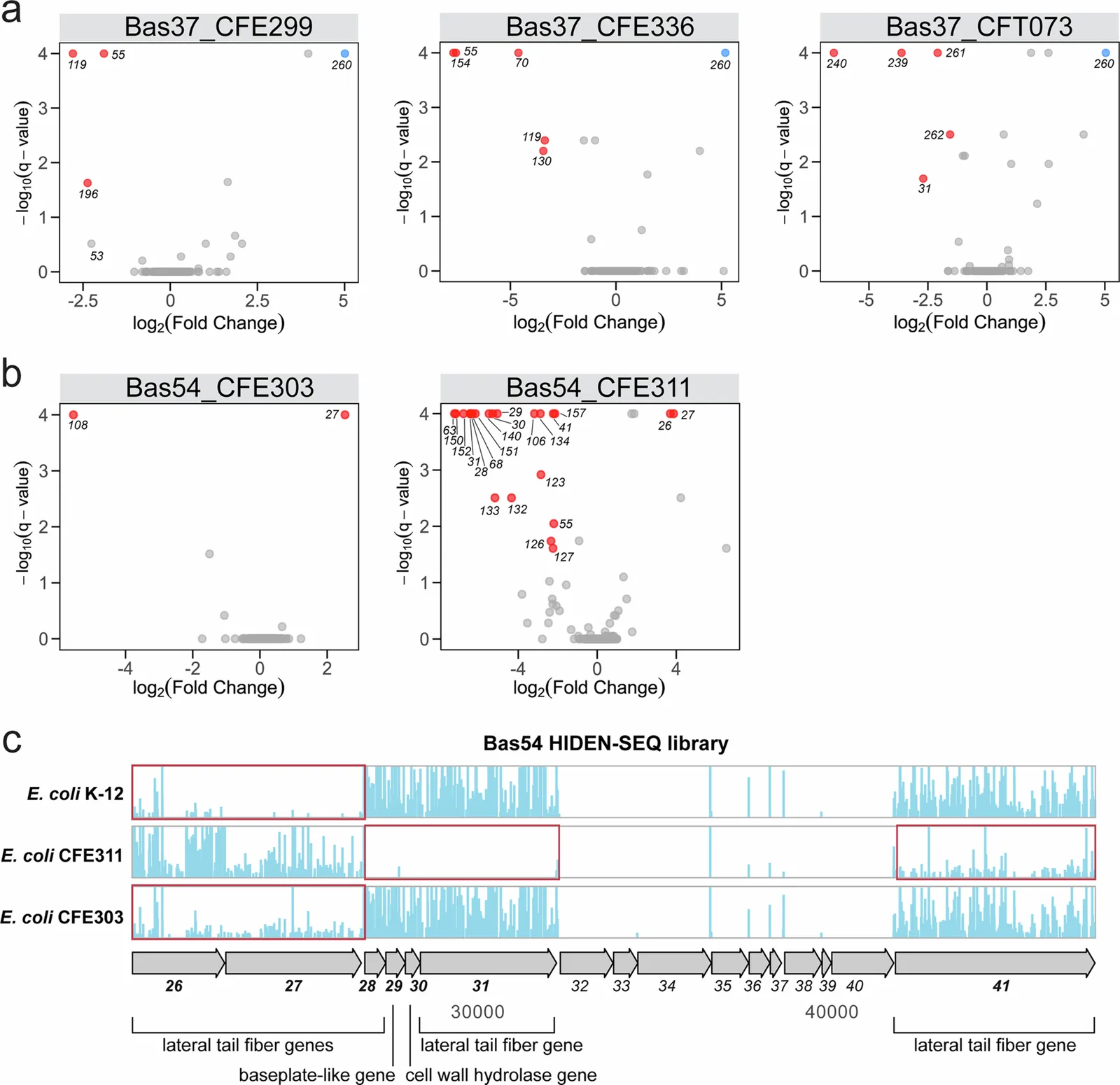
Identification of additional conditionally essential genes in clinical isolates by HIDEN-SEQ. **a**, Volcano plots comparing the depletion of gene disruptions in phage Bas37 after infection of the laboratory strain *E. coli* K-12 BW25113 with infections of uropathogenic isolates *E. coli* CFE299, CFE336, and CFT073. Each point represents a viral gene with the *x* axis showing the log_2_FC in normalized insertion counts and the *y* axis showing the –log_10_(*q* value) (Methods). Negative log_2_FC values indicate that mutants with insertions in that gene had reduced fitness in the clinical isolate compared to the laboratory strain. Genes highlighted in red represent the most significant observed effects. *bas37_0119* encodes the Cef ortholog, *bas37_0196* is a gene of unknown function and NrdC.1 ortholog*, bas37_0055* encodes the PseT ortholog. In all plots, the single blue point represents disruptions in *bas37_0260* which cause a strong fitness defect in *E. coli* K-12 BW25113 but not in the clinical isolates (Supplementary Data 5 (tabs S13, S14 and S17)). **b**, Volcano plots comparing the depletion of gene disruptions in phage Bas54 after infection of the laboratory strain *E. coli* K-12 BW25113 with infections of uropathogenic isolates *E. coli* CFE303 or CFE311. Each point represents a viral gene; axes as in (**a**). Negative log_2_FC values indicate that mutants with insertions in that gene had reduced fitness in the clinical isolate compared to the laboratory strain. Genes highlighted in red represent the most significant observed effects (Supplementary Data 6 (tabs S8 and S9)). **c**, Transposon insertion profiles at the locus encoding lateral tail fibre genes of Bas54 obtained after infection of *E. coli* K-12 BW25113, *E. coli* CFE311, and *E. coli* CFE303 with the Bas54 reference HIDEN-SEQ library. Vertical bars represent the number of transposon insertions at TA sites with heights indicating total read counts (shown up to a maximum of 150). It is apparent that different sets of lateral tail fibre genes are conditionally essential for growth on different host strains (Supplementary Data 6 (tabs S5–S7)).

A major advance of HIDEN-SEQ is its ability to assign new functions to the uncharacterized ‘dark matter’ of phage genes by conditional essentiality. To test this in a clinically relevant setting, we infected a panel of *E. coli* isolates from urinary tract infections (Supplementary Table 1) with T4, Bas37 and Bas54 transposon libraries to compare viral gene essentiality across hosts. Intriguingly, for each phage–host pair, we identified multiple viral genes with strongly reduced insertion counts relative to the reference strain, indicating conditionally essential roles (Fig. 4a,b and Extended Data Fig. 7a,b). While these genes may contribute to diverse viral functions during infection, many are probably involved in overcoming bacterial immunity, which is highly variable across *E. coli* strains^43^. However, some lateral tail fibre genes of Bas54 also showed host-specific conditional essentiality, suggesting that this phage deploys alternative tail fibres to target different host receptors (Extended Data Fig. 7c).

**Extended Data Fig. 8:**
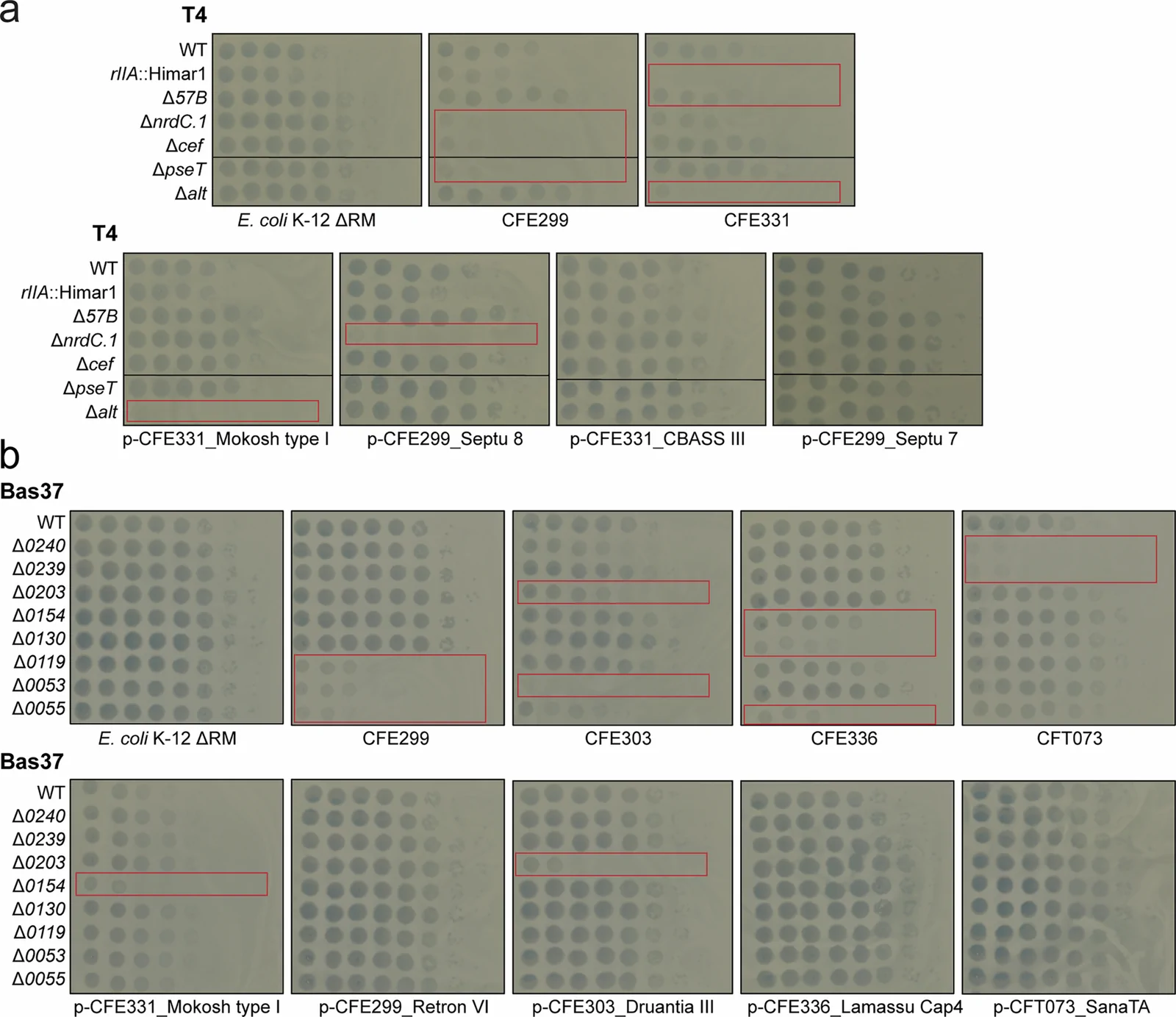
Matching candidate phage antidefence genes identified by HIDEN-SEQ with host defence systems. The figure shows ten-fold serial dilution plaque assays of knockout mutants of phages T4 (**a**) and Bas37 (**b**) lacking candidate antidefence genes plated on *E. coli* K-12 ΔRM variants expressing predicted defence systems cloned from uropathogenic *E. coli* strains. In parallel, the same viral knockout mutants were also spotted on the uropathogenic strains themselves to confirm the fitness defects detected by HIDEN-SEQ (see Fig. 4a, b and Extended Data Fig. 7a). Data are representative of 3 independent biological replicates.

We first focused our follow-up experiments on the T-even phages, which are known to harbour a diverse repertoire of antidefence factors^44,45^ and showed overlapping sets of conditionally essential genes on the same clinical strains (Fig. 4a,b and Extended Data Fig. 7a). To link these candidate antidefence genes to specific branches of bacterial immunity, we used DefenseFinder^46^ to predict defence genes in these hosts and selected some defence systems for further analysis. In parallel, we generated targeted deletions of a subset of the most strongly depleted phage genes (log_2_ fold change, log_2_FC <−2). We then challenged each phage mutant with each cloned defence system expressed ectopically in *E. coli* K-12, which revealed multiple viral antidefence genes acting against different bacterial defence systems, including both previously undescribed factors and genes with known functions (Fig. 4c,d and Extended Data Fig. 8a,b).

**Extended Data Fig. 9:**
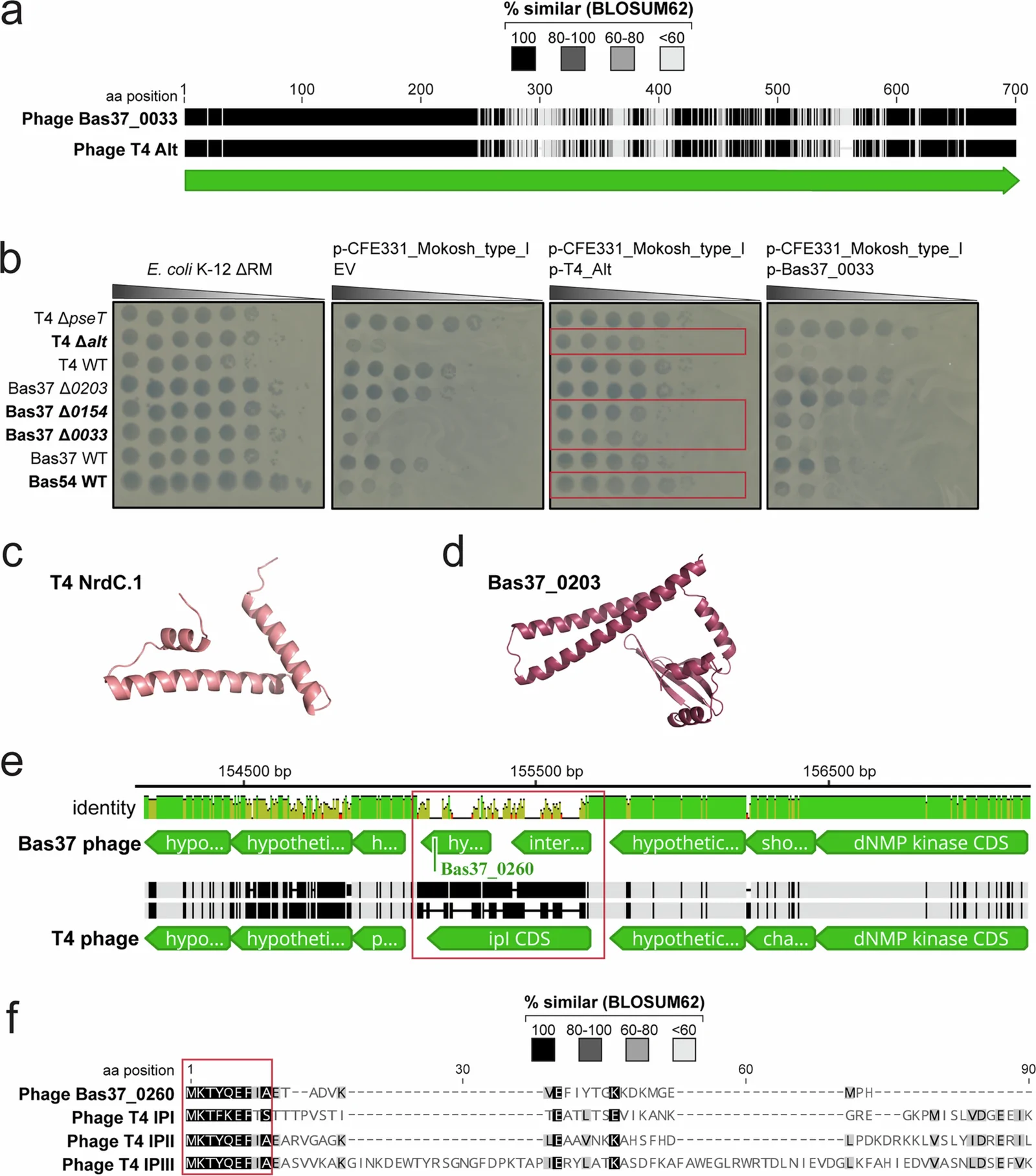
New antidefence genes with known and unknown functions. **a**, Amino acid sequence alignment of Alt ADP-ribosyltransferase of phage Bas37 (encoded by *bas37_0033*) and phage T4 generated using MAFFT v7.490 implemented in Geneious Prime 2023.0.4 with default settings. **b**, Ten-fold serial dilution plaque assays of knockout mutants of phages T4 and Bas37 plated on *E. coli* K-12 ΔRM expressing the Mokosh type I with either an empty vector (EV) or expressing the Alt orthologs of T4 or Bas37 (encoded by *bas37_0033*). Plates show infections with T4 Δ*alt*, Bas37 Δ*0033*, Bas37 Δ*0154* (which lacks β-α glucosyltransferase), and the Mokosh-sensitive phage Bas54 after induction with 5 mM IPTG. Wildtype phages and knockout mutants T4 Δ*pseT* and Bas37 Δ*0203* are shown as controls. Data are representative of 3 independent biological replicates. **c**, AlphaFold2-predicted structure of NrdC.1 of phage T4. **d**, AlphaFold2 predicted structure of Bas37_0203 of phage Bas37. **e**, The illustration shows a sequence alignment at the region comprising the *ipI* of phage T4. Note that the upstream and downstream regions are highly conserved. T4 encodes the *ipI* gene, whereas Bas37 codes for two different genes in this locus, including *bas37_0260*. Alignments were generated using MAFFT v7.490 implemented in Geneious Prime 2023.0.4 with default settings. **f**, Partial amino acid sequence alignment of Bas37_0260 and phage T4 internal proteins IPI, IPII, IPIII, highlighting the conserved sequence motif at the N-terminus.

One notable finding was that Alt of T4 phage and β-α glucosyltransferase (*βα-gt*; encoded by *bas37_0154*) of Bas37 are involved in evasion of the Mokosh type I defence system of *E. coli* strain CFE331 (Fig. 4c,d). The *alt* gene encodes an ADP-ribosyltransferase that has been previously reported to manipulate the host RNA polymerase via ADP-ribosylation^47^ and to interfere with the MazEF toxin–antitoxin system^48^. Interestingly, the Alt orthologues of T4 and Bas37 exhibit substantial sequence differences (Extended Data Fig. 9a). When expressed ectopically in the presence of Mokosh type I, only Alt^T4^, but not Alt^Bas37^ (Bas37_0033), partially restored plaque formation of the T4 *alt* mutant, Mokosh-sensitive Bas37 mutants and even the naturally Mokosh-sensitive phage Bas54 (Extended Data Fig. 9b). Additional HIDEN-SEQ experiments on *E. coli* K-12 ectopically expressing Mokosh type I confirmed that T-even phages evade this defence system using both their ADP-ribosyltransferases and viral DNA glucosylation (Fig. 4e and Supplementary Discussion B).

We also found that the previously uncharacterized T4 *nrdC.1* and Bas37 *bas37_0203* genes encode bona fide antidefence factors targeting the Septu and Druantia type III defence systems, respectively (Fig. 4c,d and Extended Data Fig. 8a,b). These proteins lack any known domains or characterized homologues (Extended Data Fig. 9c,d), so their mechanisms of action remain elusive. Another interesting finding relates to *bas37_0260*, which is encoded at the same locus in Bas37 as the T4 inhibitor of the GrmSD type IV restriction system called internal protein I (IPI)^49^ (Extended Data Fig. 9e). Disruption of *bas37_0260* caused a strong fitness defect when infecting the *E. coli* K-12 BW25113 laboratory strain but not different uropathogenic isolates or the nearly identical *E. coli* K-12 MG1655 ΔRM variant in which we had previously deleted the three type IV restriction systems McrA, McrBC and Mrr^41^ (Fig. 4b and Extended Data Fig. 7a). A *bas37_0260* knockout indeed became specifically sensitive to McrBC (Fig. 4f), demonstrating that Bas37_0260 is a previously unknown type IV restriction inhibitor with no homology to its analogues IPI, IPII and IPIII of phage T4 beyond the characteristic N-terminal signal sequence that directs its packaging into virions^50^ (Extended Data Fig. 9f).

Lastly, we extended the screen pairing phage genes and host defences also to phage Bas54 and identified two antidefence genes specifically required to overcome DS-28, a predicted defence system encoded by *E. coli* strain CFE311 (Fig. 4g). These two genes are *bas54_0063* encoding a MoxR ATPase and *bas54_0140* with no functional annotation. Together, these findings show that HIDEN-SEQ can systematically uncover phage genes that counteract bacterial defences, including those with no prior annotations or characterized function.

## Discussion

In this study, we have established HIDEN-SEQ as a genome-wide TnSeq method for bacteriophages. This method allows systematic disruption of phage genes by Himar1 transposition and selection for transposon mutants with a CRISPR–Cas13a/anti-CRISPR system. Using HIDEN-SEQ, we constructed densely saturated transposon libraries for three different phages that allowed us to systematically map phage gene essentiality at a very high resolution. We benchmarked the technique using the well-studied T4 model phage, where most genes are dispensable under standard laboratory conditions, and show that HIDEN-SEQ reliably recapitulates its known biology^10^. At only a few loci, disruptions were not tolerated even though previous work had proposed that the affected genes may be not essential, possibly owing to polar effects or differences in the techniques used to assess gene essentiality (Supplementary Discussion A). Importantly, the high saturation of our input library (Extended Data Fig. 1b) confirms that these observations are not caused by inefficient transposition and that post-selection depletion reflects real fitness costs of the insertions.

Beyond T-even phages, HIDEN-SEQ revealed many genes of unknown function with essential roles in non-model phage infection, highlighting its potential to explore gene function across diverse phage groups. A major strength of this method is its ability to uncover conditionally essential genes that become critical only under certain challenges. HIDEN-SEQ not only identified known antidefence genes in the presence of their corresponding bacterial defence systems but also uncovered new bona fide factors acting against six distinct defence systems (DarTG2, Mokosh type I, Septu, Druantia type III, McrBC type IV restriction and DS-28). Five of these viral genes had no previously known function, demonstrating the great potential of HIDEN-SEQ screens to assign functions to the ‘dark matter’ of phage genomes.

Several approaches for mapping phage gene essentiality have recently expanded the toolbox for phage biology and feature specific disadvantages compared with HIDEN-SEQ^15–20^. The knockdown-based CRISPRi-ART^16^, for instance, relies on previous knowledge about the position of viral genes and accurate start codon prediction while also being constrained by the notorious variability of crRNA efficacy across genes and conditions. Consequently, this method failed to detect the essentiality of around 25% of the known essential genes of phage T4. However, knockdown approaches such as CRISPRi^15,16^ or ASO-based methods^20^ are suited to probe conditional fitness effects of essential genes that are inherently inaccessible to HIDEN-SEQ unless transposition would be performed in vitro. Another recently published method, PhageMaP, demonstrated that phage gene essentiality can be mapped by targeted mutagenesis^17^. However, this is a laborious approach that requires extensive custom construct design for each phage and generates only a few insertional mutants at predefined sites per gene, which introduces bias and limits resolution. By contrast, HIDEN-SEQ requires no prior knowledge about genes in the target phage, relies on a single crRNA for selection per phage and generates high-density insertion libraries that can be repeatedly used across different hosts and conditions. During the revision of this manuscript, two independent studies reported related TnSeq approaches applied to phages of *Pseudomonas* and *Prodigiosinella*^32,51^. Together with HIDEN-SEQ, these studies show that phage transposon mutagenesis is broadly applicable, while also illustrating that the biology of individual phages may require tailored adjustments of the technology, such as phage-specific promoters or alternative transposon systems to overcome DNA modifications.

Taken together, HIDEN-SEQ provides a versatile platform that links phage phenotypes to underlying genes and, consequently, molecular mechanisms. It enables high-throughput mapping of phage–host interactions beyond standard laboratory conditions, including the infection of clinical isolates and under varied growth environments. In addition, HIDEN-SEQ reveals dispensable genome regions that can be directly informative for phage engineering to improve clinical and biotechnological applications. Looking forward, its great utility and efficacy make HIDEN-SEQ a powerful tool to address complex biological questions about phage biology with far-reaching implications for phage therapy, microbial ecology and biotechnological innovation.

## Methods

### Preparation of culture media and solutions

LB culture medium was prepared by dissolving 10 g l^−1^ tryptone, 5 g l^−1^ yeast extract and 10 g l^−1^ sodium chloride in Milli-Q H_2_O and then sterilized by autoclaving. LB agar plates were prepared by supplementing LB medium with 1.5% w/v agar before autoclaving. M9 minimal medium supplemented with 0.4% w/v D-glucose (M9Glc) was prepared as previously described^52^. M9Glc agar plates were prepared by mixing autoclaved 3% (w/v) agar and sterile 2× M9Glc medium in a 1:1 ratio to achieve a final agar concentration of 1.5%. Phosphate-buffered saline (PBS) was prepared as a solution containing 8 g l^−1^ NaCl, 0.2 g l^−1^ KCl, 1.44 g l^−1^ NA_2_HPO_4_·2H_2_O and 0.24 g l^−1^ KH_2_PO_4_, adjusted to pH 7.4 with 10 M NaOH and sterilized by autoclaving. SM buffer was prepared as 0.1 M NaCl, 10 mM MgSO_4_ and 0.05 M Tris (pH 7.5) and was also sterilized by autoclaving.

### Bacterial strain handling and culturing

*E. coli* strains were routinely cultured in LB medium at 37 °C with agitation at 170 rpm. For experiments involving the DarTG2 system, the bacteria were instead cultured in M9Glc medium and incubated at 30 °C (ref. ^53^). We selected for plasmid maintenance using ampicillin at 50 µg ml^−1^, chloramphenicol at 25 µg ml^−1^, gentamicin at 20 µg ml^−1^ and kanamycin at 50 µg ml^−1^. A full list of bacterial strains used in this study is presented in Supplementary Table 1.

### Isolation of new uropathogenic *E. coli* strains

Urine samples from patients with suspected asymptomatic bacteriuria or urinary tract infection were collected at the Department of Urology and the Emergency Department at the University Hospital Basel (Switzerland). This work was part of a study approved by the ethical commission of northwest and central Switzerland (EKNZ, approval no. 2020_02588). A detailed overview of the study design/protocol is provided on ClinicalTrials.gov (registered as NCT05017766). Patients were included in accord with this approved study protocol. Urine specimens (8 ml) supplemented with boric acid were submitted to the Clinical Microbiology Laboratory of the University Hospital Basel for routine diagnostic culture and identification of uropathogenic *E. coli* (UPEC). In brief, 10 μl urine was plated onto 5% sheep blood agar plates and incubated under standard clinical microbiology conditions. Following confirmation of UPEC-positive cultures, single colonies were isolated and stored at −80 °C using cryopreservation beads. On request, strains of interest were restreaked on 5% sheep blood agar plates and transferred to Biozentrum (University of Basel, Switzerland) for genomic DNA extraction and subsequent genome sequencing.

### Bacteriophage handling and culturing

Bacteriophages were cultured using the double-agar overlay (‘top agar’) method^54^. Top agar was prepared as LB agar with 0.5% w/v agar and supplemented with 20 mM MgSO_4_ and 5 mM CaCl_2_. Top agar plates were incubated at 37 °C or 30 °C (Δ*adfM* T4 phage experiments) for approximately 16–20 h before plaque enumeration. For CRISPR–Cas13a counterselection, top agar was made without divalent cations and supplemented with chloramphenicol at 25 µg ml^−1^ and anhydrotetracycline (aTc) at 5 nM to induce the expression of Cas13a^30^. For transposition experiments, 2 mM isopropyl-β-D-thiogalactopyranoside (IPTG) was added to the top agar to induce transposase expression. When M9Glc plates were used, top agar was prepared by mixing sterile 1% w/v agar with 2× M9Glc medium in a 1:1 ratio, to have a final agar concentration of 0.5% w/v and maintain equivalent nutrient composition to the M9Glc agar plate. All bacteriophages used in this study are listed in Supplementary Data 1.

### Plasmids and plasmid construction

Plasmids were typically constructed using the method of Gibson et al.^55^ (‘Gibson Assembly’) by ligating PCR products containing 25 nucleotide overlaps. Some plasmids have been constructed using classical restriction–ligation cloning as described in our previous work^41^. Mutations in plasmids were introduced by round-the-horn PCR^56^ with partially overlapping primers. We used a two-plasmid setup for LbuCas13a counterselection, where we separated Cas13a protein expression into a dedicated plasmid pAH221 (derivative of all-in-one plasmid pBA559^30^; Addgene 186235) and cloned the crRNA expression cassette into pAH218 (RSF1010 origin of replication). The same two-plasmid setup was used to establish the LseCas13a system in *E. coli*. The coding sequence of the LseCas13a protein was codon-optimized for *E. coli* and synthesized by Twist Bioscience before cloning into the pAH221 backbone. In parallel, the crRNA expression cassette was cloned into the pAH218 backbone. The crRNAs for selection against wild-type phages were designed to target essential phage transcripts, using either 31 (LbuCas13a) or 24 nucleotide (LseCas13a) spacers^28,30^ and cloned into the pAH218 backbone via Golden Gate assembly^30^. Codon-optimized gene sequences for the anti-CRISPRs, AIcrVIA3 (LbuCas13a)^57^ and AcrVIA1 (LseCas13a)^27^, were synthesized (Twist Bioscience) and cloned into the transposon and homologous recombination vectors under the control of constitutive promoters from the Anderson library (J23110 for AIcrVIA3; J23119 for AcrVIA1). Transposon plasmids (RSF1030 high-copy origin of replication) contained mariner transposon inverted repeats (GGGGACTTATCAGCCAACCTGTTATGT) flanking the selection marker. The transposase gene was cloned into pAH186, a ColE1-based plasmid with IPTG-inducible expression. Defence systems were predicted in the genomes of the *E. coli* clinical isolates using DefenseFinder (v2.0.0)^46^ (Supplementary Data 2) and then cloned on a SC101-*ori* plasmid under their native promoter (by including a >80-bp upstream region) and used to transform *E*. *coli* K-12 MG1655 ΔRM^41^ for further testing. For phage gene complementation, the pNDM220 backbone was used, which contains an IPTG-inducible *Plac* promoter on a mini-R1 backbone. All cloned constructs were routinely verified by Sanger sequencing or full plasmid sequencing using Oxford Nanopore technology at Microsynth AG. The construction of all plasmids is described in detail in Supplementary Data 3. All oligonucleotide primers and synthesized DNA fragments used in this work are presented in Supplementary Table 2.

### Construction of phage mutants

Unless stated otherwise, knockout mutants of bacteriophage genes and other phage mutants were constructed by introducing an *acr* gene at the target locus through homologous recombination followed by CRISPR–Cas selection as pioneered by Guan and colleagues^28^. In brief, the *acr* gene was cloned with flanking >100-bp phage homology arms onto a replicative plasmid as recombination donor. Subsequently, an *E. coli* K-12 host was transformed with this plasmid and infected with the target phage to enable homologous recombination. The lysate from this infection was then subjected to CRISPR–Cas13a selection against the parental wild-type phage using a crRNA targeting the transcripts of an essential phage gene. Subsequently, plaques were screened for successful insertion of the *acr* gene by overspanning PCR, and the sequence of the recombinant phage was verified by Sanger sequencing at the relevant locus. The phage mutant isolates were passaged three times via single plaques on top agar of the Cas13a selection strain for clonal purification. In attempts to generate a T4 knockout mutant of the *wac* gene, homologous recombination and CRISPR–Cas13a selection were performed either without or with genetic complementation from the IPTG-inducible pNDM220 plasmid (Supplementary Fig. 4).

### Construction of Bas54-knockout mutants with genetic complementation

To orthogonally validate HIDEN-SEQ essentiality calls, knockout mutants of selected Bas54 genes were generated by homologous recombination followed by CRISPR–Cas9 counterselection^58^. Single-guide RNAs (sgRNAs) were designed using Geneious Prime 2023.0.4 and their predicted activity was assessed according to Doench et al.^59^. To prevent targeting of the complementation vector, sgRNAs were selected to target regions outside the expressed sequence: For *bas54_0009*, *bas54_0087*, *bas54_0184* and *bas54_0202*, the sgRNAs targeted the 3′ region surrounding the stop codon, whereas for *bas54_0110* and *bas54_0220*, they targeted the 5′ region near the start codon. sgRNAs were constitutively expressed from a p15A *ori* backbone. For homologous recombination, ~250-bp upstream and downstream homology arms flanking the target locus were cloned into an SC101-*ori* plasmid. The corresponding phage gene was provided in trans from pNDM220 plasmid (mini-R1 *ori*) during both recombination and CRISPR–Cas9 selection. Bas54 was first propagated on the recombination template strain and the resulting lysate was subsequently plated on a strain carrying the CRISPR–Cas9 system with the guide targeting the gene to be deleted. Individual plaques were screened by overspanning PCR and verified with Sanger sequencing or whole-genome sequencing.

### Construction of the T4 HIDEN-SEQ library

The transposition donor strain was obtained by sequentially transforming *E. coli* K-12 with two plasmids. The first plasmid encoded the Himar1 transposase under the control of an IPTG-inducible promoter, while the second carried the mariner transposon in trans on a high-copy plasmid to bypass the notorious overexpression inhibition of mariner-type transposases^60^. For the T4 reference HIDEN-SEQ library, the transposon encoded the *acrVIA1* gene without a transcriptional terminator to allow readthrough transcription. It also included stop codons in all three reading frames at the 3′ end of the *acrVIA1* gene to prevent aberrant translation. To assess potential polar effects, we also constructed a T4 HIDEN-SEQ library using the same transposon with *rrnB* T1 transcriptional terminator inserted downstream of *acrVIA1*. To generate a transposon pool, phage T4 was grown to confluency on top agar plates of the transposon donor strain supplemented with IPTG for transposase induction. Confluent plaques from these plates were scraped and pooled in 10–15 ml SM buffer and then centrifuged at 8,000*g* for 10 min. Supernatants were collected and titre were determined, typically yielding >10^10^ plaque-forming units (PFU) ml^−1^. Even at possibly very low transposition frequencies such as 1 in 10^5^ plaques, at least ≥10^5^ PFU ml^−1^ would be transposon mutants, which is well above the ~15,000 unique insertions needed to potentially saturate all 14,474 TA sites in the genome of our T4 laboratory variant (Supplementary Data 1). The resulting lysate (referred to as the input library) was then plated on top agar with the selection strain expressing LseCas13a and a potent crRNA targeting the transcripts of the major capsid protein (*mcp*). As a control, a parallel lysate was prepared using a donor strain carrying a non-selectable transposon without anti-CRISPR and plated on the same selection strain to estimate the frequency of Cas13a genetic escape. We tested different volumes of the phage lysate for plating to obtain single plaques with adequate spacing where direct competition between mutants may be reduced. Plaques were counted to estimate the number of single plaques per square plate, which was used to calculate the number of plates required to make the library. We aimed to collect at least twice as many plaques as there are TA dinucleotide sites in the phage genome to ensure high saturation. Plaques from all plates were scraped and pooled in 100–200 ml of SM buffer and centrifuged at 8,000*g* for 10 min. The resulting final stock of the library had a titre of 10^9^–10^10^ PFU ml^−1^, predominantly transposon mutants with a possible minor fraction of genetic escape mutants. The library was stored at 4 °C until further use. Following the same protocol, another T4 library was later constructed on the *E. coli* Δ*rnlA* strain from the Keio collection^61^, which does not express the RnlA–RnlB system, to test the conditional essentiality of *dmd* (Fig. 2b).

### Construction of Bas37 HIDEN-SEQ library

The Bas37 HIDEN-SEQ reference library was constructed using the same procedure as for T4. For counterselection, we used the same crRNA targeting the major capsid protein, which was also effective against Bas37 because the target sequence is conserved between these two closely related phages^41^. We collected a similar number of plaques aiming to saturate the 14,279 TA dinucleotide sites in the Bas37 genome^41^.

### Construction of Bas54 HIDEN-SEQ library

The Bas54 HIDEN-SEQ reference library was generated using the same procedure described above with a simple adaptation of the selection system to this virus, allowing us to evaluate the flexibility of HIDEN-SEQ. For Bas54, the transposon encoded the anti-CRISPR gene *AIcrVIA3*, and selection was consequently performed using the LbuCas13a system with crRNA targeting the transcripts of the DNA polymerase gene. We pooled more than 17,000 plaques to cover 8,585 TA dinucleotide sites in the Bas54 genome^41^, ensuring high saturation of the transposon library.

### Competitive fitness assay using HIDEN-SEQ library

Overnight cultures of the target hosts were diluted 1:10 to mid-exponential phase (approximately 5 × 10^8^ colony-forming units (CFU) ml^−1^) in fresh LB medium with additional supplements if necessary. The HIDEN-SEQ reference phage library was added at a multiplicity of infection (MOI) of 1:100 to ensure single infections. At this MOI, infections are performed with 5 × 10^6^ PFU ml^−1^, which ensures that there are more phages per millilitre than the transposon mutant pool can contain independent mutants. This excess robustly buffers the experiment against random perturbations of mutant abundance. After agitation at 170 rpm in Erlenmeyer flasks for 20–24 h at 37 °C, the cultures were centrifuged at 8,000*g* for 10 min and the supernatants were transferred to fresh tubes. Mutant library phage lysates were stored at 4 °C before genomic DNA extraction.

Condition-specific modifications were applied when necessary: for CBASS experiments, 0.2% L-arabinose was added at the time of infection to induce expression of defence genes^35^. Competitive fitness assays to test growth medium-dependent conditional essentiality were performed in both LB and M9Glc medium. For experiments with the DarTG2 system, infections were performed in M9Glc medium with incubation at 30 °C (ref. ^53^). Further details of all libraries generated in this work are provided in Supplementary Data 4–6.

### HIDEN-SEQ library preparation and sequencing

Genomic DNA was extracted from phage transposon mutant library samples using the Norgen Biotek Phage DNA Isolation Kit. Library preparation and sequencing were performed at the Lausanne Genomic Technologies Facility (GTF) using the AVITI short-read sequencer (Element Biosciences), following a customized protocol. In brief, genomic DNA was sheared to ~300–500-bp fragments and ligated to Illumina-compatible adaptors containing i5 and i7 indexes. A first round of PCR was performed using a transposon-specific primer with a 5′ biotin label and an adaptor-specific primer to selectively amplify transposon insertion sites. Biotinylated PCR products were captured using streptavidin-coated magnetic beads to remove background genomic DNA. Finally, to enrich transposon-insertion sites, another PCR was performed using a second transposon-specific primer that anneals close to the transposon-genome junction and incorporates the P5 flow cell adapter. The resulting libraries were deep sequenced using single-end sequencing. Input HIDEN-SEQ libraries, collected before outgrowth and CRISPR–Cas13a selection, were more difficult to sequence because transposon-specific amplification also captured residual donor plasmid DNA, reducing the fraction of reads derived from transposon–genome junctions and probably underestimating insertion density. To improve recovery of informative reads from these samples for the identification of transposon insertion sites, the DNA library preparation workflow was manually scaled up and optimized for maximal amplification. These libraries were sequenced only to assess the efficiency of transposition and overall library saturation before outgrowth. All analyses for gene essentiality were based on libraries obtained after outgrowth/selection and not these input libraries.

### Data analysis of HIDEN-SEQ libraries

Raw sequencing reads were initially assessed for quality using FastQC (v0.12.1)^62^. Adapter trimming and quality filtering were subsequently performed with fastp (v0.22.0)^63^, applying a minimum Phred quality score cutoff of Q30 and discarding reads shorter than 50 bp. Owing to the variable density of TA sites across different phages, we implemented a random read subsampling strategy using seqkit (v2.10.0)^64^ to standardize the analysis across different datasets. Specifically, for each phage, we estimated a target sequencing depth of 350 reads per TA site, which maintains the library complexity and ensures that the global sequencing depth is normalized across genomes with different insertion site availability. The preprocessed reads were then used to identify transposon insertion sites with the TPP tool from TRANSIT (v3.3.13) software^65^. TPP first detects a match (allowing no mismatch) to the transposon prefix in each read and then maps the genomic portion to the phage reference genome using BWA (v0.7.12)^66^. The output of this step is a read count table of transposon insertions (including the orientation) at every TA dinucleotide site in the genome. The insertion density (TAs_hit/TA_sites) as reported by TPP is provided in Supplementary Data 4–6. For downstream analyses, TA sites with ten or fewer mapped reads were set to zero to reduce noise from spurious reads.

### Gene essentiality analysis

To account for the less complex structure and smaller size of phage genomes compared with bacterial genomes^26,65^, we developed a custom gene essentiality classification pipeline in RStudio (v4.3.2). This approach integrates insertion site saturation, positional bias and read counts to more accurately capture the range of fitness effects observed in our data, which did not conform solely to binary essentiality classifications. Insertion orientation was not included as a parameter in gene essentiality calling because after outgrowth, insertions were almost exclusively aligned with gene directionality (Supplementary Fig. 5). For each gene, we calculated saturation as the proportion of TA sites with non-zero read counts. Genes with saturation below 0.2 were classified as essential, while those with saturation above 0.8 were classified as non-essential. Genes falling between these thresholds were further evaluated in two steps. We first assessed the positional distribution of insertions. Genes with a strong 3′-end insertion bias or with insertions restricted to one half of the gene, indicative of an essential region within a gene, were also classified as essential. As the transposon carries stop codons in all three reading frames, this asymmetric insertion pattern can be observed when the 3′ portion of a gene is dispensable, but insertions would not be recovered in the opposite case because disruption of the 5′ region of the gene prevents production of the downstream part of the protein. We then used read count as a proxy for the fitness cost of gene disruptions. Genes with saturation between 0.2 and 0.4 were classified as ‘strong fitness defect’ if more than 50% of insertions had read counts below the dataset median, indicating that gene disruption imposes a high fitness cost. By contrast, genes with saturation between 0.7 and 0.8 were classified as ‘reduced fitness’ if the majority of insertions had read counts above the median, indicating a non-essential gene whose disruption has a mild fitness cost. Genes with saturation between 0.4 and 0.7, or those that did not meet the above criteria, were classified as ‘intermediate’, reflecting phenotypes that were neither clearly deleterious nor fully tolerated. Each gene was thus ultimately assigned to one of five HIDEN-SEQ categories: essential, strong fitness defect, intermediate, reduced fitness or non-essential. Genes containing 5 or fewer TA sites were omitted from classification owing to insufficient insertion sites to support confidence calls. To account for stochastic variation introduced by subsampling, we generated five replicate datasets by randomly sampling from the same set of preprocessed reads at fixed sequencing depth. Each replicate was processed independently through the full analysis pipeline. Final gene essentiality calls were assigned on the basis of consistent classification in at least three out of five replicates. Insertions at TA sites outside of coding sequences were recorded and analysed but not subjected to an essentiality analysis.

### Conditional essentiality analysis

To identify genes that become essential only under specific experimental conditions, we used the resampling method implemented in TRANSIT^65^, which determines read counts that are significantly different between two conditions using a non-parametric permutation test. To account for differences in sequencing depth between datasets, we used the subsampling at a fixed depth, as described above, and normalized the read counts using the trimmed total reads (TTR) method^65^. Instead of comparing each condition to the original reference library, we used parallel control libraries that were grown under the same conditions but lacking selective pressure. As an example, infection of a host carrying an empty vector was used as a control to infections of the same host carrying a plasmid expressing an antiphage defence system. This approach minimized noise from potential random loss of mutants during library passaging and helped distinguish true conditionally essential genes from those that get depleted after prolonged selection (Supplementary Discussion B). The resampling test was run with 10,000 permutations (-s 10000), and returned, for each gene, a *P* value and the corresponding log_2_FC in normalized insertion counts between conditions. The *P* values were adjusted for multiple testing by the Benjamini–Hochberg procedure to control the false-discovery rate at <0.05 (*q* value). Given the small size of the phage genomes, transposon insertion profiles were also inspected manually as a complementary check. For the CBASS condition, resampling results were not considered owing to random mutant losses on this sample (because of toxicity of defence genes); however, manual inspection revealed one gene with clear gene-wide depletion. In the Bas54 HIDEN-SEQ library grown in *E. coli* CFE303, the *bas54_0108* gene showed clear depletion relative to the reference strain but was not detected by the standard procedure owing to high insertion counts at the 3′ end. To account for this, we applied trimming of TA sites in the C terminus (-iC 20) during resampling.

### Genome sequencing and assembly of new uropathogenic *E. coli* strains

For the whole-genome sequencing of newly isolated uropathogenic *E. coli* strains, DNA extraction was performed using the Quick-DNA Fungal/Bacterial microprep kit (Zymo research, cat. no. D6007) according to the manufacturer’s instructions. Short-read whole-genome sequencing was performed at SeqCenter (https://www.seqcenter.com/) using Illumina technology and parallel long-read sequencing was carried out in-house using an Oxford Nanopore Technologies (ONT) platform. Read quality was first assessed using FastQC (v.0.11.8)^62^ and subsequently quality-trimmed with Trimmomatic (v.0.39)^67^ to remove low-quality bases from the first 15 positions of each read. Hybrid de novo genome assemblies combining Illumina short reads and ONT long reads were generated using Unicycler (v0.5.1)^68^. Assemblies were generated in normal, bold and conservative modes, and the assembly with the highest contiguity and completeness for each isolate was selected for downstream analyses.

### Genome sequencing and assembly of individual phage mutants

Whole-genome sequencing of individual phage mutants was performed using commercial DNA sequencing as described previously^41,69^. In brief, phage genomic DNA was isolated from the lysate using the Norgen Biotek Phage DNA Isolation Kit and sequenced at SeqCenter (https://www.seqcenter.com/) with Illumina technology. For selected Bas54-knockout mutants generated with CRISPR–Cas9 sequencing was performed at SeqVision (https://seqvision.com/) using Oxford Nanopore technology. Genome assembly was done using Unicycler (v0.4.8)^68^, and the downstream analyses were performed in Geneious Prime 2023.0.4.

### Structure predictions and analysis of newly identified antidefence genes

To generate hypotheses about the function of the candidate antidefence phage genes, we searched for structural homologues in the Protein Data Bank using HHpred^70^ and Dali server^71^. Structure predictions of phage proteins were performed using AlphaFold 2 on the ColabFold webserver^72^ with default parameters. The structural visualizations were generated using PyMOL Molecular Graphics System (Version 3.0.4, Schrodinger).

### Reporting summary

Further information on research design is available in the Nature Portfolio Reporting Summary linked to this article.

## Supplementary information

Supplementary Information Supplementary Discussion A and B, Figs. 1–6, Tables 1 and 2 and source data for supplementary figures.

Reporting Summary

Peer Review File

Supplementary Data 1 Phages used in this study and annotation tables.

Supplementary Data 2 Prediction of defense proteins encoded by *E. coli* uropathogenic isolates (using DefenseFinder).

Supplementary Data 3 Construction of all plasmids generated in this study.

Supplementary Data 4 Processed read counts, gene essentiality classification and conditional essentiality for phage T4.

Supplementary Data 5 Processed read counts, gene essentiality classification and conditional essentiality for phage Bas37.

Supplementary Data 6 Processed read counts, gene essentiality classification and conditional essentiality for phage Bas54.

## Source data

Source Data Extended Data Fig. 5 Unprocessed agarose gels.

## Data Availability

All data supporting the findings of this study are available in this Article. Data from the HIDEN-SEQ experiments are organized by phage in Supplementary Data 4–6. The raw reads of our HIDEN-SEQ experiments have been deposited in the NCBI Sequence Read Archive (SRA) under BioProject accession no. PRJNA1454991. The corresponding SRA run accessions (SRR) are listed in Supplementary Data 4–6. In addition, the raw sequencing data and genome assemblies for the uropathogenic *E. coli* isolates are available in the European Nucleotide Archive (ENA) under study accession no. PRJEB111350. HIDEN-SEQ plasmids are available via Addgene (https://www.addgene.org/browse/article/28275208/) with IDs 257247 (pAH186ColE1_Himar1_T’ase), 257248 (pAH160_Tn_AIcrVIA3_v2), 257249 (pDH160_Tn_AcrVIA1_v2), 257250 (pAH221_LbuCas13a), 257251 (pAH218_LbuCas13a_e), 257252 (pAH221_LseCas13a) and 257253 (pAH218_LseCas13a_e). Source data are provided with this paper.

## Code availability

The Rstudio code used for the gene essentiality analysis of HIDEN-SEQ libraries is available via Zenodo at https://doi.org/10.5281/zenodo.20179451 (ref. ^73^).
