# Supplementary material for: Systematic mapping of bacteriophage gene essentiality with HIDEN-SEQ
Source: Nat Microbiol. 2026 Sep 25;11(10):2751–63. doi: 10.1038/s41564-026-02455-8 (PMC13716836; doi:10.1038/s41564-026-02455-8)

Unprocessed agarose gels

Extended Data Fig. 5a

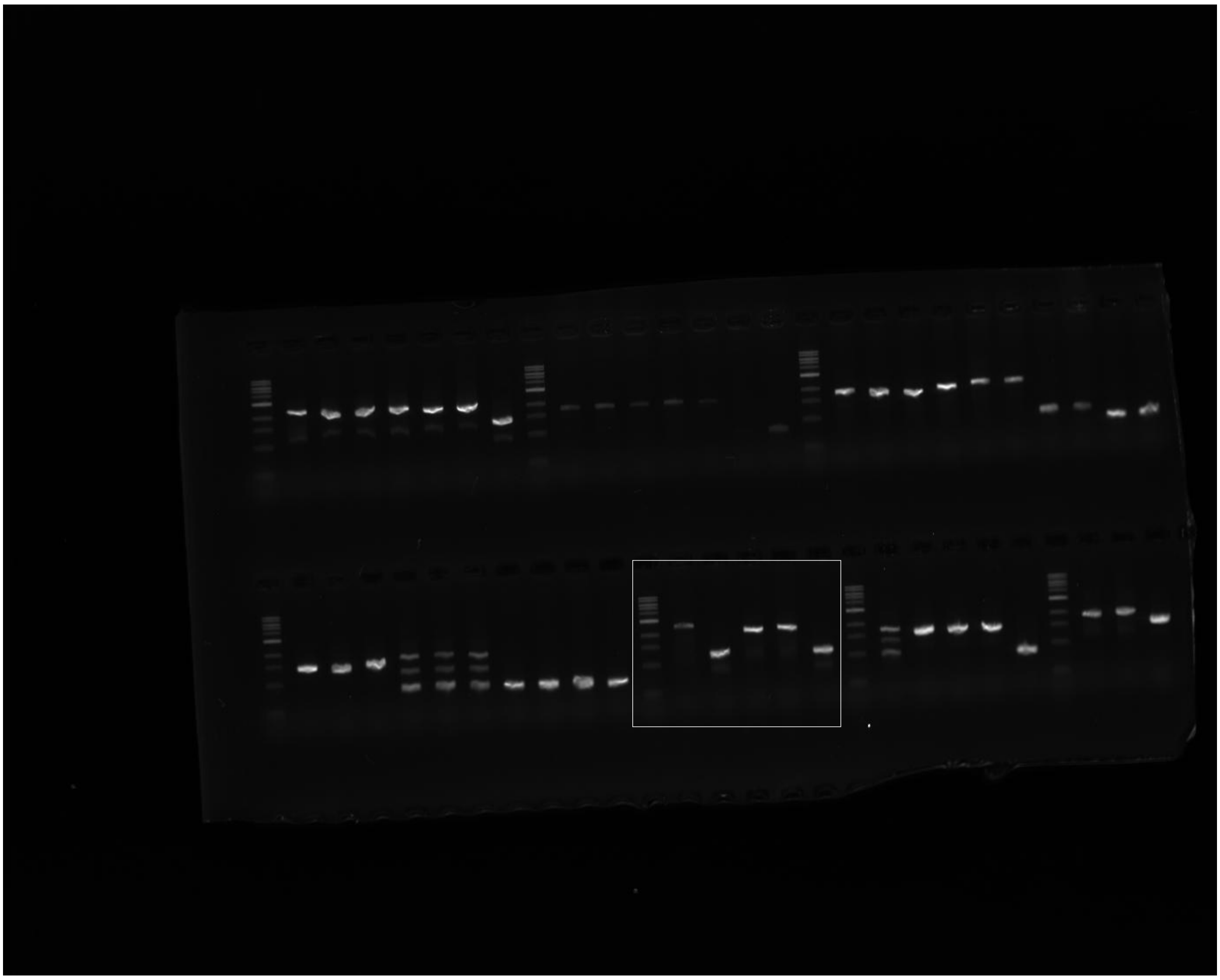

Extended Data Fig. 5b

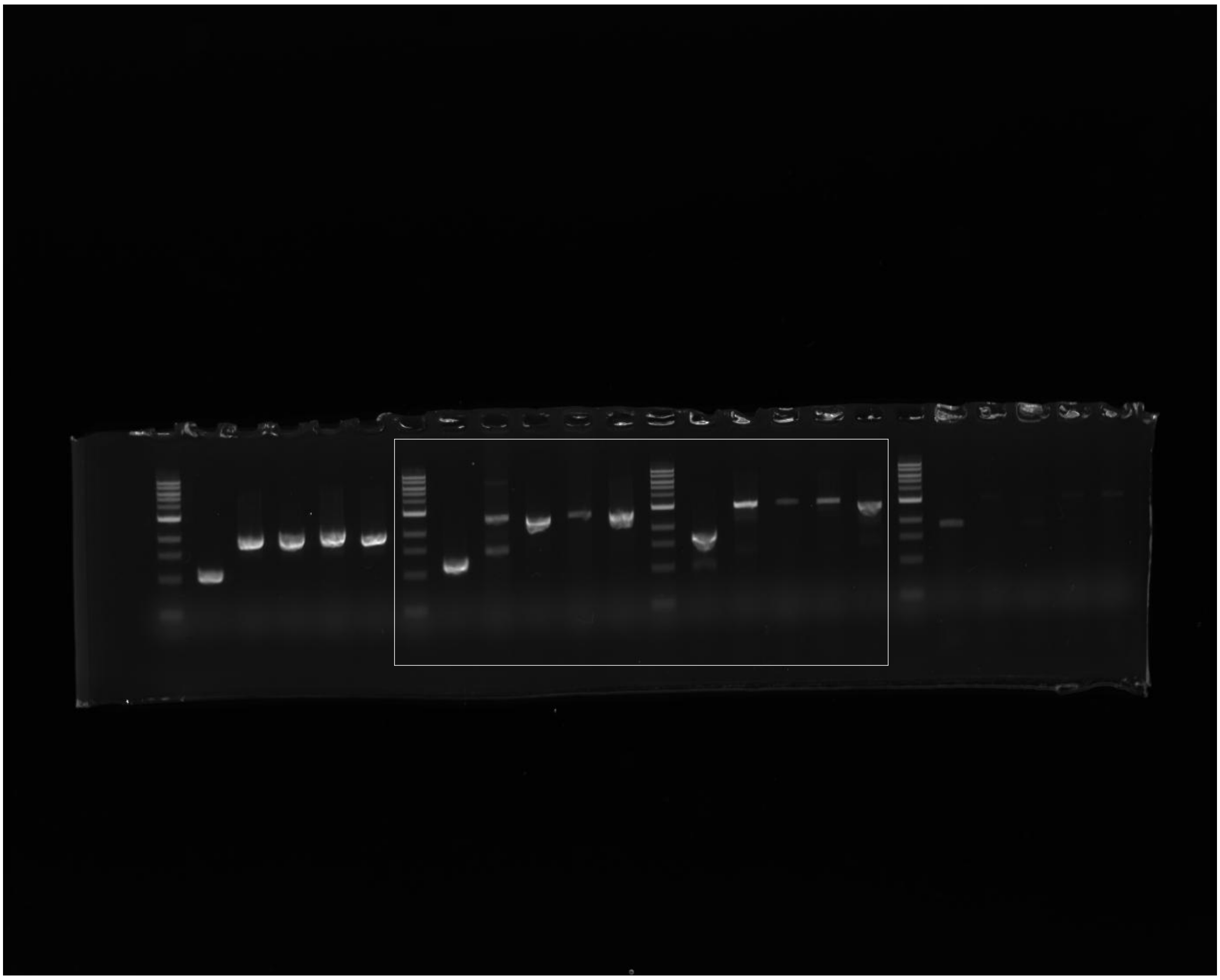

Supplement: Supplementary file 1 [file 41564_2026_2455_MOESM10_ESM.pdf]
