# Supplementary material for: Systematic mapping of bacteriophage gene essentiality with HIDEN-SEQ
Source: Nat Microbiol. 2026 Sep 25;11(10):2751–63. doi: 10.1038/s41564-026-02455-8 (PMC13716836; doi:10.1038/s41564-026-02455-8)

nature microbiology

Supplementary information https://doi.org/10.1038/s41564-026-02455-8

Systematic mapping of bacteriophage gene essentiality with HIDEN-SEQ

In the format provided by the authors and unedited

Table of Contents

Supplementary Discussion

Supplementary Figures

Supplementary Tables

Supplementary References

Source Data Supplementary Figures

Supplementary Discussion

A. Discrepancies to previous gene essentiality analyses for phage T4 and polar effects

Despite large agreement with previous literature, a few genes of model phage T4 (*inh, hoc, segE, uvsW, segC,* and *wac*) were scored as essential by HIDEN-SEQ while previous work using other methods had indicated that they may be not essential under standard laboratory conditions (Fig. 1b)[1,2]. One possible explanation for these few observed differences to previous work may be the different strategies used to study gene essentiality. For example, the severe depletion of transposon insertions disrupting the *wac* gene locus – encoding the phage whiskers – cannot be directly compared to earlier work that had observed no strong essentiality of the Wac protein based on conditional translational inhibition by amber mutants[3], an approach that may still allow residual protein expression. Consistently, also our attempts to generate a targeted knockout of *wac* were unsuccessful even in the presence of gene complementation *in trans* (Supplementary Fig. 4). These results highlight an inherent limitation of all transposon screens which interrogate fitness effects related to the integrity of a specific DNA locus and not directly the presence or absence of certain gene products. While the Wac protein may not be required for growth of phage T4, our results clearly show that the *wac* locus is essential. We anticipate that similar effects affecting our study or previous work are causal to the few observed discrepances in reported T4 gene essentiality, and they may also affect TnSeq with other phages.

A known caveat of TnSeq screens is that transposon insertions can cause polar effects by disturbing the expression of downstream genes which may distort gene essentiality calls, especially in case of insertions at non-essential loci which affect downstream essential genes[4,5]. Importantly, the large agreement of our HIDEN-SEQ results for phage T4 with previous work suggests that polar effects do not greatly affect the results obtained with our technology. To probe how polar effects would appear in HIDEN-SEQ, we deliberately provoked the disruption of transcriptional units by supplementing our transposon with a transcriptional terminator for a dedicated set of experiments. Conversely, our T4 reference HIDEN-SEQ library had been generated with the same transposon lacking the terminator. Interestingly, the library with terminator showed depletion of insertions upstream of several essential genes, a pattern not observed in our reference library (Extended Data Fig. 2a). Consistent with these observations, targeted insertions at multiple loci

(*alc, dsbA, 47.1* and upstream of *42*) recovered substantially fewer mutants when the *acrVIA1* selection marker was introduced with a transcriptional terminator than without it (Extended Data Fig. 2b). These results suggest that polar effects do not greatly distort the HIDEN-SEQ data generated with our transposon that lacks a transcriptional terminator at least for phage T4. However, it is clear that polar effects can nonetheless influence fitness effects of insertions and gene essentiality calls in other phages. It is therefore important to validate key results with orthogonal approaches such as clean knockouts, transcriptional knockdowns, or conditional translational inhibition through amber codons.

Besides polar effects on essential genes adjacent to the insertion site, other locus-dependent effects such as differences in expression of the anti-CRISPR selection marker could affect results obtained with HIDEN-SEQ and may explain some of the few observed discrepancies to previous work. However, the large agreement of our T4 data with previous work and the absence of obvious biases, e.g, between insertions in early and late genes, do not suggest that these phenomena would have significant effects on HIDEN-SEQ results for the tested phages. This is further supported by the recovery of disruptive insertions in several *bona fide* Bas54 tail fiber genes, which are likely expressed late during infection (Fig. 3b).

In our results and in a parallel study on phages infecting *Prodigiosinella*[6], insertions were originally observed evenly in both transposon orientations but after outgrowth almost completely aligned with gene directionality (Supplementary Fig. 5a, b). While this observation does not affect library density or the results of HIDEN-SEQ, it highlights the impact of local transcription and transcriptional conflicts on viral fitness across diverse phages.

B. Phage anti-defense factors against Mokosh type I and regrowth-associated fitness effects

The ADP-ribosyltransferases and glucosyltransferases of T-even phages were identified as anti-defense factors against Mokosh type I system of *E. coli* CFE331 strain. Notably, the glucosyltransferases did not appear as conditionally essential when the T4 and Bas37 HIDEN-SEQ libraries were grown in *E. coli* CFE331 (Fig. 4a, b). This may indicate insufficient expression in the native host, inhibition by prophage-encoded anti-defense factors present in CFE331 or a synergistic interaction with a defense system specific to *E. coli* K-12. Comparison between two

closely related phages, T4 and Bas37, highlights overlapping mechanisms yet distinct gene requirements in their counter-defense against Mokosh (Fig. 4e). In Bas37, *alt* (*bas37_0033*), *βα-gt* (*bas37_0154*), and *modA* (*bas37_0131*), which is another known ADP-ribosyltransferase[7], were identified as conditionally essential genes. The requirements of Bas37 for both *modA* and *alt* suggest functional compensation for the weaker activity of Bas37 Alt (Fig. 4e and Extended Data Fig. 9b). In contrast, in T4 phage, complete loss of insertions in the presence of Mokosh type I was observed only for T4 *alt* and *α-gt* gene, while there was only a minor reduction in a few other genes, such as *modA*, *alc* and *β-gt*. These results also point to differences in glycosylation of hydroxymethyl cytosine residues, as previously reported for T-even relatives[8], which may also account for the higher sensitivity of Bas37 to Mokosh type I (Fig. 4d). Notably, the Bas37 *βα-gt* is orthologous to the T6 *βα-gt* gene, which adds a second glucosyl group to generate gentiobiosyl-hydroxymethyl cytosine[8]. However, the Bas37 *α-gt* gene did not appear conditionally essential in this case because its depletion was due to a regrowth-associated fitness effect rather than only selective pressure. Such effects were also observed for a few genes in other experiments where insertions tolerated in the reference library were progressively depleted upon passaging. Consequently, such genes may not be identified as conditionally essential. Similar effects have also been reported in bacterial TnSeq[9], where the measured fitness of insertions can shift across serial passages, indicating a gradual fitness disadvantage that is only revealed under longer selection. Such progressive changes can occur both broadly (Supplementary Fig. 6a, b) but also in specific phage-host settings (Supplementary Fig. 6c).

Supplementary Figures

Supplementary Fig. 1 | The CRISPR-Cas13a / anti-CRISPR based selection of transposition events into T4 phage genome.

Supplementary Fig. 2 | Genome-wide essentiality mapping of Bas37 phage.

Supplementary Fig. 3 | Orthogonal validation of Bas54 HIDEN-SEQ essentiality calls.

Supplementary Fig. 4 | Attempts to generate T4 *wac* deletion mutants by targeted phage engineering.

Supplementary Fig. 5 | Orientation bias of transposon insertions after outgrowth in HIDEN-SEQ libraries.

Supplementary Fig. 6 | Gene fitness effects upon passaging of the transposon phage libraries.

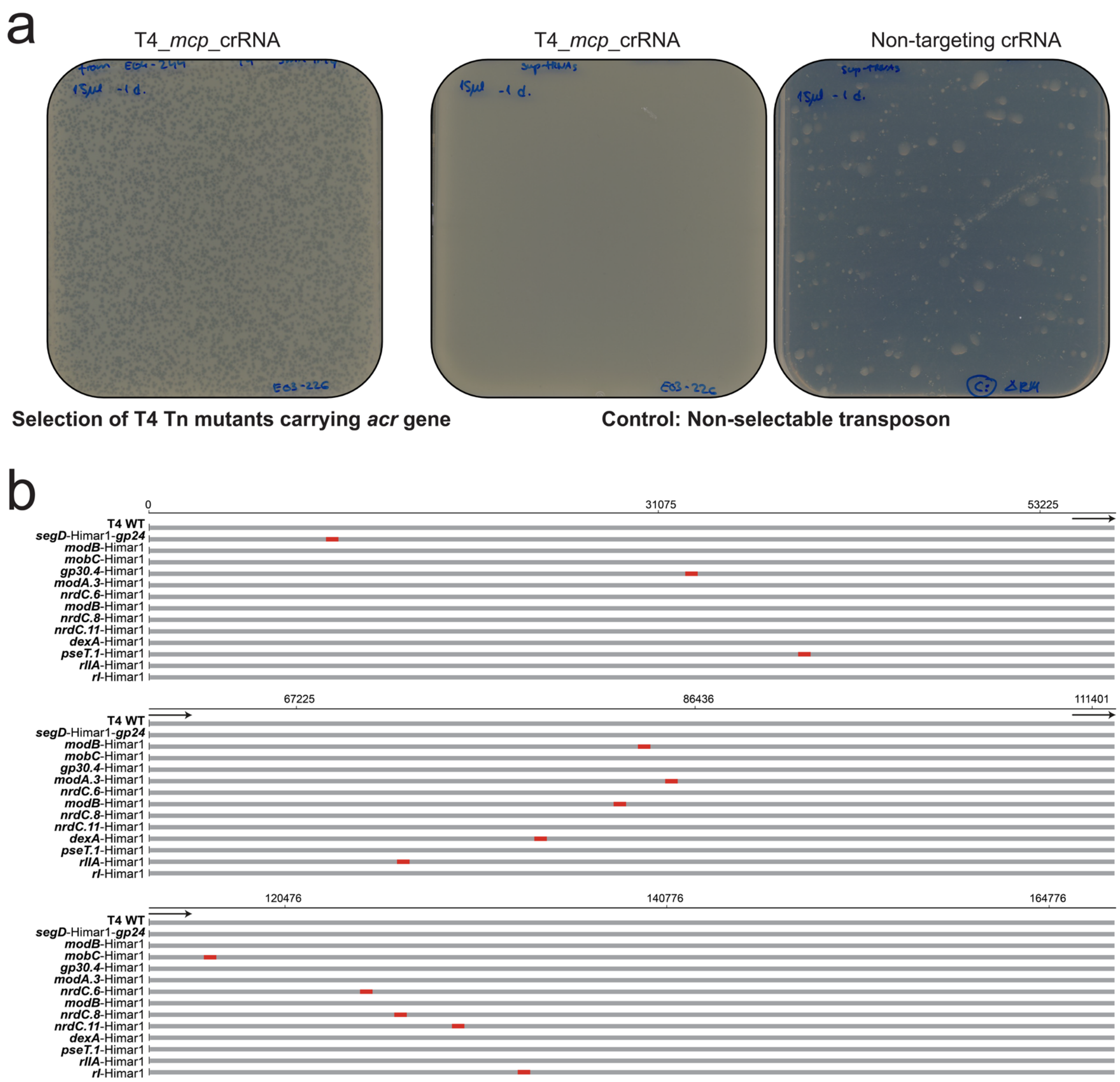

Supplementary Fig. 1 | The CRISPR-Cas13a / anti-CRISPR based selection of transposition events into T4 phage genome.

a, T4 pool of anti-CRISPR transposon mutants plated on *E. coli* K-12 expressing *cas13a* with the crRNA targeting the transcripts of the major capsid protein. The plaques indicate phage growth enabled by anti-CRISPR activity (left). A T4 mutant pool generated with a non-selectable transposon (lacking anti-CRISPR) plated on the same host does not show plaque growth in presence of CRISPR-Cas13a selection (middle plate) but results in full lysis (right) without selection. **b**, Whole-genome alignment of wildtype T4 phage with 13 individual clones that were randomly picked after CRISPR-Cas13a selection and then sequenced. Red marks indicate transposon insertions identified in each clone. Alignments were generated using MAFFT v7.490 implemented in Geneious Prime 2023.0.4 with default settings.

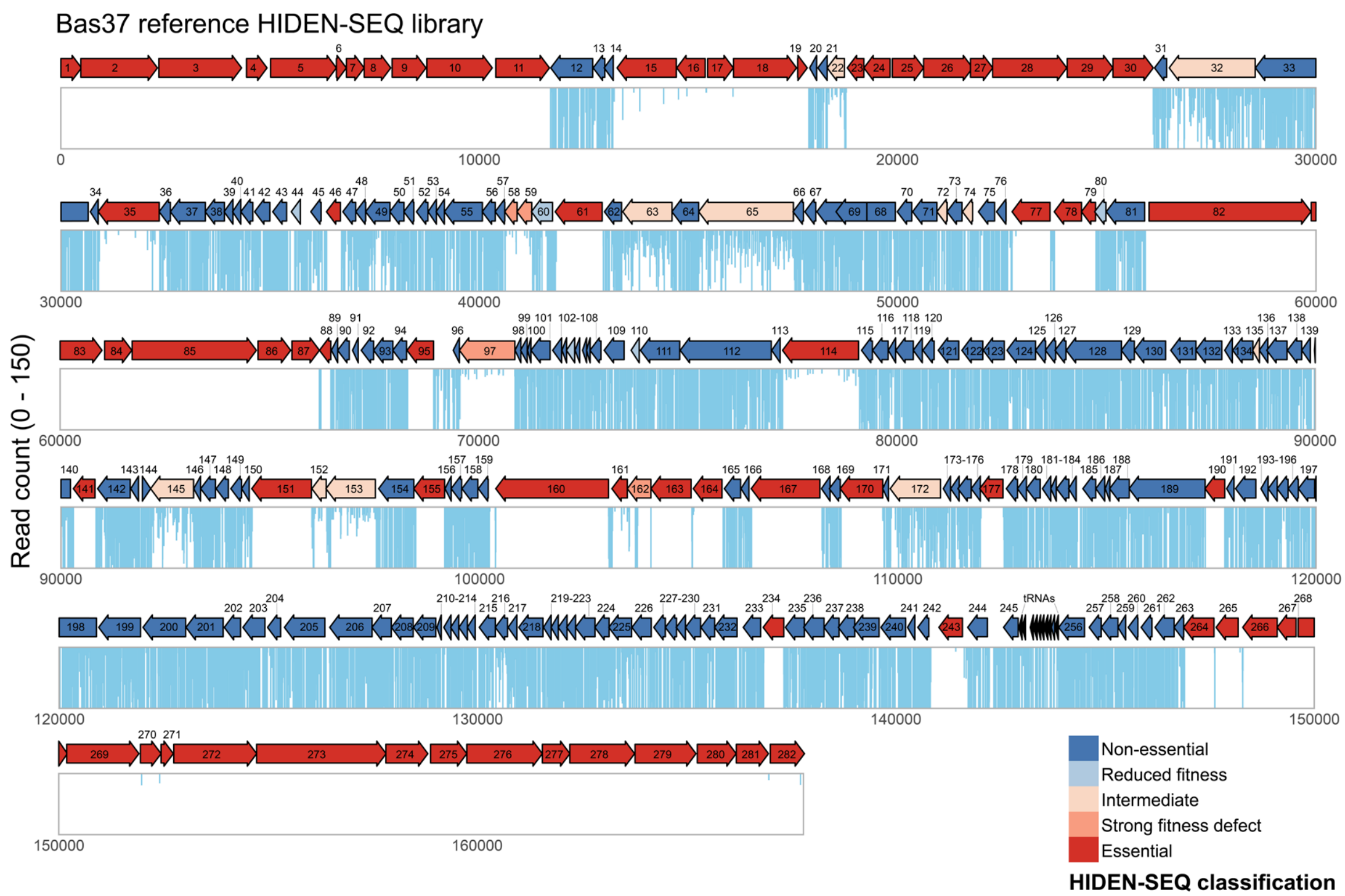

Supplementary Fig. 2 | Genome-wide essentiality mapping of Bas37 phage.

Phage Bas37 HIDEN-SEQ gene essentiality map with position 1 set to the start of the small terminase subunit gene. Arrows present the coding sequences with the orientation indicating the direction of transcription. Arrow colours correspond to HIDEN-SEQ classification categories derived from transposon insertion profiles: non-essential (dark blue), reduced fitness (light blue), intermediate (light orange), strong fitness defect (orange), and essential (red). Vertical bars represent the number of transposon insertions at TA dinucleotide sites with heights indicating total read counts (shown up to a maximum of 150). Genes containing ≤5 TA sites are shown in black and were not classified. Source data: Supplementary Data 5 (Data S3-S4).

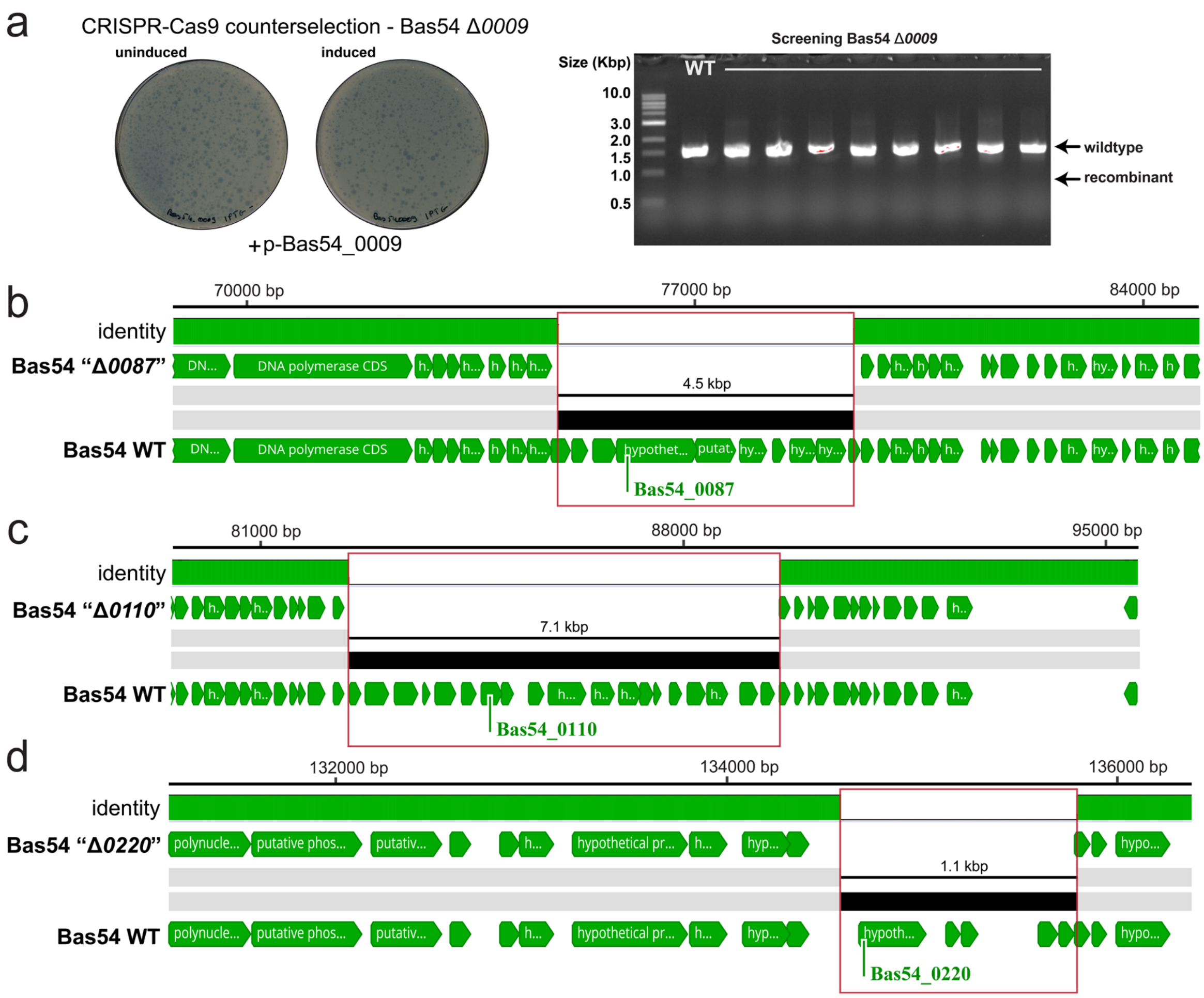

Supplementary Fig. 3 | Orthogonal validation of Bas54 HIDEN-SEQ essentiality calls.

a, Whole-plate infections on the Cas9 selection strain during attempts to delete *bas54_0009* (major capsid protein) with or without induction of the phage gene from the complementation plasmid. No increase in the number of plaques is observed upon induction, as would be expected if complementation enabled recovery of deletion mutants. PCR screening of several plaques confirms the absence of recombinant phages indicating that recovered mutants are Cas9 escapees. Deletion of *bas54_0009* could not be obtained under these conditions which highlight the limitations of genetic complementation for knocking out essential phage genes. **b**, **c**, **d**, The illustration shows sequence alignments at the targeted loci in Bas54 "Δ*0087*" (**b**), "Δ*0110*" (**c**), "Δ*0220*" (**d**) mutants compared to the wildtype phage, highlighting the deleted regions. Alignments were generated using MAFFT v7.490 implemented in Geneious Prime 2023.0.4 with default settings. In all cases, only mutants carrying larger deletions could be obtained. The additional genes that are removed in these mutants are classified as not essential by HIDEN-SEQ (Fig. 3b). Consistent with this, plaque formation of all three mutants is restored when only the corresponding targeted gene is provided *in trans* (see Extended Data Fig. 6e).

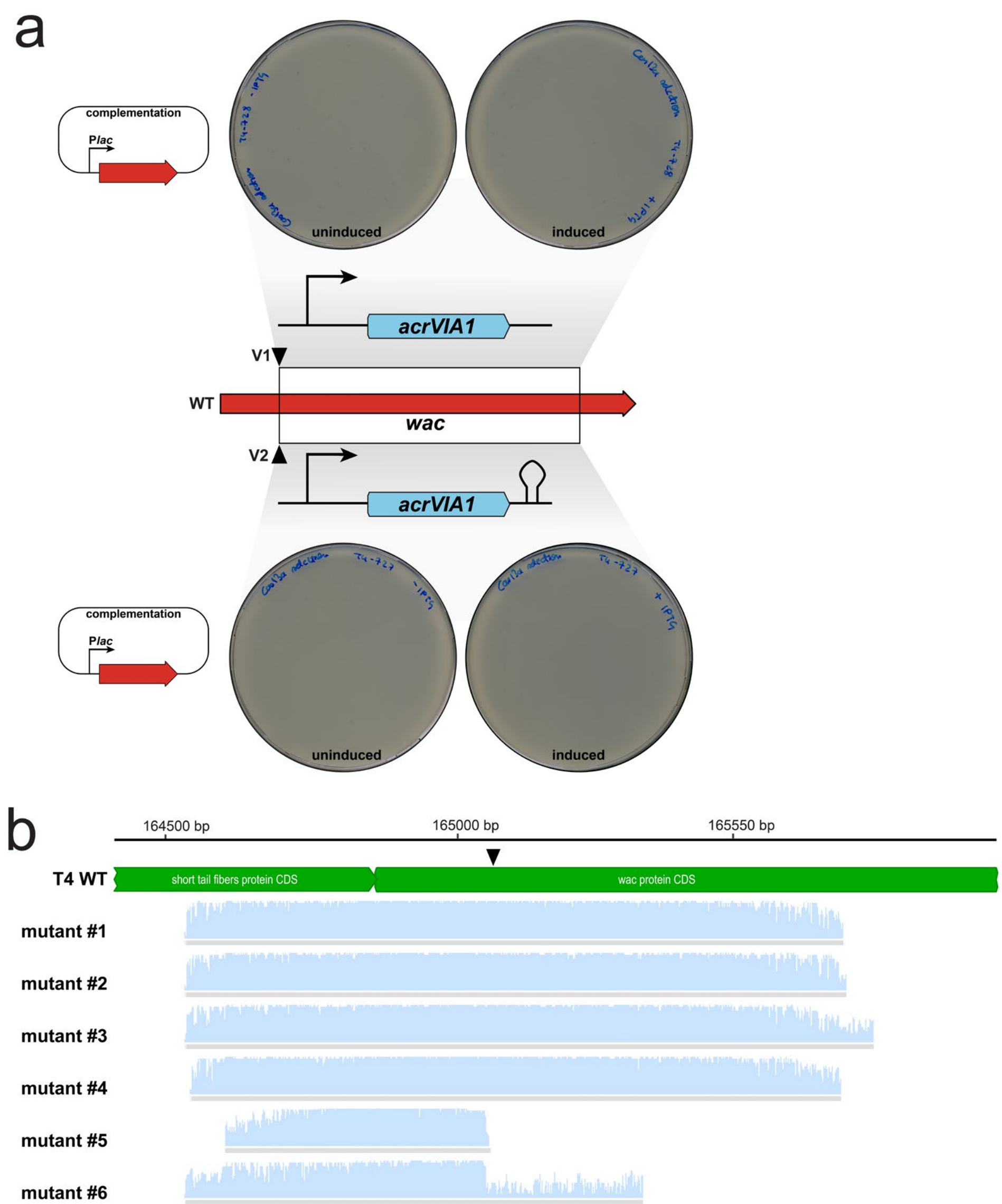

Supplementary Fig. 4 | Attempts to generate T4 *wac* deletion mutants by targeted phage engineering.

a, Whole-plate infections on Cas13a selection strain during attempts to replace the indicated region of *wac* (boxed) of phage T4 with *acrVIA1* in the absence (V1) or presence (V2) of a transcriptional terminator, with or without induction of *wac* expression from a plasmid. In all cases, only very few plaques were obtained, which is consistent with a strong fitness cost associated with disruption of this locus (see also panel b). **b**, Read mapping of PCR products from representative cases. Across multiple independent screening attempts, no recombinant phages could be identified. Instead, Sanger sequencing reads were ambiguous, mapped to wildtype phage (mutants #1-4), or broke off near the intended start of the deletion (mutants #5-6), suggesting possible sequence rearrangements.

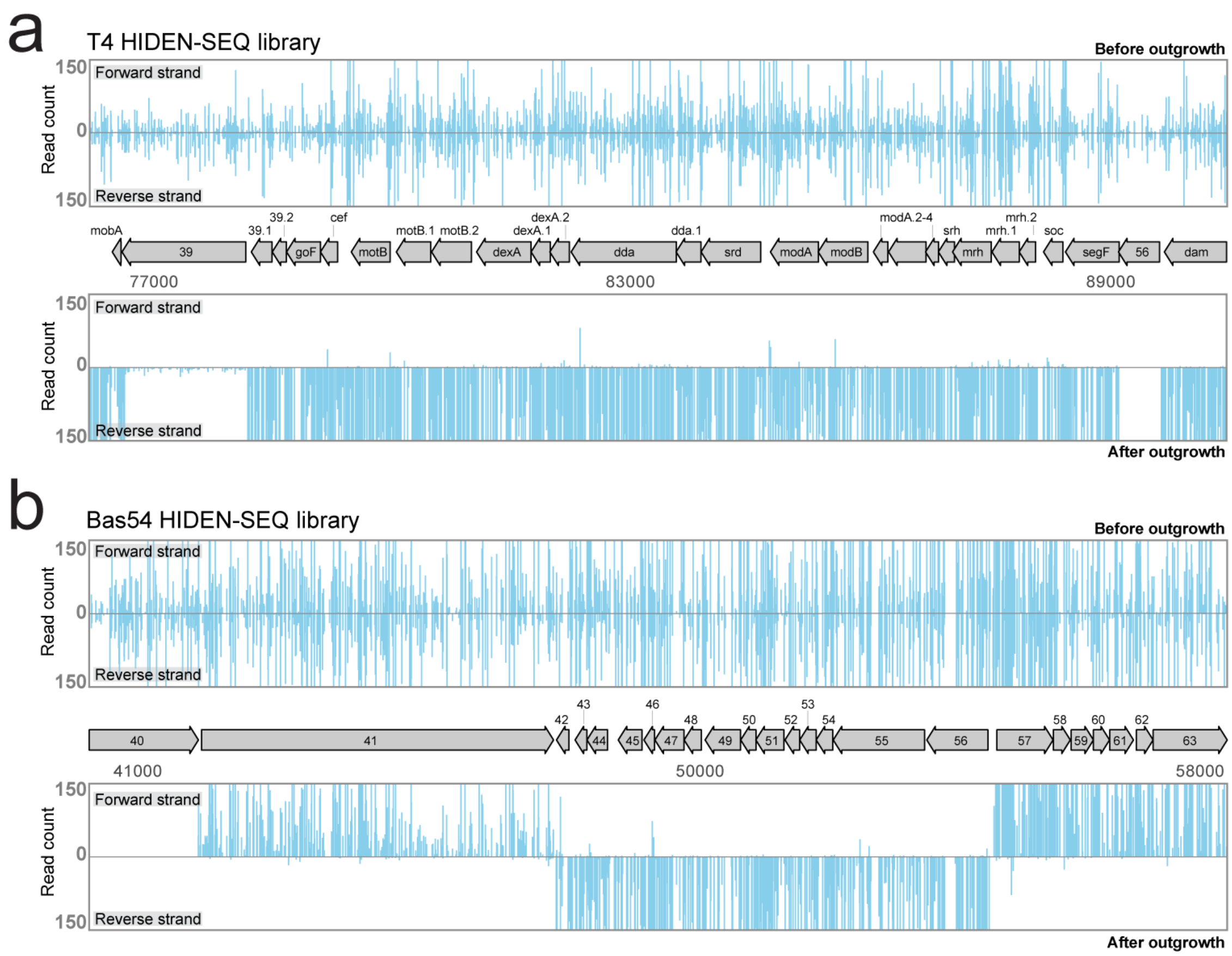

Supplementary Fig. 5 | Orientation bias of transposon insertions after outgrowth in HIDEN-SEQ libraries.

a, **b**, Transposon insertion profiles across a representative region on the T4 (**a**) and Bas54 (**b**) genome before (top track) and after (bottom track) outgrowth. Vertical bars represent the number of transposon insertions at TA dinucleotide sites with heights indicating read counts (shown up to a maximum of 150), plotted separately for forward and reverse strand. Arrows indicate coding sequences with orientation showing the direction of transcription. Prior to outgrowth, insertions are distributed in both orientations. After outgrowth, insertions are strongly biased in the direction of gene transcription. Source data: Supplementary Data 4 (Data S2-S3, panel a) and Supplementary Data 6 (Data S2-S3, panel b).

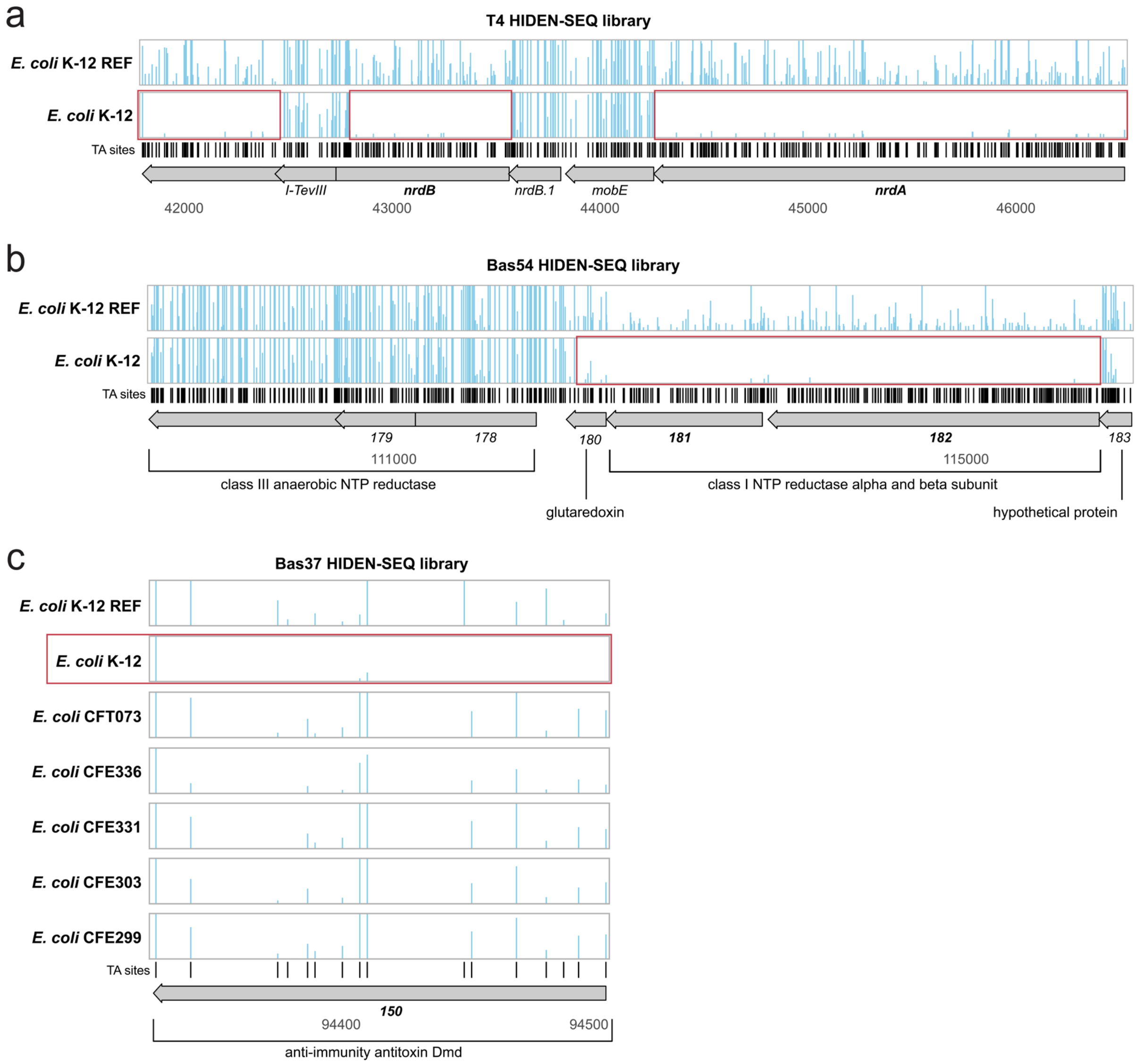

Supplementary Fig. 6 | Gene fitness effects upon passaging of the transposon phage libraries.

a, Transposon insertion profiles at the locus encoding ribonucleoside reductase subunits (*nrdA* and *nrdB)* genes of phage T4 for the T4 reference HIDEN-SEQ library (top track) and those obtained upon passaging of the library in *E. coli* K-12 BW25113 host (bottom track). Vertical bars represent the number of transposon insertions at TA sites (shown with black tick marks) with heights indicating read counts (shown up to a maximum of 150). The illustration highlights that the observed depletion of transposon insertion mutants is specific to *nrdA* and *nrdB* genes. This depletion was also observed in other hosts (Supplementary Data 4). Source data: Supplementary Data 4 (Data S3, Data S17). **b**, Same as in (a), but for ribonucleotide reductase subunits in Bas54 phage. Source data: Supplementary Data 6 (Data S3, S5). **c**, Transposon insertion profiles at the locus encoding the Dmd antitoxin (*bas37_0150*) gene ortholog of Bas37 phage in the Bas37 reference HIDEN-SEQ library (top track) and those obtained upon passaging

of the library in the *E. coli* K-12 BW25113 host expressing *rnlAB* or different *E. coli* clinical isolates. The illustration highlights that the insertion depletion is host-specific because *bas37_0150* insertions are progressively depleted (more essential) upon passaging in *E. coli* K-12, but not in the other *E. coli* strains. Source data: Supplementary Data 5 (Data S3, S5-S10).

Supplementary Tables

Supplementary Table 1. List of all bacterial strains used in this study

Identifier	Genotype	relevant plasmid(s)	Selection	Source/Description
AH-E03-200	*E. coli* K-12 MG1655 ΔRM MG1655 Δ*mrr-hsdRMS-mcrBC* Δ*mcrA*	none	none	Our laboratory collection[10]; strain lacking all known restriction systems of *E. coli* K-12
AH-E01-047	*E. coli* K-12 BW25113 F^- Δ(*araD-araB*)567, Δ*lacZ4787(::rrnB-3)*, λ^-, *rph-1*, Δ(*rhaD-rhaB*)568, *hsdR514*	none	none	Our laboratory collection[11]
DH-E01-007	*E. coli* K-12 MG1655 ΔRM MG1655 Δ*mrr-hsdRMS-mcrBC* Δ*mcrA*	pAH221_LbuCas13a	Cam25	Our laboratory collection; strain carrying the LbuCas13a expression plasmid
AH-E10-748	*E. coli* K-12 DH5α *(?) F- endA1 glnV44 thi-1 recA1 relA1 gyrA96 deoR nupG Φ80dlacZΔM15 Δ(lacZYA-argF)U169, hsdR17(rK- mK+), λpir*	pAH218_LbuCas13a_e	Gen20	Our laboratory collection; strain carrying crRNA cassette plasmid for cloning LbuCas13a spacers
DH-E01-008	*E. coli* K-12 MG1655 ΔRM MG1655 Δ*mrr-hsdRMS-mcrBC* Δ*mcrA*	pAH221_LbuCas13a, pAH218_LbuCas13a_e	Cam25, Gen20	Our laboratory collection; strain carrying the LbuCas13a expression plasmid and the empty crRNA cassette plasmid
DH-E03-201	*E. coli* K-12 MG1655 ΔRM MG1655 Δ*mrr-hsdRMS-mcrBC* Δ*mcrA*	pAH221_LseCas13a	Cam25	This study; strain carrying the LseCas13a expression plasmid
DH-E03-182	*E. coli* K-12 DH5α *(?) F- endA1 glnV44 thi-1 recA1 relA1 gyrA96 deoR nupG Φ80dlacZΔM15 Δ(lacZYA-argF)U169, hsdR17(rK- mK+), λpir*	pAH218_LseCas13a_e	Gen20	This study; strain carrying crRNA cassette plasmid for cloning LseCas13a spacers
DH-E03-173	*E. coli* K-12 MG1655 ΔRM MG1655 Δ*mrr-hsdRMS-mcrBC* Δ*mcrA*	pAH221_LseCas13a, pAH218_LseCas13a_e	Cam25, Gen20	This study; strain carrying the LseCas13a expression plasmid and the empty crRNA cassette plasmid
DH-E02-160	*E. coli* K-12 MG1655 ΔRM MG1655 Δ*mrr-hsdRMS-mcrBC* Δ*mcrA*	pAH221_LbuCas13a, pAH218_LbuCas13a_Bas54_DNA_pol_H5	Cam25, Gen20	This study; LbuCas13a selection strain with crRNA targeting Bas54 phage
DH-E03-226	*E. coli* K-12 MG1655 ΔRM MG1655 Δ*mrr-hsdRMS-mcrBC* Δ*mcrA*	pAH221_LseCas13a, pAH218_LseCas13a_2_T4_mcp_CDS_2	Cam25, Gen20	This study; LseCas13a selection strain with crRNA targeting T4/Bas37 phage
DH-E05-359	*E. coli* EC100D K-12 *(?) F- mcrA Δ(mrr-hsdRMS-mcrBC) φ80dlacZΔM15 ΔlacX74 recA1 endA1 araD139 Δ(ara, leu)7697 galU galK λ- rpsL (StrR) nupG pir+(DHFR)*	pAH160_Tn_AIcrVIA3_v2	Kan50	This study; transposon plasmid with AIcrVIA3 as selection marker
DH-E04-279	*E. coli* EC100D K-12 *(?) F- mcrA Δ(mrr-hsdRMS-mcrBC) φ80dlacZΔM15 ΔlacX74 recA1 endA1 araD139 Δ(ara, leu)7697 galU galK λ- rpsL (StrR) nupG pir+(DHFR)*	pDH160_Tn_AcrVIA1_v2	Kan50	This study; transposon plasmid with AcrVIA1 as selection marker
DH-E05-365	*E. coli* EC100D K-12 *(?) F- mcrA Δ(mrr-hsdRMS-mcrBC) φ80dlacZΔM15 ΔlacX74 recA1 endA1 araD139 Δ(ara, leu)7697 galU galK λ- rpsL (StrR) nupG pir+(DHFR)*	pAH160_Tn_AcrVIA1_v3	Kan50	This study; transposon plasmid with AcrVIA1 as selection marker including a transcriptional terminator
DH-E05-364	*E. coli* K-12 BW25113 F^- Δ(*araD-araB*)567, Δ*lacZ4787(::rrnB-3)*, λ^-, *rph-1*, Δ(*rhaD-rhaB*)568, *hsdR514*	pAH186ColE1_Himar1_T'ase, pAH160_Tn_AIcrVIA3_v2	Amp50, Kan50	This study; transposition strain with transposon carrying AIcrVIA3
DH-E04-282	*E. coli* K-12 BW25113	pAH186ColE1_Himar1_T'ase,	Amp50, Kan50	This study; transposition strain with transposon carrying AcrVIA1

	F⁻ Δ(*araD-araB*)567, Δ*lacZ4787(::rrnB-3*), λ⁻, *rph-1*, Δ(*rhaD-rhaB*)568, *hsdR514*	pDH160_Tn_AcrVIA1_v2		
DH-E05-367	*E. coli* K-12 BW25113 F⁻ Δ(*araD-araB*)567, Δ*lacZ4787(::rrnB-3*), λ⁻, *rph-1*, Δ(*rhaD-rhaB*)568, *hsdR514*	pAH186ColE1_Himar1_T'ase, pAH160_Tn_AcrVIA1_v3	Amp50, Kan50	This study; transposition strain with transposon carrying AcrVIA1 including a transcriptional terminator
DH-E04-253	*E. coli* K-12 BW25113 F⁻ Δ(*araD-araB*)567, Δ*lacZ4787(::rrnB-3*), λ⁻, *rph-1*, Δ(*rhaD-rhaB*)568, *hsdR514*	pAH186ColE1_Himar1_T'ase, pAH160_Tn_sup-tRNAs_v2	Amp50, Kan50	This study; transposition strain with transposon carrying sup-tRNAs as non-selectable (with Cas13a) cassette
DH-E05-351	*E. coli* K-12 BW25113 F⁻ Δ(*araD-araB*)567, Δ*lacZ4787(::rrnB-3*), λ⁻, *rph-1*, Δ(*rhaD-rhaB*)568, *hsdR514*	pSH232_EcCdnG-WT	Amp50	Plasmid obtained from Prof. Philip J. Kranzusch; strain expressing CBASS EcCdnG operon[12]
DH-E05-352	*E. coli* K-12 BW25113 F⁻ Δ(*araD-araB*)567, Δ*lacZ4787(::rrnB-3*), λ⁻, *rph-1*, Δ(*rhaD-rhaB*)568, *hsdR514*	pSH233_EcCdnG-delHNH	Amp50	Plasmid obtained from Prof. Philip J. Kranzusch; strain expressing CBASS EcCdnG operon with inactive Cap effector proteins[12]
DH-E05-401	*E. coli* K-12 BW25113 F⁻ Δ(*araD-araB*)567, Δ*lacZ4787(::rrnB-3*), λ⁻, *rph-1*, Δ(*rhaD-rhaB*)568, *hsdR514*	pBR322_darTG1	Amp50	Plasmid obtained from Prof. Michele LeRoux; strain expressing DarTG1 system[13]
DH-E05-402	*E. coli* K-12 BW25113 F⁻ Δ(*araD-araB*)567, Δ*lacZ4787(::rrnB-3*), λ⁻, *rph-1*, Δ(*rhaD-rhaB*)568, *hsdR514*	pBR322_darTG2	Amp50	Plasmid obtained from Prof. Michele LeRoux; strain expressing DarTG2 system[13]
DH-E05-400	*E. coli* K-12 BW25113 F⁻ Δ(*araD-araB*)567, Δ*lacZ4787(::rrnB-3*), λ⁻, *rph-1*, Δ(*rhaD-rhaB*)568, *hsdR514*	pBR322_ev	Amp50	Plasmid obtained from Dr. Michele LeRoux; control strain for experiments with DarTG1 and DarTG2 carrying the empty vector[13]
DH-E05-338	*E. coli* K-12 BW25113 F⁻ Δ(*araD-araB*)567, Δ*lacZ4787(::rrnB-3*), λ⁻, *rph-1*, Δ(*rhaD-rhaB*)568, *hsdR514*	pUA139	Kan50	Our laboratory collection; control strain for experiments with RexAB carrying the empty vector
DH-E05-339	*E. coli* K-12 BW25113 F⁻ Δ(*araD-araB*)567, Δ*lacZ4787(::rrnB-3*), λ⁻, *rph-1*, Δ(*rhaD-rhaB*)568, *hsdR514*	pVD3_RexAB_v2	Kan50	This study; strain expressing RexAB
DH-E04-321	*E. coli* K-12 *ΔrnlA* BW25113 *rnlA::kanR(*pKD13*)*	none	Kan50	Keio collection mutant[14] obtained from Prof. Urs Jenal; strain not expressing RnlAB defense system
DH-E05-329	*E. coli* K-12 *ΔrnlA* BW25113 *rnlA::kanR(*pKD13*)*	pAH221_LseCas13a, pAH218_LseCas13a_2_empty	Kan50, Cam25, Gen20	Keio collection mutant not expressing RnlAB defense system carrying the LseCas13a expression plasmid and the empty crRNA cassette plasmid
DH-E05-330	*E. coli* K-12 *ΔrnlA* BW25113 *rnlA::kanR(*pKD13*)*	pAH221_LseCas13a, pAH218_LseCas13a_2_T4_crRNA_mcp_CDS_2	Kan50, Cam25, Gen20	Keio collection mutant not expressing RnlAB defense system LseCas13a selection strain with crRNA targeting T4 phage
DH-E05-336	*E. coli* K-12 *ΔrnlA* BW25113 *rnlA::FRT*	none	Kan50	Keio collection mutant[14] not expressing RnlAB defense system; Kanamycin cassette was FLPped out using pCP20
DH-E05-346	*E. coli* K-12 *ΔrnlA* BW25113 *rnlA::FRT*	pAH186ColE1_Himar1_T'ase, pDH160_Tn_AcrVIA1_v2	Kan50, Amp50	Keio collection mutant not expressing RnlAB defense system transposition strain with transposon carrying AcrVIA1
DH-E06-408	*E. coli* K-12 MG1655 ΔRM MG1655 Δ*mrr-hsdRMS-mcrBC* Δ*mcrA*	pAH210_T4_tk.4_AcrVIA1	Kan50	This study; strain carrying the homologous recombination plasmid for removing *tk.4*/*adfM* gene from T4 phage

DH-E05-396	*E. coli* K-12 MG1655 ΔRM MG1655 Δ*mrr-hsdRMS-mcrBC* Δ*mcrA*	pAH218_T4_vs_Acr VIA1	Gen20	This study; strain carrying the homologous recombination plasmid for removing *vs* gene from T4 phage
DH-E06-412	*E. coli* K-12 MG1655 ΔRM MG1655 Δ*mrr-hsdRMS-mcrBC* Δ*mcrA*	pAH210_T4_alt_Acr VIA1	Kan50	This study; strain carrying the homologous recombination plasmid for removing *alt* gene from T4 phage
DH-E06-413	*E. coli* K-12 MG1655 ΔRM MG1655 Δ*mrr-hsdRMS-mcrBC* Δ*mcrA*	pAH210_T4_cef_Acr VIA1	Kan50	This study; strain carrying the homologous recombination plasmid for removing *cef* gene from T4 phage
DH-E06-414	*E. coli* K-12 MG1655 ΔRM MG1655 Δ*mrr-hsdRMS-mcrBC* Δ*mcrA*	pAH210_T4_gp57B_ AcrVIA1	Kan50	This study; strain carrying the homologous recombination plasmid for removing *57B* gene from T4 phage
DH-E06-415	*E. coli* K-12 MG1655 ΔRM MG1655 Δ*mrr-hsdRMS-mcrBC* Δ*mcrA*	pAH210_T4_nrdC.1 _AcrVIA1	Kan50	This study; strain carrying the homologous recombination plasmid for removing *nrdC.1* gene from T4 phage
DH-E06-416	*E. coli* K-12 MG1655 ΔRM MG1655 Δ*mrr-hsdRMS-mcrBC* Δ*mcrA*	pAH210_T4_rnlA_A crVIA1_1	Kan50	This study; strain carrying the homologous recombination plasmid for inserting *acrVIA1* gene in T4 *rnlA* gene – position 1
DH-E06-417	*E. coli* K-12 MG1655 ΔRM MG1655 Δ*mrr-hsdRMS-mcrBC* Δ*mcrA*	pAH210_T4_rnlA_A crVIA1_2	Kan50	This study; strain carrying the homologous recombination plasmid for inserting *acrVIA1* gene in T4 *rnlA* gene – position 2
DH-E06-433	*E. coli* K-12 MG1655 ΔRM MG1655 Δ*mrr-hsdRMS-mcrBC* Δ*mcrA*	pAH210_T4_pseT_A crVIA1	Kan50	This study; strain carrying the homologous recombination plasmid for removing *pseT* gene from T4 phage
DH-E06-418	*E. coli* K-12 MG1655 ΔRM MG1655 Δ*mrr-hsdRMS-mcrBC* Δ*mcrA*	pAH210_Bas37_005 3_AcrVIA1	Kan50	This study; strain carrying the homologous recombination plasmid for removing *bas37_0053* gene from Bas37 phage
DH-E06-419	*E. coli* K-12 MG1655 ΔRM MG1655 Δ*mrr-hsdRMS-mcrBC* Δ*mcrA*	pAH210_Bas37_005 5_AcrVIA1	Kan50	This study; strain carrying the homologous recombination plasmid for removing *bas37_0055* gene from Bas37 phage
DH-E06-420	*E. coli* K-12 MG1655 ΔRM MG1655 Δ*mrr-hsdRMS-mcrBC* Δ*mcrA*	pAH210_Bas37_011 9_AcrVIA1	Kan50	This study; strain carrying the homologous recombination plasmid for removing *bas37_0119* gene from Bas37 phage
DH-E06-421	*E. coli* K-12 MG1655 ΔRM MG1655 Δ*mrr-hsdRMS-mcrBC* Δ*mcrA*	pAH210_Bas37_013 0_AcrVIA1	Kan50	This study; strain carrying the homologous recombination plasmid for removing *bas37_0130* gene from Bas37 phage
DH-E06-422	*E. coli* K-12 MG1655 ΔRM MG1655 Δ*mrr-hsdRMS-mcrBC* Δ*mcrA*	pAH210_Bas37_015 4_AcrVIA1	Kan50	This study; strain carrying the homologous recombination plasmid for removing *bas37_0154* gene from Bas37 phage
DH-E06-423	*E. coli* K-12 MG1655 ΔRM MG1655 Δ*mrr-hsdRMS-mcrBC* Δ*mcrA*	pAH210_Bas37_020 3_AcrVIA1	Kan50	This study; strain carrying the homologous recombination plasmid for removing *bas37_0203* gene from Bas37 phage
DH-E06-424	*E. coli* K-12 MG1655 ΔRM MG1655 Δ*mrr-hsdRMS-mcrBC* Δ*mcrA*	pAH210_Bas37_023 9_AcrVIA1	Kan50	This study; strain carrying the homologous recombination plasmid for removing *bas37_0239* gene from Bas37 phage
DH-E06-425	*E. coli* K-12 MG1655 ΔRM MG1655 Δ*mrr-hsdRMS-mcrBC* Δ*mcrA*	pAH210_Bas37_024 0_AcrVIA1	Kan50	This study; strain carrying the homologous recombination plasmid for removing *bas37_0240* gene from Bas37 phage
DH-E06-460	*E. coli* K-12 MG1655 ΔRM MG1655 Δ*mrr-hsdRMS-mcrBC* Δ*mcrA*	pAH210_Bas37_003 3_AcrVIA1	Kan50	This study; strain carrying the homologous recombination plasmid for removing *bas37_0033* gene from Bas37 phage

DH-E06-475	*E. coli* K-12 MG1655 ΔRM MG1655 Δ*mrr-hsdRMS-mcrBC* Δ*mcrA*	pAH210_Bas37_0260_AcrVIA1	Kan50	This study; strain carrying the homologous recombination plasmid for removing *bas37_0260* gene from Bas37 phage
DH-E06-426	*E. coli* K-12 MG1655 ΔRM MG1655 Δ*mrr-hsdRMS-mcrBC* Δ*mcrA*	pAH210_Bas54_0063_AIcrVIA3	Kan50	This study; strain carrying the homologous recombination plasmid for removing *bas54_0063* gene from Bas54 phage
DH-E06-428	*E. coli* K-12 MG1655 ΔRM MG1655 Δ*mrr-hsdRMS-mcrBC* Δ*mcrA*	pAH210_Bas54_0140_AIcrVIA3	Kan50	This study; strain carrying the homologous recombination plasmid for removing *bas54_0140* gene from Bas54 phage
DP-E06-433	*E. coli* K-12 MG1655 ΔRM MG1655 Δ*mrr-hsdRMS-mcrBC* Δ*mcrA*	pAH210_CFE299_Septu_7	Kan50	This study; the pAH210 plasmid encoding antiviral defense from CFE299 clinical isolate
DP-E06-441	*E. coli* K-12 MG1655 ΔRM MG1655 Δ*mrr-hsdRMS-mcrBC* Δ*mcrA*	pAH210_CFE299_Retron_VI	Kan50	This study; the pAH210 plasmid encoding antiviral defense from CFE299 clinical isolate
DP-E06-443	*E. coli* K-12 MG1655 ΔRM MG1655 Δ*mrr-hsdRMS-mcrBC* Δ*mcrA*	pAH210_CFE299_Septu_8	Kan50	This study; the pAH210 plasmid encoding antiviral defense from CFE299 clinical isolate
DP-E06-435	*E. coli* K-12 MG1655 ΔRM MG1655 Δ*mrr-hsdRMS-mcrBC* Δ*mcrA*	pAH210_CFE303_Druantia_III_1	Kan50	This study; the pAH210 plasmid encoding antiviral defense from CFE303 clinical isolate
DP-E06-449	*E. coli* K-12 MG1655 ΔRM MG1655 Δ*mrr-hsdRMS-mcrBC* Δ*mcrA*	pAH210_CFE331_CBASS_III_3	Kan50	This study; the pAH210 plasmid encoding antiviral defense from CFE331 clinical isolate
DP-E06-450	*E. coli* K-12 MG1655 ΔRM MG1655 Δ*mrr-hsdRMS-mcrBC* Δ*mcrA*	pAH210_CFE331_Mokosh_Type_I_A_7	Kan50	This study; the pAH210 plasmid encoding antiviral defense from CFE331 clinical isolate
DP-E06-437	*E. coli* K-12 MG1655 ΔRM MG1655 Δ*mrr-hsdRMS-mcrBC* Δ*mcrA*	pAH210_CFE336_Lamassu-Cap4_nuclease_1	Kan50	This study; the pAH210 plasmid encoding antiviral defense from CFE336 clinical isolate
DP-E06-439	*E. coli* K-12 MG1655 ΔRM MG1655 Δ*mrr-hsdRMS-mcrBC* Δ*mcrA*	pAH210_CFT073_SanaTA_8	Kan50	This study; the pAH210 plasmid encoding antiviral defense from CFT073 clinical isolate
DH-E06-469	*E. coli* K-12 MG1655 ΔRM MG1655 Δ*mrr-hsdRMS-mcrBC* Δ*mcrA*	pAH210_CFE311_DS-28_1	Kan50	This study; the pAH210 plasmid encoding antiviral defense from CFE311 clinical isolate
DH-E06-462	*E. coli* K-12 MG1655 ΔRM MG1655 Δ*mrr-hsdRMS-mcrBC* Δ*mcrA*	pNDM220_T4_alt	Amp50	This study; strain expressing the *alt* gene of T4 from a plasmid
DH-E06-461	*E. coli* K-12 MG1655 ΔRM MG1655 Δ*mrr-hsdRMS-mcrBC* Δ*mcrA*	pNDM220_Bas37_alt (0033)	Amp50	This study; strain expressing the *alt* gene of Bas37 from a plasmid
DH-E06-463	*E. coli* K-12 MG1655 ΔRM MG1655 Δ*mrr-hsdRMS-mcrBC* Δ*mcrA*	pAH210_CFE331_Mokosh_Type_I_A_7, pNDM220	Kan50, Amp50	This study; control strain carrying pNDM220 empty plasmid and plasmid expressing Mokosh type I defense system from CFE331 clinical isolate
DH-E06-464	*E. coli* K-12 MG1655 ΔRM MG1655 Δ*mrr-hsdRMS-mcrBC* Δ*mcrA*	pAH210_CFE331_Mokosh_Type_I_A_7, pNDM220_T4_alt	Kan50, Amp50	This study; strain expressing the *alt* gene of T4 from a plasmid and Mokosh type I defense system from CFE331 clinical isolate
DH-E06-465	*E. coli* K-12 MG1655 ΔRM MG1655 Δ*mrr-hsdRMS-mcrBC* Δ*mcrA*	pAH210_CFE331_Mokosh_Type_I_A_7, pNDM220_Bas37_alt(0033)	Kan50, Amp50	This study; strain expressing the *alt* gene of Bas37 from a plasmid and Mokosh type I defense system from CFE331 clinical isolate
DH-E06-476	*E. coli* K-12 MG1655 ΔRM MG1655 Δ*mrr-hsdRMS-mcrBC* Δ*mcrA*	pAH213_mcrA	Amp50	Our laboratory collection
DH-E06-477	*E. coli* K-12 MG1655 ΔRM MG1655 Δ*mrr-hsdRMS-mcrBC* Δ*mcrA*	pAH213_mcrBC	Amp50	Our laboratory collection
DH-E06-478	*E. coli* K-12 MG1655 ΔRM MG1655 Δ*mrr-hsdRMS-mcrBC* Δ*mcrA*	pAH213_mrr	Amp50	Our laboratory collection
DH-E05-404	*E. coli* K-12 MG1655 ΔRM MG1655 Δ*mrr-hsdRMS-mcrBC* Δ*mcrA*	pAH210_T4-wac-AcrVIA1_v1	Kan50	This study; strain carrying the homologous recombination plasmid for

				removing *wac* gene from T4 phage using *acrVIA1* as selection marker (including a transcriptional terminator)
DH-E07-489	*E. coli* K-12 MG1655 ΔRM MG1655 Δ*mrr-hsdRMS-mcrBC* Δ*mcrA*	pAH210_T4-wac-AcrVIA1_v3	Kan50	This study; strain carrying the homologous recombination plasmid for removing *wac* gene from T4 phage using *acrVIA1* as selection marker (lacking a transcriptional terminator)
DP-E09-722	*E. coli* EC100D K-12 *(?) F- mcrA Δ(mrr-hsdRMS-mcrBC) φ80dlacZΔM15 ΔlacX74 recA1 endA1 araD139 Δ(ara, leu)7697 galU galK λ- rpsL (StrR) nupG pir+(DHFR)*	pNDM220_T4_wac	Amp50	This study; strain expressing the *wac* gene of T4 from a plasmid
DP-E09-726	*E. coli* K-12 MG1655 ΔRM MG1655 Δ*mrr-hsdRMS-mcrBC* Δ*mcrA*	pAH221_LseCas13a, pAH218_LseCas13a_2_T4_mcp_CDS_2, pNDM220_T4_wac	Cam25, Gen20. Amp50	This study; LseCas13a selection strain with crRNA targeting T4/Bas37 phage with T4 *wac* complementation *in trans*
DP-E09-727	*E. coli* K-12 MG1655 ΔRM MG1655 Δ*mrr-hsdRMS-mcrBC* Δ*mcrA*	pAH210_T4-wac-AcrVIA1_v1, pNDM220_T4_wac	Kan50, Amp50	This study; strain carrying the homologous recombination plasmid for removing *wac* gene from T4 phage using *acrVIA1* as selection marker (including a transcriptional terminator) with T4 *wac* complementation *in trans*
DP-E09-728	*E. coli* K-12 MG1655 ΔRM MG1655 Δ*mrr-hsdRMS-mcrBC* Δ*mcrA*	pAH210_T4-wac-AcrVIA1_v3, pNDM220_T4_wac	Kan50, Amp50	This study; strain carrying the homologous recombination plasmid for removing *wac* gene from T4 phage using *acrVIA1* as selection marker (lacking a transcriptional terminator) with T4 *wac* complementation *in trans*
DH-E07-493	*E. coli* K-12 MG1655 ΔRM MG1655 Δ*mrr-hsdRMS-mcrBC* Δ*mcrA*	pAH210_Bas37_rnlA(0061)_AcrVIA1_1	Kan50	This study; strain carrying the homologous recombination plasmid for inserting *acrVIA1* gene in *bas37_0061* gene – position 1
DH-E07-490	*E. coli* K-12 MG1655 ΔRM MG1655 Δ*mrr-hsdRMS-mcrBC* Δ*mcrA*	pAH210_T4_regA_AcrVIA1	Kan50	This study; strain carrying the homologous recombination plasmid for inserting *acrVIA1* gene in T4 *regA* gene
DH-E07-491	*E. coli* K-12 MG1655 ΔRM MG1655 Δ*mrr-hsdRMS-mcrBC* Δ*mcrA*	pAH210_Bas37_regA(0161)_AcrVIA1	Kan50	This study; strain carrying the homologous recombination plasmid for inserting *acrVIA1* gene in Bas37 *bas37_0161* gene
DH-E07-494	*E. coli* K-12 MG1655 ΔRM MG1655 Δ*mrr-hsdRMS-mcrBC* Δ*mcrA*	pAH210_T4_alc_AcrVIA1	Kan50	This study; strain carrying the homologous recombination plasmid for inserting *acrVIA1* gene in T4 *alc* gene
DH-E07-495	*E. coli* K-12 MG1655 ΔRM MG1655 Δ*mrr-hsdRMS-mcrBC* Δ*mcrA*	pAH210_T4_alc_AcrVIA1_term	Kan50	This study; strain carrying the homologous recombination plasmid for inserting *acrVIA1* gene in T4 *alc* gene – including transcriptional terminator after *acrVIA1*
DH-E07-496	*E. coli* K-12 MG1655 ΔRM MG1655 Δ*mrr-hsdRMS-mcrBC* Δ*mcrA*	pAH210_T4_dsba_AcrVIA1	Kan50	This study; strain carrying the homologous recombination plasmid for inserting *acrVIA1* gene in T4 *dsba* gene
DH-E07-497	*E. coli* K-12 MG1655 ΔRM MG1655 Δ*mrr-hsdRMS-mcrBC* Δ*mcrA*	pAH210_T4_dsba_AcrVIA1_term	Kan50	This study; strain carrying the homologous recombination plasmid for inserting *acrVIA1* gene in T4 *dsba* gene – including transcriptional terminator after *acrVIA1*
DH-E07-498	*E. coli* K-12 MG1655 ΔRM MG1655 Δ*mrr-hsdRMS-mcrBC* Δ*mcrA*	pAH210_T4_gp47.1_AcrVIA1	Kan50	This study; strain carrying the homologous recombination plasmid for inserting *acrVIA1* gene in T4 *gp47.1* gene
DH-E07-499	*E. coli* K-12 MG1655 ΔRM MG1655 Δ*mrr-hsdRMS-mcrBC* Δ*mcrA*	pAH210_T4_gp47.1_AcrVIA1_term	Kan50	This study; strain carrying the homologous recombination plasmid for inserting *acrVIA1* gene in T4 *gp47.1*

				gene – including transcriptional terminator after *acrVIA1*
DH-E07-500	*E. coli* K-12 MG1655 ΔRM MG1655 Δ*mrr-hsdRMS-mcrBC* Δ*mcrA*	pAH210_T4_gp47.1_AcrVIA1_fw	Kan50	This study; strain carrying the homologous recombination plasmid for inserting *acrVIA1* gene in T4 *gp47.1* gene in opposite direction of the disrupted gene
DH-E07-501	*E. coli* K-12 MG1655 ΔRM MG1655 Δ*mrr-hsdRMS-mcrBC* Δ*mcrA*	pAH210_T4_upgp42_AcrVIA1	Kan50	This study; strain carrying the homologous recombination plasmid for inserting *acrVIA1* gene upstream of T4 *gp42* gene
DH-E07-502	*E. coli* K-12 MG1655 ΔRM MG1655 Δ*mrr-hsdRMS-mcrBC* Δ*mcrA*	pAH210_T4_upgp42_AcrVIA1_term	Kan50	This study; strain carrying the homologous recombination plasmid for inserting *acrVIA1* gene upstream of T4 *gp42* gene – including transcriptional terminator after *acrVIA1*
DH-E07-503	*E. coli* K-12 MG1655 ΔRM MG1655 Δ*mrr-hsdRMS-mcrBC* Δ*mcrA*	pAH210_T4_upgp42_AcrVIA1_fw	Kan50	This study; strain carrying the homologous recombination plasmid for inserting *acrVIA1* gene upstream of T4 *gp42* gene in opposite direction of the disrupted gene
DH-E07-506	*E. coli* K-12 MG1655 ΔRM MG1655 Δ*mrr-hsdRMS-mcrBC* Δ*mcrA*	pNDM220_Bas54_0009	Amp50	This study; strain expressing the *bas54_0009* gene of Bas54 from a plasmid
DH-E07-507	*E. coli* K-12 MG1655 ΔRM MG1655 Δ*mrr-hsdRMS-mcrBC* Δ*mcrA*	pNDM220_Bas54_0087	Amp50	This study; strain expressing the *bas54_0087* gene of Bas54 from a plasmid
DH-E07-508	*E. coli* K-12 MG1655 ΔRM MG1655 Δ*mrr-hsdRMS-mcrBC* Δ*mcrA*	pNDM220_Bas54_0110	Amp50	This study; strain expressing the *bas54_0110* gene of Bas54 from a plasmid
DH-E07-509	*E. coli* K-12 MG1655 ΔRM MG1655 Δ*mrr-hsdRMS-mcrBC* Δ*mcrA*	pNDM220_Bas54_0184	Amp50	This study; strain expressing the *bas54_0184* gene of Bas54 from a plasmid
DH-E07-505	*E. coli* K-12 MG1655 ΔRM MG1655 Δ*mrr-hsdRMS-mcrBC* Δ*mcrA*	pNDM220_Bas54_0202	Amp50	This study; strain expressing the *bas54_0202* gene of Bas54 from a plasmid
DH-E07-504	*E. coli* K-12 MG1655 ΔRM MG1655 Δ*mrr-hsdRMS-mcrBC* Δ*mcrA*	pNDM220_Bas54_0220	Amp50	This study; strain expressing the *bas54_0220* gene of Bas54 from a plasmid
DP-E10-732	*E. coli* K-12 MG1655 ΔRM MG1655 Δ*mrr-hsdRMS-mcrBC* Δ*mcrA*	pUA139-Bas54-0009	Kan50	This study; strain carrying the homologous recombination plasmid for deleting *bas54_0009* gene in Bas54 phage
DP-E10-734	*E. coli* K-12 MG1655 ΔRM MG1655 Δ*mrr-hsdRMS-mcrBC* Δ*mcrA*	pUA139-Bas54-0087	Kan50	This study; strain carrying the homologous recombination plasmid for deleting *bas54_0087* gene in Bas54 phage
DP-E10-776	*E. coli* K-12 MG1655 ΔRM MG1655 Δ*mrr-hsdRMS-mcrBC* Δ*mcrA*	pUA139-Bas54-0110	Kan50	This study; strain carrying the homologous recombination plasmid for deleting *bas54_0110* gene in Bas54 phage
DP-E10-736	*E. coli* K-12 MG1655 ΔRM MG1655 Δ*mrr-hsdRMS-mcrBC* Δ*mcrA*	pUA139-Bas54-0184	Kan50	This study; strain carrying the homologous recombination plasmid for deleting *bas54_0184* gene in Bas54 phage
DP-E10-778	*E. coli* K-12 MG1655 ΔRM MG1655 Δ*mrr-hsdRMS-mcrBC* Δ*mcrA*	pUA139-Bas54-0202	Kan50	This study; strain carrying the homologous recombination plasmid for deleting *bas54_0202* gene in Bas54 phage
DP-E10-751	*E. coli* K-12 MG1655 ΔRM MG1655 Δ*mrr-hsdRMS-mcrBC* Δ*mcrA*	pUA139-Bas54-0220	Kan50	This study; strain carrying the homologous recombination plasmid for deleting *bas54_0220* gene in Bas54 phage

DP-E10-758	*E. coli* K-12 MG1655 ΔRM MG1655 Δ*mrr-hsdRMS-mcrBC* Δ*mcrA*	pAH212-Bas54-0009 + DS-SpCas	Cam25, Sp50	This study; CRISPR-Cas9 selection strain
DP-E10-759	*E. coli* K-12 MG1655 ΔRM MG1655 Δ*mrr-hsdRMS-mcrBC* Δ*mcrA*	pAH212-Bas54-0087 + DS-SpCas	Cam25, Sp50	This study; CRISPR-Cas9 selection strain
DP-E10-774	*E. coli* K-12 MG1655 ΔRM MG1655 Δ*mrr-hsdRMS-mcrBC* Δ*mcrA*	pAH212-Bas54-0110 + DS-SpCas	Cam25, Sp50	This study; CRISPR-Cas9 selection strain
DP-E10-760	*E. coli* K-12 MG1655 ΔRM MG1655 Δ*mrr-hsdRMS-mcrBC* Δ*mcrA*	pAH212-Bas54-0184 + DS-SpCas	Cam25, Sp50	This study; CRISPR-Cas9 selection strain
DP-E10-761	*E. coli* K-12 MG1655 ΔRM MG1655 Δ*mrr-hsdRMS-mcrBC* Δ*mcrA*	pAH212-Bas54-0202 + DS-SpCas	Cam25, Sp50	This study; CRISPR-Cas9 selection strain
DP-E10-762	*E. coli* K-12 MG1655 ΔRM MG1655 Δ*mrr-hsdRMS-mcrBC* Δ*mcrA*	pAH212-Bas54-0220 + DS-SpCas	Cam25, Sp50	This study; CRISPR-Cas9 selection strain
DP-E10-770	*E. coli* K-12 MG1655 ΔRM MG1655 Δ*mrr-hsdRMS-mcrBC* Δ*mcrA*	pUA139-Bas54-0009, pNDM220_Bas54_0009	Kan50, Amp50	This study; strain carrying the homologous recombination plasmid for deleting *bas54_0009* gene in Bas54 phage + complementation of the gene
DP-E10-771	*E. coli* K-12 MG1655 ΔRM MG1655 Δ*mrr-hsdRMS-mcrBC* Δ*mcrA*	pUA139-Bas54-0087, pNDM220_Bas54_0087	Kan50, Amp50	This study; strain carrying the homologous recombination plasmid for deleting *bas54_0087* gene in Bas54 phage + complementation of the gene
DP-E10-779	*E. coli* K-12 MG1655 ΔRM MG1655 Δ*mrr-hsdRMS-mcrBC* Δ*mcrA*	pUA139-Bas54-0110. pNDM220_Bas54_0110	Kan50, Amp50	This study; strain carrying the homologous recombination plasmid for deleting *bas54_0110* gene in Bas54 phage + complementation of the gene
DP-E10-772	*E. coli* K-12 MG1655 ΔRM MG1655 Δ*mrr-hsdRMS-mcrBC* Δ*mcrA*	pUA139-Bas54-0184, pNDM220_Bas54_0184	Kan50, Amp50	This study; strain carrying the homologous recombination plasmid for deleting *bas54_0184* gene in Bas54 phage + complementation of the gene
DP-E10-782	*E. coli* K-12 MG1655 ΔRM MG1655 Δ*mrr-hsdRMS-mcrBC* Δ*mcrA*	pUA139-Bas54-0202, pNDM220_Bas54_0202	Kan50, Amp50	This study; strain carrying the homologous recombination plasmid for deleting *bas54_0202* gene in Bas54 phage + complementation of the gene
DP-E10-773	*E. coli* K-12 MG1655 ΔRM MG1655 Δ*mrr-hsdRMS-mcrBC* Δ*mcrA*	pUA139-Bas54-0220, pNDM220_Bas54_0220	Kan50, Amp50	This study; strain carrying the homologous recombination plasmid for deleting *bas54_0220* gene in Bas54 phage + complementation of the gene
DP-E10-766	*E. coli* K-12 MG1655 ΔRM MG1655 Δ*mrr-hsdRMS-mcrBC* Δ*mcrA*	pAH212-Bas54-0009 + DS-SpCas, pNDM220_Bas54_0009	Cam25, Sp50, Amp50	This study; CRISPR-Cas9 selection strain + complementation of the gene
DP-E10-767	*E. coli* K-12 MG1655 ΔRM MG1655 Δ*mrr-hsdRMS-mcrBC* Δ*mcrA*	pAH212-Bas54-0087 + DS-SpCas, pNDM220_Bas54_0087	Cam25, Sp50, Amp50	This study; CRISPR-Cas9 selection strain + complementation of the gene
DP-E10-779	*E. coli* K-12 MG1655 ΔRM MG1655 Δ*mrr-hsdRMS-mcrBC* Δ*mcrA*	pAH212-Bas54-0110 + DS-SpCas, pNDM220_Bas54_0110	Cam25, Sp50, Amp50	This study; CRISPR-Cas9 selection strain + complementation of the gene
DP-E10-768	*E. coli* K-12 MG1655 ΔRM MG1655 Δ*mrr-hsdRMS-mcrBC* Δ*mcrA*	pAH212-Bas54-0184 + DS-SpCas, pNDM220_Bas54_0184	Cam25, Sp50, Amp50	This study; CRISPR-Cas9 selection strain + complementation of the gene
DP-E10-781	*E. coli* K-12 MG1655 ΔRM MG1655 Δ*mrr-hsdRMS-mcrBC* Δ*mcrA*	pAH212-Bas54-0202 + DS-SpCas, pNDM220_Bas54_0202	Cam25, Sp50, Amp50	This study; CRISPR-Cas9 selection strain + complementation of the gene
DP-E10-769	*E. coli* K-12 MG1655 ΔRM MG1655 Δ*mrr-hsdRMS-mcrBC* Δ*mcrA*	pAH212-Bas54-0220 + DS-SpCas, pNDM220_Bas54_0220	Cam25, Sp50, Amp50	This study; CRISPR-Cas9 selection strain + complementation of the gene
AH-E04-284	*E. coli* CFT073 *rpoS(+)*	none	none	Our laboratory collection

CFE-299	*E. coli* isolate CFE299	none	none	Collection of Swiss NCCR AntiResist consortium (phylogroup B2, ST80, O7-type O-antigen)
CFE-303	*E. coli* isolate CFE303	none	none	Collection of Swiss NCCR AntiResist consortium (phylogroup B2, ST1161, O170-type O-antigen)
CFE-311	*E. coli* isolate CFE311	none	none	Collection of Swiss NCCR AntiResist consortium (phylogroup F, ST648, O1-type O-antigen)
CFE-331	*E. coli* isolate CFE331	none	none	Collection of Swiss NCCR AntiResist consortium (phylogroup B2, ST73, O25-type O-antigen)
CFE-336	*E. coli* isolate CFE336	none	none	Collection of Swiss NCCR AntiResist consortium (phylogroup D, ST349, O166-type O-antigen)

Supplementary Table 2. List of all oligonucleotide primers and synthesized DNA used in this study

Name	Purpose	Sequence (5'-3')
prAH2173	Used for PCR amplification; see Supplementary Data 3	GATCTGATAGAGAAGGGTTTGC
prAH2179	Used for PCR amplification; see Supplementary Data 3	GTCATTTCGAACCCCAGAGT
prDH0264	Used for PCR amplification; see Supplementary Data 3	GCGGGACTCTGGGGTTCGAAATGAC
prDH0265	Used for PCR amplification; see Supplementary Data 3	ACGAGCAAACCCTTCTCTATCAGATC
prDH0347	Used for PCR amplification; see Supplementary Data 3	TGGCTGATAAGTCCCCGGTCTACGAAGCATGAGACCGATAGGTAAACGA
prDH0349	Used for PCR amplification; see Supplementary Data 3	CTATCGGTCTCATGCTTAGACCGGGGACTTATCAGCCAA
prDH0263	Used for PCR amplification; see Supplementary Data 3	CTGACGACCGCGCAAGTGGCACTTT
prDH0454	Used for PCR amplification; see	TGAGACCGATAGGTAAACGA

	Supplementary Data 3	
prDH0233	Used for PCR amplification; see Supplementary Data 3	TCCAGTCGGTAGATATTCCACAAAACAGC
prDH0261	Used for PCR amplification; see Supplementary Data 3	GCTGTTTTGTGGAATATCTACCGACTGG
prDH0262	Used for PCR amplification; see Supplementary Data 3	CCCGAAAAGTGCCACTTGCG
prDH0343	Used for PCR amplification; see Supplementary Data 3	TACCTAGGACTGAGCTAGCCGTAAATCTCGAGTCATTTCGAACCC
prDH0344	Used for PCR amplification; see Supplementary Data 3	TTTACGGCTAGCTCAGTCCTAGGTACAATGCTAGCGGATCCTCTAGATTTTTAAGAAGGAGATATA
prAH2568	Used for PCR amplification; see Supplementary Data 3	CAACTTAATCGCCTTGCAGC
prAH2245	Used for PCR amplification; see Supplementary Data 3	TCCAATTGTTATCCGCTCAC
prAH2525	Used for PCR amplification; see Supplementary Data 3	CAATTGTGAGCGGATAACAATTGGACTATGACCATGATTACGAATTCG
prAH2569	Used for PCR amplification; see Supplementary Data 3	GATGTGCTGCAAGGCGATTAAGTTGGTATACACTCCGCTATCGCTACC
prJR19	Used for PCR amplification; see Supplementary Data 3	TAATTTGACCAGAGAACAAGAATAACACTTTTCGGGGAAATGTTCTA
prJR21	Used for PCR amplification; see Supplementary Data 3	GCATAGTCCCTAGGACTGAGCTAGCTGTCAAGTTAATGTCATGATAATAATGGTTTCTTAGAC
prJR22	Used for PCR amplification; see Supplementary Data 3	AGCTAGCTCAGTCCTAGGGACTATGCTAGCTAAGAAGGAGATATACATATGAAGAATGGTTTTTATGCG
prJR18	Used for PCR amplification;	TTATTCTTGTTCTCTGGTCAAATTA

	see Supplementary Data 3	
prAH2038	Used for PCR amplification; see Supplementary Data 3	GAGAGAAGATTTTCAGCCTGATAC
prAH2711	Used for PCR amplification; see Supplementary Data 3	TGTATCAGGCTGAAAATCTTCTCTCCTGCAAGGTAGTGGACAAGAC
prDH0210	Used for PCR amplification; see Supplementary Data 3	CGCTAGTTTGTTATCAGAATCGCAGAGCGAGCTCGATATCAAATTAC
prDH0204	Used for PCR amplification; see Supplementary Data 3	AAGTAAAGTGATTAACAGCGCATTAGAGCTGCTTAATGAGGTC
prDH0205	Used for PCR amplification; see Supplementary Data 3	CTAATGCGCTGTTAATCACTTTAC
prDH0206	Used for PCR amplification; see Supplementary Data 3	CTATTGGAATTCACGGTACTTTCAATTTCAT
prDH0207	Used for PCR amplification; see Supplementary Data 3	AAATTGAAAGTACCGTGAATTCCAATAGC
prDH0208	Used for PCR amplification; see Supplementary Data 3	GACTGTAACAGCTCTTCAGATACATACAGAG
prDH0209	Used for PCR amplification; see Supplementary Data 3	GTATGTATCTGAAGAGCTGTTACAGTCCTTG
prDH0153	Used for PCR amplification; see Supplementary Data 3	CTGCGATTCTGATAACAAACTAGC
prAH2697	Used for PCR amplification; see Supplementary Data 3	GCGGGACTCTGGGGTTCGAAATGACGGAATTCTTGACAGCTAGCTCA
prAH2698	Used for PCR amplification; see Supplementary Data 3	CGAGCAAACCCTTCTCTATCAGATCCCTTTCGTTTTATTTGATGCC

prDH0292	Used for cloning **crRNA** against T4/Bas37; see Supplementary Data 3	AAAC**TTCAAAAGTTCAGCTTTAGTTTTG**A
prDH0293	Used for cloning **crRNA** against T4/Bas37; see Supplementary Data 3	AGCAT**CAAAACTAAAGCTGAACTTTTGAA**
prDH0184	Used for cloning **crRNA** against Bas54; see Supplementary Data 3	AAAC**AACTTCAAAATCCTTATAAAGAGTTTTCTTT**A
prDH0185	Used for cloning **crRNA** against Bas54; see Supplementary Data 3	AGCAT**AAAGAAAACTCTTTATAAGGATTTTGAAGTT**
prAH2224	Used for PCR amplification; see Supplementary Data 3	CCCTTTGATATGTAACGGTGAAC
prAH2225	Used for PCR amplification; see Supplementary Data 3	GTTAATGTCATGATAATAATGGTTTCTTAGAC
prDH0509	Used for PCR amplification; see Supplementary Data 3	GCAAAACCCGTACCCTAGGT
prDH0154	Used for PCR amplification; see Supplementary Data 3	TCGAGATTGACAGCTAGCTCA
prDH0523	Used for PCR amplification; see Supplementary Data 3	AAACCATTATTATCATGACATTAACCTTCGTGGATAAGAGAAAAGAGTG
prDH0521	Used for PCR amplification; see Supplementary Data 3	CCTAGACCTAGGGTACGGGTTTTGCGAAATTGTGATTGTTGATTGGG
prDH0522	Used for PCR amplification; see Supplementary Data 3	GGACTGAGCTAGCTGTCAATCTCGAGCCCTTGATATATTTTACAATCATCTA
prDH0524	Used for PCR amplification; see Supplementary Data 3	CTGTTCACCGTTACATATCAAAGGGTGCGTCAGCAGATACATCATATAC
prDH0507	Used for PCR amplification; see	CGGGACTCTGGGGTTCGAAATGACTCACCCCATTCAGTCTGTAAT

	Supplementary Data 3	
prDH0508	Used for PCR amplification; see Supplementary Data 3	CCTAGACCTAGGGTACGGGTTTTGCTGGTAGATTCTTATAATCGAGGATTG
prDH0510	Used for PCR amplification; see Supplementary Data 3	GGACTGAGCTAGCTGTCAATCTCGACCTATACATAAAACCAAAATTTTAGTCAT
prDH0511	Used for PCR amplification; see Supplementary Data 3	CGAGCAAACCCTTCTCTATCAGATCCCTCTTATAATTCGGGATGGA
prDH0526	Used for PCR amplification; see Supplementary Data 3	AAACCATTATTATCATGACATTAACCACCTGTCATTGTAACCAATTCA
prDH0527	Used for PCR amplification; see Supplementary Data 3	CCTAGACCTAGGGTACGGGTTTTGCCATTTTGCCACGTGGATTAA
prDH0528	Used for PCR amplification; see Supplementary Data 3	GGACTGAGCTAGCTGTCAATCTCGAGCGGTATTTTCTTCTTTGGATATAAG
prDH0529	Used for PCR amplification; see Supplementary Data 3	CTGTTCACCGTTACATATCAAAGGGCATGATTGAATTTATAGATGCATATGC
prDH0530	Used for PCR amplification; see Supplementary Data 3	AAACCATTATTATCATGACATTAACGGCTCCATACCTAAACGTCG
prDH0531	Used for PCR amplification; see Supplementary Data 3	CCTAGACCTAGGGTACGGGTTTTGCATGAGAAAGTCGGTGATAAACAG
prDH0532	Used for PCR amplification; see Supplementary Data 3	GGACTGAGCTAGCTGTCAATCTCGATTTACGTTTCATTACAATTTCCTCA
prDH0533	Used for PCR amplification; see Supplementary Data 3	CTGTTCACCGTTACATATCAAAGGGTATAGCAATGCTCTCTATGCATAAAG
prDH0534	Used for PCR amplification; see Supplementary Data 3	AAACCATTATTATCATGACATTAACCCTCATCAGTATGTAAACAACTTTGT
prDH0535	Used for PCR amplification;	CCTAGACCTAGGGTACGGGTTTTGCTGTCTTATAATGTTGGGCCC

	see Supplementary Data 3	
prDH0536	Used for PCR amplification; see Supplementary Data 3	GGACTGAGCTAGCTGTCAATCTCGAAAATTCCATCATTCTTCATCTTTTG
prDH0537	Used for PCR amplification; see Supplementary Data 3	CTGTTCACCGTTACATATCAAAGGGGATCAAGCTGCTCGTCTGAT
prDH0538	Used for PCR amplification; see Supplementary Data 3	AAACCATTATTATCATGACATTAACTAGTCAGAAGCTCAGCAATTTTCT
prDH0539	Used for PCR amplification; see Supplementary Data 3	CCTAGACCTAGGGTACGGGTTTTGCGACGAGACTTTTAAACAAGTACTAGGA
prDH0540	Used for PCR amplification; see Supplementary Data 3	GGACTGAGCTAGCTGTCAATCTCGACAGTCTCCATATATTCTTTACGCTTT
prDH0541	Used for PCR amplification; see Supplementary Data 3	CTGTTCACCGTTACATATCAAAGGGTGATGATTATAGCCCTCCTGTTA
prDH0546	Used for PCR amplification; see Supplementary Data 3	AAACCATTATTATCATGACATTAACTGCTTCCATTGCATAAGTCTTT
prDH0547	Used for PCR amplification; see Supplementary Data 3	CCTAGACCTAGGGTACGGGTTTTGCTGATGGTGCATCAGATGATC
prDH0548	Used for PCR amplification; see Supplementary Data 3	GGACTGAGCTAGCTGTCAATCTCGATAATAGTCTTAAACAATTTTTCTGGATTATC
prDH0549	Used for PCR amplification; see Supplementary Data 3	CTGTTCACCGTTACATATCAAAGGGTCTAGTTGAACGATACGAAATCG
prDH0550	Used for PCR amplification; see Supplementary Data 3	AAACCATTATTATCATGACATTAACAAGTCTATCACGCAACCGAT
prDH0551	Used for PCR amplification; see Supplementary Data 3	CCTAGACCTAGGGTACGGGTTTTGCTCGATTTAAACGATGTTGATTATAT

prDH0552	Used for PCR amplification; see Supplementary Data 3	GGACTGAGCTAGCTGTCAATCTCGATATTCATCGTGAACGGATTTTC
prDH0553	Used for PCR amplification; see Supplementary Data 3	CTGTTCACCGTTACATATCAAAGGGCTCAGATGATGTAAGTGCATCTG
prDH0542	Used for PCR amplification; see Supplementary Data 3	AAACCATTATTATCATGACATTAACCCAACTAAACCGCTATTTACAATAAC
prDH0543	Used for PCR amplification; see Supplementary Data 3	CCTAGACCTAGGGTACGGGTTTTGCGACGATGTAGTTAAAGAAGAAATTTTC
prDH0544	Used for PCR amplification; see Supplementary Data 3	GGACTGAGCTAGCTGTCAATCTCGACAGAACCAGGACAGCCAATA
prDH0545	Used for PCR amplification; see Supplementary Data 3	CTGTTCACCGTTACATATCAAAGGGCCAGATTCAGCGGTTAGAATC
prDH0554	Used for PCR amplification; see Supplementary Data 3	AAACCATTATTATCATGACATTAACGCTGCGTTATCAAGCTTTTC
prDH0555	Used for PCR amplification; see Supplementary Data 3	CCTAGACCTAGGGTACGGGTTTTGCCAGAAGACAAAGTAGATGGTTCAAC
prDH0556	Used for PCR amplification; see Supplementary Data 3	GGACTGAGCTAGCTGTCAATCTCGAAACAAAATAGATATTTCTTTTCTTTTTCAA
prDH0557	Used for PCR amplification; see Supplementary Data 3	CTGTTCACCGTTACATATCAAAGGGTTGTGTATTCACTAGTGAAATCAGAG
prDH0558	Used for PCR amplification; see Supplementary Data 3	AAACCATTATTATCATGACATTAACGGTCATCAATAGCTAATTTCACATC
prDH0559	Used for PCR amplification; see Supplementary Data 3	CCTAGACCTAGGGTACGGGTTTTGCAACCAAAGAAGACCCAACAA
prDH0560	Used for PCR amplification; see	GGACTGAGCTAGCTGTCAATCTCGATAAAACCCAGGATTTTTAGCAATA

	Supplementary Data 3	
prDH0561	Used for PCR amplification; see Supplementary Data 3	CTGTTCACCGTTACATATCAAAGGGATACTCGCGAACTCAATGAAC
prDH0562	Used for PCR amplification; see Supplementary Data 3	AAACCATTATTATCATGACATTAACAAGGTTCCATACCTAAACGTCG
prDH0563	Used for PCR amplification; see Supplementary Data 3	CCTAGACCTAGGGTACGGGTTTTGCTATTTCGTGAAATTACTGAAGATGG
prDH0564	Used for PCR amplification; see Supplementary Data 3	GGACTGAGCTAGCTGTCAATCTCGACGTCATTAGTGCAGTTCTGAAC
prDH0565	Used for PCR amplification; see Supplementary Data 3	CTGTTCACCGTTACATATCAAAGGGTCGCAAAAATTGGGATTGTG
prDH0566	Used for PCR amplification; see Supplementary Data 3	AAACCATTATTATCATGACATTAACCCGTCTTTGTCTCGGTAAAC
prDH0567	Used for PCR amplification; see Supplementary Data 3	CCTAGACCTAGGGTACGGGTTTTGCAGTTGTCAGAAGCAGAAATGC
prDH0568	Used for PCR amplification; see Supplementary Data 3	GGACTGAGCTAGCTGTCAATCTCGATCTTTAGAACGTCTTTTTGCAGAT
prDH0569	Used for PCR amplification; see Supplementary Data 3	CTGTTCACCGTTACATATCAAAGGGGCATTTTTCATAAAGTTGTTTACAAG
prDH0570	Used for PCR amplification; see Supplementary Data 3	AAACCATTATTATCATGACATTAACAGCTTTAATCAAACGGGATTTT
prDH0571	Used for PCR amplification; see Supplementary Data 3	CCTAGACCTAGGGTACGGGTTTTGCTGAAGAAGACGAGCCATTAAAG
prDH0572	Used for PCR amplification; see Supplementary Data 3	GGACTGAGCTAGCTGTCAATCTCGAAACATATCAAGGGCAGAAACTG
prDH0573	Used for PCR amplification;	CTGTTCACCGTTACATATCAAAGGGTCGTCAGTATAAAGCAGGTTCTATT

	see Supplementary Data 3	
prDH0574	Used for PCR amplification; see Supplementary Data 3	AAACCATTATTATCATGACATTAACCATTAATTGCGTAATAACCATATGC
prDH0575	Used for PCR amplification; see Supplementary Data 3	CCTAGACCTAGGGTACGGGTTTTGCGGAAGTTTGAAGAACGGTTAAAG
prDH0576	Used for PCR amplification; see Supplementary Data 3	GGACTGAGCTAGCTGTCAATCTCGAAAATTTGTCCTCCATCAACTGT
prDH0577	Used for PCR amplification; see Supplementary Data 3	CTGTTCACCGTTACATATCAAAGGGTACAAGACTGTTCCTCTGTGGTATT
prDH0578	Used for PCR amplification; see Supplementary Data 3	AAACCATTATTATCATGACATTAACCACATCAGTAGAAATTATCTGACGC
prDH0579	Used for PCR amplification; see Supplementary Data 3	CCTAGACCTAGGGTACGGGTTTTGCCGAAGCATGAAAATAAACGTATTC
prDH0580	Used for PCR amplification; see Supplementary Data 3	GGACTGAGCTAGCTGTCAATCTCGATTTACCTCTAGAAAATCTTCCGG
prDH0581	Used for PCR amplification; see Supplementary Data 3	CTGTTCACCGTTACATATCAAAGGGGGAGTAAATAGGAATGAAAATGAAA
prDH0582	Used for PCR amplification; see Supplementary Data 3	AAACCATTATTATCATGACATTAACTCAAACATTGAATTGAAATCACTTT
prDH0583	Used for PCR amplification; see Supplementary Data 3	CCTAGACCTAGGGTACGGGTTTTGCCTTGCTCGAGTTGAAACCTT
prDH0584	Used for PCR amplification; see Supplementary Data 3	GGACTGAGCTAGCTGTCAATCTCGACAGCAGTATACTGTTCTTCGACAG
prDH0585	Used for PCR amplification; see Supplementary Data 3	CTGTTCACCGTTACATATCAAAGGGCAAAAGGCGATGTTGAAATA

prDH0634	Used for PCR amplification; see Supplementary Data 3	AAACCATTATTATCATGACATTAACATTTATCCTTGAACGAACTTGTAAG
prDH0635	Used for PCR amplification; see Supplementary Data 3	CCTAGACCTAGGGTACGGGTTTTGCAGAGTTGACTTTGCATCATTTG
prDH0636	Used for PCR amplification; see Supplementary Data 3	GGACTGAGCTAGCTGTCAATCTCGAAGGTGCATAAACGAAAGCCT
prDH0637	Used for PCR amplification; see Supplementary Data 3	CTGTTCACCGTTACATATCAAAGGGCTAGATTATTAAAGGCCTTCGGG
prDH0646	Used for PCR amplification; see Supplementary Data 3	AAACCATTATTATCATGACATTAACTTTTCTGCTGTAGTCAATAATCCAC
prDH0647	Used for PCR amplification; see Supplementary Data 3	CCTAGACCTAGGGTACGGGTTTTGCGGGCAGCAGAAAATAAATCAA
prDH0648	Used for PCR amplification; see Supplementary Data 3	GGACTGAGCTAGCTGTCAATCTCGATTTCATTTTGATTTCCATTTGGT
prDH0649	Used for PCR amplification; see Supplementary Data 3	CTGTTCACCGTTACATATCAAAGGGTGAAGCTGAGCGTCTTTTCT
prDH0586	Used for PCR amplification; see Supplementary Data 3	AAACCATTATTATCATGACATTAACCAACGTCATAGCCGATCTGT
prDH0587	Used for PCR amplification; see Supplementary Data 3	GTCATTTCGAACCCCAGAGTCCCGCCTTCTACAGGGATAGGTAGCCC
prDH0588	Used for PCR amplification; see Supplementary Data 3	ATCTGATAGAGAAGGGTTTGCTCGTAACCGAGATTTTGTATTCTCAGTCT
prDH0589	Used for PCR amplification; see Supplementary Data 3	CTGTTCACCGTTACATATCAAAGGGCCTTAAGCTTTAGCCAAAATGG
prDH0598	Used for PCR amplification; see	AAACCATTATTATCATGACATTAACGCTACGCTCGAAAGAGAGACT

	Supplementary Data 3	
prDH0599	Used for PCR amplification; see Supplementary Data 3	ATCTGATAGAGAAGGGTTTGCTCGTTTCAGCTACTCCAACCTTGATAA
prDH0600	Used for PCR amplification; see Supplementary Data 3	GTCATTTCGAACCCCAGAGTCCCGCGGAGTATTTAGCCGCCTTTG
prDH0601	Used for PCR amplification; see Supplementary Data 3	CTGTTCACCGTTACATATCAAAGGGTTCTTGCCAAACTTGACACTG
prDP0266	Used for PCR amplification; see Supplementary Data 3	AAACCATTATTATCATGACATTAACGCAAAACAATATCGCGTCC
prDP0267	Used for PCR amplification; see Supplementary Data 3	CTGTTCACCGTTACATATCAAAGGGTGGATGTTCTCAGCCTGTC
prDP0280	Used for PCR amplification; see Supplementary Data 3	AAACCATTATTATCATGACATTAACACTTCTTAGCGGTGATTCTG
prDP0281	Used for PCR amplification; see Supplementary Data 3	CTGTTCACCGTTACATATCAAAGGGGAGACCTTCGAGTTATCAATC
prDP0284	Used for PCR amplification; see Supplementary Data 3	AAACCATTATTATCATGACATTAACTGGTAACGCGATCATCAAG
prDP0285	Used for PCR amplification; see Supplementary Data 3	CTGTTCACCGTTACATATCAAAGGGTAAAGGATGGAGTAAAGTAGCG
prDP0270	Used for PCR amplification; see Supplementary Data 3	AAACCATTATTATCATGACATTAACGAGGATGTGGAGTGAGGGG
prDP0271	Used for PCR amplification; see Supplementary Data 3	CTGTTCACCGTTACATATCAAAGGGTTTATAGAAAATACCGCTCCCG
prDP0296	Used for PCR amplification; see Supplementary Data 3	AAACCATTATTATCATGACATTAACATTGAGTCAGGCAGCATAAG
prDP0297	Used for PCR amplification;	CTGTTCACCGTTACATATCAAAGGGCAAATGTACCGGAATTGGAG

	see Supplementary Data 3	
prDP0298	Used for PCR amplification; see Supplementary Data 3	AAACCATTATTATCATGACATTAACGAACGATTACATCAGTATCATACC
prDP0299	Used for PCR amplification; see Supplementary Data 3	CTGTTCACCGTTACATATCAAAGGGCTCATTTGACATGGTTAACG
prDP0274	Used for PCR amplification; see Supplementary Data 3	AAACCATTATTATCATGACATTAACAAATACGCCCACGAAGTCCC
prDP0275	Used for PCR amplification; see Supplementary Data 3	CTGTTCACCGTTACATATCAAAGGGAAAAATGTTAGGCCTGCAAGC
prDP0272	Used for PCR amplification; see Supplementary Data 3	AAACCATTATTATCATGACATTAACATTAGCAGACAGGTAATGTGG
prDP0273	Used for PCR amplification; see Supplementary Data 3	CTGTTCACCGTTACATATCAAAGGGCGGAATGAAACTGATTGTATAAC
prJR46	Used for PCR amplification; see Supplementary Data 3	AAACCATTATTATCATGACATTAACCATAATCCGGGTCAATGTAA
prJR47	Used for PCR amplification; see Supplementary Data 3	CTGTTCACCGTTACATATCAAAGGGGCAAGGCGGCTTTTATATAT
prDH0640	Used for PCR amplification; see Supplementary Data 3	ACAACGTCGTGACTGGGAAAAC
prDH0633	Used for PCR amplification; see Supplementary Data 3	AAAACGACGGCCAGTGAATTC
prDH0632	Used for PCR amplification; see Supplementary Data 3	CGAGGAATTCACTGGCCGTCGTTTTGCAGGAGGAATTCACCATGATGGAACTTATTACAGAATTATTTGAC
prDH0639	Used for PCR amplification; see Supplementary Data 3	AGGGTTTTCCCAGTCACGACGTTGTTTATCCTTGAACGAACTTGTAAGG

prAH1948	Used for PCR amplification; see Supplementary Data 3	GGGAACGCGGCCGCACCTACATCTGTATTAACGAAGCG, with NotI restriction site
prAH1949	Used for PCR amplification; see Supplementary Data 3	CTGCTTCTCGAGACACGGTGCCTGACTGC; with XhoI restriction site in the primer and adjacent ClaI + HindIII restriction sites on the amplified backbone
prAH1954	Used for PCR amplification; see Supplementary Data 3	CGAAACAAGCTTTTAAGGCCCATAACATCTCA; with HindIII restriction site
prAH1955	Used for PCR amplification; see Supplementary Data 3	GCATTTGCGGCCGCTCACTCAAAATAGTCCATATCCAG; NotI restriction site
prAH1958	Used for PCR amplification; see Supplementary Data 3	GGGAATATCGATGTAAACAGAAAGTCGCGTAACAG; with ClaI restriction site
prAH1959	Used for PCR amplification; see Supplementary Data 3	GCATTTGCGGCCGCTTATTTGAGATATTCATCGAAAATGT; with NotI restriction site
prAH1960	Used for PCR amplification; see Supplementary Data 3	GGGAACATCGATACAAAAGCTGGACTGGAAAC; with ClaI restriction site
prAH1961	Used for PCR amplification; see Supplementary Data 3	GCATTTGCGGCCGCTTATTTCTGTAATCGGTTTATATTAACG; with NotI restriction site
prDH0514	Used for PCR amplification; see Supplementary Data 3	AAACCATTATTATCATGACATTAACATTGGAAATGAAACACGTCC
prDH0515	Used for PCR amplification; see Supplementary Data 3	GGACTGAGCTAGCTGTCAATCTCGACCAATGTTTTTATCAAGAACTTCA
prDH0516	Used for PCR amplification; see Supplementary Data 3	CCTAGACCTAGGGTACGGGTTTTGCCGGTTCAACTGTTGAAGAGC
prDH0517	Used for PCR amplification; see Supplementary Data 3	CTGTTCACCGTTACATATCAAAGGGGGTGATAAAAAGGTAGAAAGCAATAC
prDH0676	Used for PCR amplification; see	GCAAAACCCGTACCCTAGGTCTAGGCCTCTAGAACATTTCCCCGA

	Supplementary Data 3	
prDP0515	Used for PCR amplification; see Supplementary Data 3	CGAGGAATTCACTGGCCGTCGTTTTATGACAGATATTGTACTGAATGACTTAC
prDP0516	Used for PCR amplification; see Supplementary Data 3	AGGGTTTTCCCAGTCACGACGTTGTTTATGCTGGTGATAAAAAGG
prDH0682	Used for PCR amplification; see Supplementary Data 3	AAACCATTATTATCATGACATTAACCGCTTCCATCGCATAAGTCTTT
prDH0683	Used for PCR amplification; see Supplementary Data 3	CTGTTCACCGTTACATATCAAAGGGTCTAGTTGAACGATTCGAAATCG
prDH0677	Used for PCR amplification; see Supplementary Data 3	ACGTCTAAGAAACCATTATTATCATGACATTAACAACTCGGAAGTTATTAGTTAAATCTTTC
prDH0678	Used for PCR amplification; see Supplementary Data 3	AGCTAGTTTGTTATCAGAATCGCAGTATCAGTCCTGTCATATTCTTCAGA
prDH0679	Used for PCR amplification; see Supplementary Data 3	GGACTGAGCTAGCTGTCAATCTCGAAGAACTTTATCTTTATTATTAGCAATTCCC
prDH0680	Used for PCR amplification; see Supplementary Data 3	CTGTTCACCGTTACATATCAAAGGGGTCACGGATGATTTTTTGAAA
prDH0701	Used for PCR amplification; see Supplementary Data 3	AAACCATTATTATCATGACATTAACAAGCATGCCTCAACATCTTCTG
prDH0702	Used for PCR amplification; see Supplementary Data 3	AGCTAGTTTGTTATCAGAATCGCAGTATGCTTCCTGATGCGGTTG
prDH0699	Used for PCR amplification; see Supplementary Data 3	GGACTGAGCTAGCTGTCAATCTCGATCACGGGCTTGCTCAAGAC
prDH0700	Used for PCR amplification; see Supplementary Data 3	CTGTTCACCGTTACATATCAAAGGGAAGCATACGGTGATACTACAGATGGG
prDH0703	Used for PCR amplification;	CCTAGACCTAGGGTACGGGTTTTGCTATGCTTCCTGATGCGGTTG

	see Supplementary Data 3	
prDH0705	Used for PCR amplification; see Supplementary Data 3	AAACCATTATTATCATGACATTAACCCAAACTCTTCTACCATTTTTTC
prDH0704	Used for PCR amplification; see Supplementary Data 3	AGCTAGTTTGTTATCAGAATCGCAGTAGCTTTGTATCATAAAGATAACCG
prDH0706	Used for PCR amplification; see Supplementary Data 3	GGACTGAGCTAGCTGTCAATCTCGAATAGACGATTAAACATCTTACCATC
prDH0707	Used for PCR amplification; see Supplementary Data 3	CTGTTCACCGTTACATATCAAAGGGATTTTGAGGTGAATAATGGC
prDH0708	Used for PCR amplification; see Supplementary Data 3	CCTAGACCTAGGGTACGGGTTTTGCTAGCTTTGTATCATAAAGATAACCG
prDH0712	Used for PCR amplification; see Supplementary Data 3	AAACCATTATTATCATGACATTAACTATCACCGTATTGAATCCAGG
prDH0711	Used for PCR amplification; see Supplementary Data 3	AGCTAGTTTGTTATCAGAATCGCAGTATTCACTAGAGCATGTTGGATTG
prDH0709	Used for PCR amplification; see Supplementary Data 3	GGACTGAGCTAGCTGTCAATCTCGAGATAAAATGCAAGAAAAAGTATCACA
prDH0710	Used for PCR amplification; see Supplementary Data 3	CTGTTCACCGTTACATATCAAAGGGTATTAAAGAACTGTCATCTGACCG
prDH0713	Used for PCR amplification; see Supplementary Data 3	CCTAGACCTAGGGTACGGGTTTTGCTATTCACTAGAGCATGTTGGATTG
prDH0714	Used for PCR amplification; see Supplementary Data 3	GGACTGAGCTAGCTGTCAATCTCGATATTCACTAGAGCATGTTGGATTG
prDH0715	Used for PCR amplification; see Supplementary Data 3	AGCTAGTTTGTTATCAGAATCGCAGGATAAAATGCAAGAAAAAGTATCACA

prDH0717	Used for PCR amplification; see Supplementary Data 3	AAACCATTATTATCATGACATTAACGAATGTATTCATCATTAAGAGCG
prDH0716	Used for PCR amplification; see Supplementary Data 3	GCTAGTTTGTTATCAGAATCGCAGTATTATTTTAGGTTGGAGTTTATTACTGTTA
prDH0718	Used for PCR amplification; see Supplementary Data 3	GGACTGAGCTAGCTGTCAATCTCGAGTTGGAATCATAAGAGGAACCG
prDH0719	Used for PCR amplification; see Supplementary Data 3	CTGTTCACCGTTACATATCAAAGGGGTTTAACTGGTCAATTATTGGTTGG
prDH0720	Used for PCR amplification; see Supplementary Data 3	CCTAGACCTAGGGTACGGGTTTTGCTATTATTTTAGGTTGGAGTTTATTACTGTTA
prDH0721	Used for PCR amplification; see Supplementary Data 3	GGACTGAGCTAGCTGTCAATCTCGATATTATTTTAGGTTGGAGTTTATTACTGTTA
prDH0722	Used for PCR amplification; see Supplementary Data 3	AGCTAGTTTGTTATCAGAATCGCAGGTTGGAATCATAAGAGGAACCG
prDH0684	Used for PCR amplification; see Supplementary Data 3	GTGAATTCCTCCTGCAAAACGA
prDH0685	Used for PCR amplification; see Supplementary Data 3	CCGTCGTTTTGCAGGAGGAATTCACCATGATTACTCGTAAAGATGAATTTG
prDH0686	Used for PCR amplification; see Supplementary Data 3	AGGGTTTTCCCAGTCACGACGTTGTTTATCCACCTTTCAGAGTTGC
prDH0687	Used for PCR amplification; see Supplementary Data 3	CCGTCGTTTTGCAGGAGGAATTCACCATGAGCGTTATTCAACAATTCG
prDH0688	Used for PCR amplification; see Supplementary Data 3	AGGGTTTTCCCAGTCACGACGTTGTTTACAATGCGTGGATTCTACTTTT
prDH0689	Used for PCR amplification; see	CCGTCGTTTTGCAGGAGGAATTCACCATGATCAGCGGGGCAAGTTT

	Supplementary Data 3	
prDH0690	Used for PCR amplification; see Supplementary Data 3	AGGGTTTTCCCAGTCACGACGTTGTCGGAAGCATGAGATCGCTTTAC
prDH0691	Used for PCR amplification; see Supplementary Data 3	CCGTCGTTTTGCAGGAGGAATTCACCATGGCTCACTTTGTTGGTGA
prDH0692	Used for PCR amplification; see Supplementary Data 3	AGGGTTTTCCCAGTCACGACGTTGTTTAGCTTTCAATACCGAATAATTTTTC
prDH0695	Used for PCR amplification; see Supplementary Data 3	CCGTCGTTTTGCAGGAGGAATTCACCATGAAACTTGATAAAAATGTTCAACG
prDH0696	Used for PCR amplification; see Supplementary Data 3	AGGGTTTTCCCAGTCACGACGTTGTTCAATCAAGGACAACACCG
prDH0697	Used for PCR amplification; see Supplementary Data 3	CCGTCGTTTTGCAGGAGGAATTCACCATGGCGGATACAACTAAAAAG
prDH0698	Used for PCR amplification; see Supplementary Data 3	AGGGTTTTCCCAGTCACGACGTTGTTTAGCTATTGCTAACAGGCTGTA
prDP0011	Used for PCR amplification; see Supplementary Data 3	ATGTATATCTCCTTCTTAAATCTAGACTCG
prDP0012V2	Used for PCR amplification; see Supplementary Data 3	TGAAAGCGCTATTTCTTCCAG
prDP0531	Used for PCR amplification; see Supplementary Data 3	CTAGATTTAAGAAGGAGATATACATAGATGGAAATTACACTGACAGAG
prDP0532	Used for PCR amplification; see Supplementary Data 3	CATATGGGATGAATCACCTTAGTGATTATTTCTCCAAAGTAAAACTTAA
prDP0533	Used for PCR amplification; see Supplementary Data 3	TCACTAAGGTGATTCATCCC
prDP0534	Used for PCR amplification;	AATTCTGGAAGAAATAGCGCTTTCATTTCATCAAGGTTCTCTGTG

	see Supplementary Data 3	
prDP0535	Used for PCR amplification; see Supplementary Data 3	CTAGATTTAAGAAGGAGATATACATATGAACGAGCAAGAACAACC
prDP0536	Used for PCR amplification; see Supplementary Data 3	ATGCATGAATTCTACTTTTGCCATATTATTTGTCTCCTTGTTCTTCG
prDP0537	Used for PCR amplification; see Supplementary Data 3	TATGGCAAAAGTAGAATTCAT
prDP0538	Used for PCR amplification; see Supplementary Data 3	AATTCTGGAAGAAATAGCGCTTTCACCCTTTGAAATCATCTATCC
prDP0539	Used for PCR amplification; see Supplementary Data 3	CTAGATTTAAGAAGGAGATATACATCCAAATATTATGCCCCTACC
prDP0540	Used for PCR amplification; see Supplementary Data 3	CAATCTTCTCAAAATTAATCTTCATTACTTTCTCCTTTCATTGTATTG
prDP0541	Used for PCR amplification; see Supplementary Data 3	ATGAAGATTAATTTTGAGAAGATTG
prDP0576	Used for PCR amplification; see Supplementary Data 3	AATTCTGGAAGAAATAGCGCTTTCAGGTAATTTGCGATCATAATG
prDP0543	Used for PCR amplification; see Supplementary Data 3	CTAGATTTAAGAAGGAGATATACATGTGACCAATTGCCGTTACC
prDP0544	Used for PCR amplification; see Supplementary Data 3	TCTATAATAATGGCTCACTTTGTTGGGTGGTGAAGATGAGTCATAAC
prDP0545	Used for PCR amplification; see Supplementary Data 3	CAACAAAGTGAGCCATTATTA
prDP0546	Used for PCR amplification; see Supplementary Data 3	AATTCTGGAAGAAATAGCGCTTTCAAAAAGGCAGATAGTGTGGC

prDP0577	Used for PCR amplification; see Supplementary Data 3	CTAGATTTAAGAAGGAGATATACATTTCACCTGCAAACACGACAC
prDP0552	Used for PCR amplification; see Supplementary Data 3	ATGAGTAAACTGGAGAGTATGAAAATCATTGACATAACCACACGG
prDP0553	Used for PCR amplification; see Supplementary Data 3	TTTTCATACTCTCCAGTTTACTC
prDP0554	Used for PCR amplification; see Supplementary Data 3	AATTCTGGAAGAAATAGCGCTTTCAGAAAGATAAGAATGATGAAGG
prDP0555	Used for PCR amplification; see Supplementary Data 3	CTAGATTTAAGAAGGAGATATACATTCTACCCTGAGCCTGATAAC
prDP0556	Used for PCR amplification; see Supplementary Data 3	AAGCAAAGTTTGTCAAGAAAATATTGTTTCCTCCATTATCACGTG
prDP0557	Used for PCR amplification; see Supplementary Data 3	AATATTTTCTTGACAAACTTTGC
prDP0558	Used for PCR amplification; see Supplementary Data 3	AATTCTGGAAGAAATAGCGCTTTCATGATTTTCGAGCAGTTAGTG
prDH0512	To screen for T4 *vs* and *tk.4* knockout mutant	GAATAAACGATCCAATTCAGCTT
prDH0513	To screen for T4 *vs* knockout mutant	CTATGCCTGCACCAATCCTA
prDH0318	To screen for recombinant phages; anneals in *acrVIA1*	ACTTCTGTCTCGTTGTGCGT
prDH0614	To screen for T4 *alt* knockout mutant	ATGGATTATGGGAAAATGCTC
prDH0615	To screen for T4 *pseT* knockout mutant and T4 *alc* mutant	AACAGTTCATCCTAATTGGCCT
prDH0616	To screen for T4/Bas37 *rnlA* mutants	ATCTGCATTAATTCATTCTGCG
prDH0727	To screen for Bas37/T4 *rnlA* mutant	GTATGCTTCAACGACCATTT

prDH0617	To screen for T4 *cef* knockout mutant	CATTCAGCTTAAGCATGGTGTT
prDH0618	To screen for T4 *nrdC.1* knockout mutant	GAATTATGGTCTTTCACTGTTTGAA
prDH0619	To screen for T4 *57B* knockout mutant	TCAAGATCATGAAATGGATGC
prDH0620	To screen for Bas37 *0053* and *0055* knockout mutant	CAGACCGTTACATTGATGCA
prDH0621	To screen for Bas37 *0119* knockout mutant	GGGTAGAAGATGGCGTTAAGTTA
prDH0622	To screen for Bas37 *0130* knockout mutant	CATCGCTATCGTAATATGGAATTAG
prDH0623	To screen for Bas37 *0154* knockout mutant	CTCGACGTGGTATCATGATTTATAC
prDH0624	To screen for Bas37 *0203* knockout mutant	TGGTGACAAGAAAGGCTCATA
prDH0625	To screen for Bas37 *0239 and 0240* knockout mutant	AGGTTGTGATTAACATGTTCACTG
prDH0638	To screen for Bas37 *0033* knockout mutant	TAAAGAATTCGCCGAAGTAGC
prDH0650	To screen for Bas37 *0260* knockout mutant	AGGTGCTACACACGATTTTGAT
prDH0626	To screen for Bas54 *0063* knockout mutant	AAAGTTAGAGGGCAAGCAAG
prDH0628	To screen for Bas54 *0140* knockout mutant	GTCAAGCTGGATTAATCAAGAAG
prDH0520	To screen for T4 *wac* knockout mutant	GGTTCTCACTTAACAAATCCAAATG
prDH0525	To screen for T4 *wac* knockout mutant	TTATAAGCACCCTGTTTAACATTCG
prDH0681	To screen for Bas37/T4 *regA* mutant	CTTCATCACCACCTTCACAGT
prDH0726	To screen for Bas37/T4 *regA* mutant	AAACTGGTTGAAGATTCCACC
prDH0731	To screen for T4 *alc* mutant	CATATCCGATGCACTACGATTT

prDH0729	To screen for T4 *dsba* mutant	GCAAATTGAGTTTCAGGAATAGAA
prDH0724	To screen for T4 *dsba* mutant	AAAGGATAACGTTGCTTCAGTTAAA
prDH0728	To screen for T4 *gp47.1* mutant	AAGCTCAGTAGAAGCATTTGGAT
prDH0723	To screen for T4 *gp47.1* mutant	TGATAAAATGAATCTCGCGC
prDH0730	To screen for T4 upstream *gp42* mutant	ATACCACGTCGAGAATCAGG
prDH0725	To screen for T4 upstream *gp42* mutant	GAAAACTACTGCGCTGAGCT
prDP0559	To check for deletion of *bas54_0009*	ATTCTGATATCGTGCTCGGC
prDP0560	To check for deletion of *bas54_0009*	TTTGAAAGCAGCATCTACCTC
prDP0561	To check for deletion of *bas54_0087*	AATTGGGGAACTTTCGAAG
prDP0562	To check for deletion of *bas54_0087*	ACATTTTTGCACCGTCTACC
prDP0563	To check for deletion of *bas54_0110*	GGACGATGAAGAATACCAGG
prDP0564	To check for deletion of *bas54_0110*	CCTTTCTCTCTTTGATGTGG
prDP0565	To check for deletion of *bas54_0184*	TACATCCATCATACAACCGC
prDP0566	To check for deletion of *bas54_0184*	GAAATCCTTAAACCGGGTC
prDP0569	To check for deletion of *bas54_0202*	ACTCTTCTTCACAGCGTTTC
prDP0570	To check for deletion of *bas54_0202*	CAGAGGTGAAAGTTATTGACG
prDP0571	To check for deletion of *bas54_0220*	TTATGGGCTATGTTGTTGG
prDP0572	To check for deletion of *bas54_0220*	GACTGTAACATTTGATAATCCG
pAH160-P5univ-Fw-PAGE	P7 primer for the second PCR during library preparation of all HIDEN-SEQ libraries	AATGATACGGCGACCACCGAGATCTACACTCTTTCCCTACACGACGCTCTTCCGATCTcgtagaccggggacttatc
pAH160-Biot-Fw	Biotinylated primer for the first PCR during library preparation of HIDEN-SEQ libraries with	ccgggagtctgcgaagttag

	AcrVIA1 as selection marker	
pDH1-Biot-Fw	Biotinylated primer for the first PCR during library preparation of HIDEN-SEQ libraries with AIcrVIA3 as selection marker	gccgaggagtataatctgaaagttg
AcrVIA1	Synthesized DNA sequence of AcrVIA1 for cloning pAH160_Tn_AcrVIA1_v2	GCTGTTTTGTGGAATATCTACCGACTGGAAACAGCGATGTTCCGGCCTGCCAGACTGGACGGACATAACAGGTTGGCTGATAA GTCCCCGGTCTGCGGGACTCTGGGGTTCGAAATGACTCGAGATTGACAGCTAGCTCAGTCCTAGGTATAATGCTAGCGGATCC TCTAGATTTTTTAAGAAGGAGATATACATATGATTTACTACATTAAAGATTTGAAGGTAAAGGGGAAAATTTTCGAGAATCTCA TGAACAAAGAGGCCGTCGAAGGCCTGATAACCTTTCTGAAGAAGGCAGAGTTCGAGATATATTCTCGGGAGAACTACTCCAAA TATAATAAATGGTTTGAGATGTGGAAGTCGCCTACCAGCTCACTGGTCTTCTGGAAGAATTACTCATTTCGTTGTCACCTGTTGT TCGTGATCGAGAAAGACGGTGAATGTTTAGGTATTCCAGCGTCAGTCTTTGAAAGCGTATTGCAGATCTATCTGGCTGATCCCT TTGCTCCGGACACTAAGGAATTGTTCGTGGAAGTATGTAACTTATACGAGTGTCTGGCAGACGTGACGGTAGTTGAGCACTTT GAAGCAGAGGAGTCTGCTTGGCATAAGCTGACGCACAACGAGACAGAAGTCTCAAAGCGCGTCTACTCTAAGGACGATGACG AGTTGCTGAAATATATACCTGAGTTCTTAGACACAATTGCCACGAACAAGAAGTCTCAAAAGTACAATCAAATTCAAGGTAAAA TACAGGAGATAAACAAGGAGATCGCCACTTTATACGAGTCTAGTGAGGACTACATATTCACGGAGTATGTATCAAACCTCTACC GGGAGTCTGCGAAGTTAGAACAACACTCGAAACAAATACTGAAGGAAGAGTTAAACTAACTGATTAGAAACAGAGACCTCGTT TACCTATCGGTCTCATGCTGATCTGATAGAGAAGGGTTTGCTCGTAGACCGGGGACTTATCAGCCAACCTGTTATGTGGCGCAG CTAGTTTGTTATCAGAATCGCAGACGACAAGCTGACGACCGCGCAAGTGGCACTTTTCGGG
transcriptional terminator	Synthesized DNA sequence of transcriptional terminator for cloning pAH160_Tn_AcrVIA1_v3	ATACTGAAGGAAGAGTTAAACTAACTGATTAGAAACAGAGACCTCGTTTACCTATCGGTCTCATGCTGTCGGGGAAATGTTCTA GAGGCATCAAATAAAACGAAAGGCTCAGTCGAAAGACTGGGCCTTTCGTTTTATCTGTTGTTTGTCGGTGAACGCTCTCCTGAG TAGGACAAATCCGCCGCCCTAGACCTAGGGTACGGGTTTTGCATCTGATAGAGAAGGGTTTGCTCGTAGACCGGGGACTTATC AGCCAACCTGTTATGTGGCGCAGGTAAACCAGCAATAGACATAAGCGGGAACCGACGACAAGCTGACGACCGCGCAAGTGGC ACTTTTCGGG
sup-tRNAs	Synthesized DNA sequence of *sup-tRNAs* for cloning pAH160_Tn_sup-tRNAs_LE392_v2	GCTGTTTTGTGGAATATCTACCGACTGGAAACAGCGATGTTCCGGCCTGCCAGACTGGACGGACATAACAGGTTGGCTGATAA GTCCCCGGTCTGCGGGACTCTGGGGTTCGAAATGACCTAACCAAACAGTCACTTTCGAGCAATTTTCCTTGAAAAAGAGGTTGA CGCTGCAAGGCTCTATACGCATAATGCGCCCCGCAACGCCGATAAGGTATCGCGAAAAAAAAGATGGTGGGGTTCCCGAGCG GCCAAAGGGAGCAGACTCTAAATCTGCCGTCATCGACTTCGAAGGTTCGAATCCTTCCCCCACCACCATCGAAGAAACAATCTA TTTATTCAAGACGCTTACCTTGTAAGTGCACCCAGTTGGGGTATCGCCAAGCGGTAAGGCACCGGATTCTAATTCCGGCATTCC GAGGTTCGAATCCTCGTACCCCAGCCACATTAAAAAAGCTCGCTTCGGCGAGCTTTTTGCTTTTCTGCGTTCATTCAATGTCGAA TGCGATGTTGAAACAGAGACCTCGTTTACCTATCGGTCTCATGCTGATCTGATAGAGAAGGGTTTGCTCGTAGACCGGGGACTT ATCAGCCAACCTGTTATGTGGCGCAGGTAAACCAGCAATAGACATAAGCGGGAACCGACGACAAGCTGACGACCGCGCAAGT GGCACTTTTCGGG
AIcrVIA3	Synthesized DNA sequence of AIcrVIA3 for cloning pAH160_Tn_AIcrVIA3_v2	GCTGTTTTGTGGAATATCTACCGACTGGAAACAGCGATGTTCCGGCCTGCCAGACTGGACGGACATAACAGGTTGGCTGATAA GTCCCCGGTCTGCGGGACTCTGGGGTTCGAAATGACTCGAGATTGACAGCTAGCTCAGTCCTAGGTATAATGCTAGCGGATCC TCTAGATTTTTTAAGAAGGAGATATACATATGGCACCTAAAAAAATACGTCGTCACCGTAACAATTCCTGTGACAGATGCAGACC TGACGGTTTTTCTGGTTGTCGATATAATAGTGTATGCGGAGAAACTGGGCGGTACGGTGACAATTACAGCCGTGAAATCCGAA AATGACTCTTATAGCGTTACTCTGGAAGACCTGGATAAAGCGGCCGAAGAATTAGAAAAAGTCGGTGGTAGCATTGTATTGAC TGTTACATTTGATAATAAAGAGAAAGCAGAGAAAGTGGCAGAGTTTGCTTACCTGAAAGCCGAGGAGTATAATCTGAAAGTTG ATGTTGAAGTTAAAGAGGAACTGTAACTGATTAGAAACAGAGACCTCGTTTACCTATCGGTCTCATGCTGATCTGATAGAGAA GGGTTTGCTCGTAGACCGGGGACTTATCAGCCAACCTGTTATGTGGCGCAGCTAGTTTGTTATCAGAATCGCAGACGACAAGC TGACGACCGCGCAAGTGGCACTTTTCGGG
LseCas13a fragment I	Synthesized DNA sequence of LseCas13a for cloning pAH221_LseCas13a	CTAATGCGCTGTTAATCACTTTACTTTTATCTAATCTAGACATCATTAATTCCTAATTTTTGTTGACACTCTATCGTTGATAGAGTT ATTTTACCACTCCCTATCAGTGATAGAGAAAAGAATTCAAAAGATCTAAAGAGGAGAAAGGATCTTACATGTGGATTTCCATTA AGACACTGATTCATCACCTGGGAGTTCTGTTTTTTTGTGATTACATGTATAATCGTCGGGAAAAGAAAATTATTGAAGTTAAAAC CATGCGCATTACGAAAGTAGAAGTTGATAGAAAAAAAGTTCTTATTTCCCGCGATAAAAACGGTGGCAAGTTAGTGTACGAAA ATGAAATGCAGGATAATACTGAACAGATCATGCACCATAAAAAATCATCATTCTACAAATCTGTGGTTAACAAAACAATCTGTC GACCAGAGCAGAAACAGATGAAGAAGCTGGTGCATGGATTGTTACAGGAAAATTCACAGGAAAAAATCAAAGTTAGTGATGT TACAAAGCTCAATATTTCCAATTTCCTGAATCACCGCTTCAAGAAGAGTTTATACTATTTCCCCGAGAATTCGCCTGATAAGTCC GAGGAATATCGCATCGAGATCAATTTGAGCCAACTGTTAGAGGATTCTCTGAAAAAACAGCAAGGTACATTTATTTGTTGGGA ATCCTTTAGTAAAGATATGGAACTGTATATTAACTGGGCCGAAAATTATATTAGTTCAAAGACGAAACTGATTAAAAAAAGTAT CCGCAACAATCGGATACAGAGCACTGAATCGAGATCCGGGCAGCTCATGGATAGATATATGAAAGACATACTGAATAAGAAC AAACCTTTTGATATCCAGTCCGTCTCAGAAAAATATCAATTAGAAAAACTGACCTCTGCTTTGAAGGCAACCTTTAAAGAAGCTA AAAAAAACGACAAAGAAATTAATTACAAACTCAAATCAACGCTGCAGAACCATGAACGGCAGATTATAGAGGAACTTAAAGAG AACTCGGAGCTTAACCAGTTTAACATCGAAATACGCAAACATCTGGAAACCTACTTCCCAATTAAAAAAACCAATCGTAAAGTA

		GGAGATATTCGAAATCTTGAAATCGGCGAAATCCAGAAAATCGTCAATCATCGTCTTAAGAACAAAATTGTGCAGCGCATCCTGCAGGAGGGCAAATTGGCGTCTTATGAAATTGAAAGTACCGTGAATTCCAATAG
LseCas13a fragment II	Synthesized DNA sequence of LseCas13a for cloning pAH221_LseCas13a	AAATTGAAAGTACCGTGAATTCCAATAGCCTCCAGAAAATTAAAATTGAAGAGGCGTTCGCCCTTAAATTTATTAATGCATGCTTATTCGCGTCCAACAATCTGCGCAACATGGTATATCCTGTTTGTAAAAAAGACATTCTTATGATTGGTGAATTCAAGAATTCTTTTAAGGAAATTAAACATAAAAAATTTATTCGACAGTGGTCCCAGTTCTTTTCCCAAGAAATTACCGTGGACGACATTGAGCTTGCTAGCTGGGGTCTGCGTGGTGCTATCGCACCAATTAGAAATGAGATCATTCACCTCAAGAAACACTCATGGAAAAAATTCTTCAACAACCCAACATTCAAGGTGAAAAAGTCCAAAATCATAAATGGGAAAACCAAAGATGTGACCAGCGAGTTTCTCTATAAAGAGACCCTGTTCAAGGATTATTTCTATAGCGAGCTGGACAGCGTCCCAGAGCTCATTATTAACAAGATGGAGTCATCAAAAATATTAGATTATTACAGCTCTGATCAGCTCAATCAGGTGTTTACCATTCCAAATTTCGAACTGTCACTTCTTACCTCAGCTGTTCCGTTTGCACCATCGTTTAAACGTGTTTATCTGAAAGGATTTGATTATCAGAATCAGGATGAGGCACAACCGGACTACAATTTGAAATTAAATATATACAATGAAAAAGCCTTTAATAGTGAAGCCTTTCAAGCACAATATTCACTCTTTAAAATGGTGTATTATCAGGTGTTTCTGCCGCAATTTACAACGAATAATGATCTGTTTAAATCGAGCGTGGATTTTATCCTGACACTGAATAAGGAACGCAAGGGATATGCCAAAGCGTTTCAAGATATTAGAAAAATGAACAAAGATGAAAAACCTTCAGAGTACATGTCATATATTCAGAGTCAACTGATGCTGTATCAAAAAAAGCAGGAAGAAAAGGAGAAAATTAATCATTTTGAGAAATTTATTAACCAGGTGTTTATAAAAGGTTTTAATTCCTTTATTGAAAAGAATCGTCTGACCTATATATGTCATCCTACCAAAAATACGGTACCAGAAAACGATAATATCGAAATTCCGTTTCATACGGACATGGATGACTCAAACATTGCTTTTTGGTTAATGTGCAAACTGCTTGATGCGAAACAGCTGAGTGAGCTCCGTAACGAGATGATCAAATTCTCATGTTCCTTGCAGTCCACCGAGGAAATTAGCACGTTCACTAAGGCCCGCGAGGTCATTGGATTGGCATTACTCAACGGTGAAAAAGGCTGCAATGATTGGAAGGAATTATTTGATGATAAAGAAGCCTGGAAAAAAAATATGTCTCTGTATGTATCTGAAGAGCTGTTACAGTC
LseCas13a fragment III	Synthesized DNA sequence of LseCas13a for cloning pAH221_LseCas13a	GTATGTATCTGAAGAGCTGTTACAGTCCTTGCCCTACACGCAGGAAGATGGTCAGACGCCCGTTATTAATCGCTCTATCGACCTCGTTAAGAAATACGGCACAGAAACCATTTTAGAAAAATTGTTCTCATCTTCGGACGACTACAAAGTCTCTGCGAAAGATATTGCAAAGTTGCATGAGTATGACGTAACAGAAAAAATTGCACAGCAGGAAAGCTTGCATAAACAGTGGATCGAAAAACCTGGACTGGCACGTGATTCAGCGTGGACAAAGAAATATCAAAATGTCATAAATGATATTTCTAATTATCAATGGGCCAAAACTAAAGTAGAACTTACCCAAGTTCGCCATCTTCATCAGTTAACGATTGATTTGTTGAGTCGTCTGGCGGGCTACATGTCTATTGCAGATCGTGACTTTCAGTTTAGCTCCAATTATATCTTGGAAAGAGAGAATAGCGAGTACCGTGTTACCTCCTGGATTCTGCTGTCTGAGAACAAGAATAAGAACAAGTACAACGACTACGAGCTGTATAATCTTAAAAACGCGTCCATCAAAGTCAGTAGCAAGAATGATCCGCAACTTAAAGTTGATCTGAAGCAGCTGCGACTGACACTCGAATATTTAGAGCTGTTTGATAACAGATTAAAAGAAAAGCGTAATAATATTAGCCACTTCAATTACTTAAATGGACAGTTAGGTAACAGTATATTGGAATTATTCGACGACGCCCGCGATGTACTGAGCTATGATCGTAAGCTGAAGAATGCCGTTTCCAAATCGCTGAAAGAAATTTTATCCTCTCATGGCATGGAAGTTACATTTAAGCCCTTATACCAAACCAACCATCATCTCAAAATCGACAAATTACAACCTAAGAAAATCCATCACCTGGGTGAGAAATCAACTGTATCTTCGAATCAGGTATCTAACGAGTACTGTCAACTTGTCAGAACCCTGTTAACAATGAAGTAATGAATCCCTCGGTACCAAAGACGAACAATAAGACGCTGAAAAGCGTCTTTTTTCGTTTTGGTCCGCTGAGCAGTTACAGAGATGTTACGAACCACTAGTGCACTGCAGTACACCTTTGGTCGAAAAAAAAAGCCCGCACTGTCAGGTGCGGGCTTTTTTCTGTGTTTCCACCAATAAAAAACGCCCGGCGGCAACCGAGCGTTCTGAACAAATCCAGATGGAGTTCTGAGGTCATTACTGGATCTATCACGCTAGTTTGTTATCAGAATCGCAG
crRNA LseCas13a	Synthesized DNA sequence of LseCas13a crRNA cassette for cloning the entry vector pAH218_LseCas13a	GGAATTCTTGACAGCTAGCTCAGTCCTAGGTATAATACTAGTGTAAGAGACTACCTCTATATGAAAGAGGACTAAAACAGAGACCTCGTTTACCTATCGGTCTCATGCTTGGGCCCGAACAAAAACTCATCTCAGAAGAGGATCTGAATAGCGCCGTCGACCATCATCATCATCATCATTGAGTTTAAACGCTCTCCAGCTTGGGTGTTTTGGCGGATGAGAGAAGATTTTCAGCCTGATACAGATTAAATCAGAACGCAGAAGCGGTCTGATAAAACAGAATTTGCCTGGCGGCAGTAGCGCGGTGGTCCCACCTGACCCCATGCCGAACTCAGAAGTGAAACGCCGTAGCGCCGATGGTAGTGTGGGGTCTGCCCATGCGAGAGTAGGCAACTGCCAGGCATCAAATAAAACGAAAGG

Supplementary References

1. Miller, E. S. *et al.* Bacteriophage T4 Genome. *Microbiology and Molecular Biology Reviews* **67**, 86–156 (2003).
2. Chen, J. *et al.* Systematic, high-throughput characterization of bacteriophage gene essentiality on diverse hosts. *Cell Host & Microbe* **33**, 1363-1380.e11 (2025).
3. Wood, W. B. & Conley, M. P. Attachment of tail fibers in bacteriophage T4 assembly: Role of the phage whiskers. *Journal of Molecular Biology* **127**, 15–29 (1979).
4. van Opijnen, T., Bodi, K. L. & Camilli, A. Tn-seq: high-throughput parallel sequencing for fitness and genetic interaction studies in microorganisms. *Nat Methods* **6**, 767–772 (2009).
5. Chan, A. *et al.* Bacteriophage genome-wide transposon mutagenesis. 2025.11.23.690004 Preprint at https://doi.org/10.1101/2025.11.23.690004 (2025).
6. Kyte, N., Fuchs, M., Smith, L. M. & Fineran, P. C. High-throughput transposon mutagenesis defines the essential genome of diverse phages. 2025.12.19.695335 Preprint at https://doi.org/10.64898/2025.12.19.695335 (2025).
7. Tiemann, B., Depping, R. & Rüger, W. Overexpression, Purification, and Partial Characterization of ADP-Ribosyltransferases ModA and ModB of Bacteriophage T4. *Gene Expression* **8**, 187 (2018).
8. Lehman, I. R. & Pratt, E. A. On the Structure of the Glucosylated Hydroxymethylcytosine Nucleotides of Coliphages T2, T4, and T6. *Journal of Biological Chemistry* **235**, 3254–3259 (1960).
9. Miravet-Verde, S., Burgos, R., Garcia-Ramallo, E., Weber, M. & Serrano, L. Quantitative essentiality in a reduced genome: a functional, regulatory and structural fitness map. *Molecular Systems Biology* 1–29 (2025) doi:10.1038/s44320-025-00133-1.
10. Maffei, E., Shaidullina A. *et al.* Systematic exploration of Escherichia coli phage–host interactions with the BASEL phage collection. *PLOS Biology* **19**, e3001424 (2021).
11. Datsenko, K. A. & Wanner, B. L. One-step inactivation of chromosomal genes in Escherichia coli K-12 using PCR products. *Proceedings of the National Academy of Sciences of the United States of America* **97**, 6640 (2000).
12. Hobbs, S. J. *et al.* Phage anti-CBASS and anti-Pycsar nucleases subvert bacterial immunity. *Nature* **605**, 522–526 (2022).
13. LeRoux, M. *et al.* The DarTG toxin-antitoxin system provides phage defence by ADP-ribosylating viral DNA. *Nat Microbiol* **7**, 1028–1040 (2022).
14. Baba, T. *et al.* Construction of Escherichia coli K-12 in-frame, single-gene knockout mutants: the Keio collection. *Molecular Systems Biology* **2**, 2006.0008 (2006).

Source Data Supplementary Figures

Source Data Supplementary Fig. 3

Unprocessed agarose gels

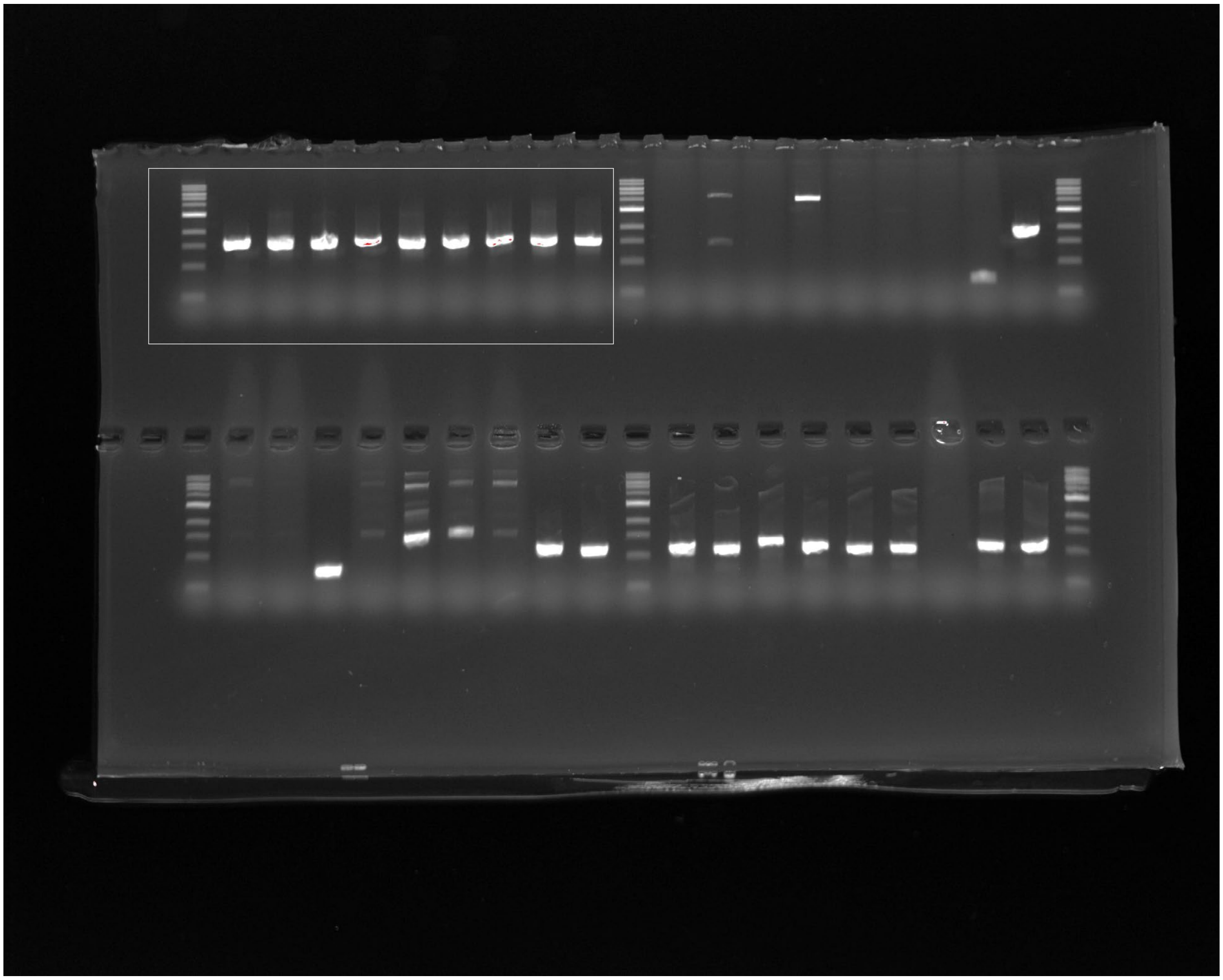

Supplement: Supplementary file 2 [file 41564_2026_2455_MOESM1_ESM.pdf]
