# Supplementary material for: Systematic mapping of bacteriophage gene essentiality with HIDEN-SEQ
Source: Nat Microbiol. 2026 Sep 25;11(10):2751–63. doi: 10.1038/s41564-026-02455-8 (PMC13716836; doi:10.1038/s41564-026-02455-8)
Supplement: Supplementary file 3 [file 41564_2026_2455_MOESM2_ESM.pdf]

nature portfolio

Corresponding author(s): Alexander Harms

Last updated by author(s): Jul 3, 2026

Reporting Summary

Nature Portfolio wishes to improve the reproducibility of the work that we publish. This form provides structure for consistency and transparency in reporting. For further information on Nature Portfolio policies, see our Editorial Policies and the Editorial Policy Checklist.

Statistics

For all statistical analyses, confirm that the following items are present in the figure legend, table legend, main text, or Methods section.

n/a	Confirmed	
☐	☒	The exact sample size (*n*) for each experimental group/condition, given as a discrete number and unit of measurement
☐	☒	A statement on whether measurements were taken from distinct samples or whether the same sample was measured repeatedly
☐	☒	The statistical test(s) used AND whether they are one- or two-sided<br>*Only common tests should be described solely by name; describe more complex techniques in the Methods section.*
☒	☐	A description of all covariates tested
☐	☒	A description of any assumptions or corrections, such as tests of normality and adjustment for multiple comparisons
☐	☒	A full description of the statistical parameters including central tendency (e.g. means) or other basic estimates (e.g. regression coefficient) AND variation (e.g. standard deviation) or associated estimates of uncertainty (e.g. confidence intervals)
☐	☒	For null hypothesis testing, the test statistic (e.g. *F*, *t*, *r*) with confidence intervals, effect sizes, degrees of freedom and *P* value noted<br>*Give P values as exact values whenever suitable.*
☒	☐	For Bayesian analysis, information on the choice of priors and Markov chain Monte Carlo settings
☒	☐	For hierarchical and complex designs, identification of the appropriate level for tests and full reporting of outcomes
☐	☒	Estimates of effect sizes (e.g. Cohen's *d*, Pearson's *r*), indicating how they were calculated

Our web collection on statistics for biologists contains articles on many of the points above.

Software and code

Policy information about availability of computer code

Data collection	No non-standard software was used for experimental data collection.
Data analysis	FastQC; fastp (v0.22.0); seqkit (v2.10.0); TRANSIT (v3.3.13); BWA (v0.7.12); RStudio (v4.3.2); Trimmomatic (v.0.39); Unicycler; Geneious Prime 2023.0.4 (including MAFFT v7.490); HHpred; Dali server; AlphaFold2 (via ColabFold); PyMOL Molecular Graphics System v3.0.4.<br>The custom R-studio code used in this study is available at Zenodo https://doi.org/10.5281/zenodo.20179451.

For manuscripts utilizing custom algorithms or software that are central to the research but not yet described in published literature, software must be made available to editors and reviewers. We strongly encourage code deposition in a community repository (e.g. GitHub). See the Nature Portfolio guidelines for submitting code & software for further information.

Data

Policy information about availability of data

All manuscripts must include a data availability statement. This statement should provide the following information, where applicable:

- Accession codes, unique identifiers, or web links for publicly available datasets
- A description of any restrictions on data availability
- For clinical datasets or third party data, please ensure that the statement adheres to our policy

All data supporting the findings of this study are available with this paper. Source data from the HIDEN-SEQ experiments are organized by phage in Supplementary Data 4-6, with the corresponding datasets indicated in the respective figure legends. The raw reads of our HIDEN-SEQ experiments have been deposited in the NCBI

Sequence Read Archive (SRA) under BioProject accession number PRJNA1454991. The corresponding SRA run accessions (SRR) are listed in Supplementary Data 4-6. In addition, the raw sequencing data and genome assemblies for the uropathogenic E. coli isolates are available in the European Nucleotide Archive (ENA) under study accession PRJEB111350.
HIDEN-SEQ plasmids are available via Addgene with IDs 257247 (pAH186ColE1_Himar1_T'ase), 257248 (pAH160_Tn_AlcrVIA3_v2), 257249 (pDH160_Tn_AcrVIA1_v2), 257250 (pAH221_LbuCas13a), 257251 (pAH218_LbuCas13a_e), 257252 (pAH221_LseCas13a), and 257253 (pAH218_LseCas13a_e).

The R-studio code used for the gene essentiality analysis of HIDEN-SEQ libraries is available at Zenodo with the digital object identifier https://doi.org/10.5281/zenodo.20179451.

Research involving human participants, their data, or biological material

Policy information about studies with human participants or human data. See also policy information about sex, gender (identity/presentation), and sexual orientation and race, ethnicity and racism.

Reporting on sex and gender	Human participants of all sexes were included; however, sex and gender were not specifically analyzed or reported in this manuscript, as the focus was solely on bacterial and viral findings.
Reporting on race, ethnicity, or other socially relevant groupings	Human participants were included without any race restrictions. No information on race is reported in this study, as only bacterial and viral data were analyzed. Only bacterial strains isolated from human patients were used in this study, no patient data / information of any sort.
Population characteristics	Participants with suspected asymptomatic bacteriuria or urinary tract infections were recruited in accord with a study protocol approved by the responsible ethical commission (see "Ethics oversight"). Population characteristics are not reported.
Recruitment	Participants with suspected asymptomatic bacteriuria or urinary tract infections were recruited in accord with a study protocol approved by the responsible ethical commission (see "Ethics oversight").
Ethics oversight	The clinical work leading to isolation of clinical bacterial strains reported in this manuscript was part of a study approved by the ethical commission of northwest and central Switzerland (EKNZ, approval 2020_02588).

Note that full information on the approval of the study protocol must also be provided in the manuscript.

Field-specific reporting

Please select the one below that is the best fit for your research. If you are not sure, read the appropriate sections before making your selection.

☒ Life sciences ☐ Behavioural & social sciences ☐ Ecological, evolutionary & environmental sciences

For a reference copy of the document with all sections, see nature.com/documents/nr-reporting-summary-flat.pdf

Life sciences study design

All studies must disclose on these points even when the disclosure is negative.

Sample size	Sample sizes were based on common practice for molecular microbiology. For validation experiments, independent biological replicates were performed as indicated in the figure legends. For HIDEN-SEQ experiments, library sizes were determined by the number of independent transposon mutants recovered to achieve sufficient library saturation for genome-wide gene essentiality analysis.
Data exclusions	An initial Bas54 HIDEN-SEQ library with insufficient saturation had initially been shown in an earlier version of this manuscript but was later removed as suggested by reviewers during peer review. A repeat experiment generated a highly saturated library that was used for all downstream analyses. No other data were excluded.
Replication	Independent replicates were performed where appropriate. Validation experiments were successful and yielded similar results across independent biological replicates. Replication details are provided in the figure legends and/or Methods.
Randomization	N/A
Blinding	N/A

Reporting for specific materials, systems and methods

We require information from authors about some types of materials, experimental systems and methods used in many studies. Here, indicate whether each material, system or method listed is relevant to your study. If you are not sure if a list item applies to your research, read the appropriate section before selecting a response.

Materials & experimental systems

n/a	Involved in the study
☒	☐ Antibodies
☒	☐ Eukaryotic cell lines
☒	☐ Palaeontology and archaeology
☒	☐ Animals and other organisms
☐	☒ Clinical data
☒	☐ Dual use research of concern
☒	☐ Plants

Methods

n/a	Involved in the study
☒	☐ ChIP-seq
☒	☐ Flow cytometry
☒	☐ MRI-based neuroimaging

Clinical data

Policy information about clinical studies

All manuscripts should comply with the ICMJE guidelines for publication of clinical research and a completed CONSORT checklist must be included with all submissions.

Clinical trial registration: The clinical work leading to the isolation of bacterial strains reported in this manuscript has been registered on ClinicalTrials.gov: NCT05017766.

Study protocol: A detailed overview of the study design/protocol is provided on ClinicalTrials.gov (identifier NCT05017766).

Data collection: Routine clinical data from patients with suspected asymptomatic bacteriuria or urinary tract infection are collected for this study and encoded accordingly. The non-clinical side cannot trace clinical data back to individual patients. No clinical data were used within this project.

Outcomes: n/a

Plants

Seed stocks: No seed stocks were used in this study.

Novel plant genotypes: No plants were used in this study.

Authentication: No plants were used in this study.
