# Supplementary material for: Systematic mapping of bacteriophage gene essentiality with HIDEN-SEQ
Source: Nat Microbiol. 2026 Sep 25;11(10):2751–63. doi: 10.1038/s41564-026-02455-8 (PMC13716836; doi:10.1038/s41564-026-02455-8)
Supplement: Supplementary file 4 [file 41564_2026_2455_MOESM3_ESM.pdf]

nature portfolio

Peer Review File

Systematic mapping of bacteriophage gene essentiality with HIDEN-SEQ

Corresponding Author: Professor Alexander Harms

Version 0:

Reviewer comments:

Reviewer #1

(Remarks to the Author)
Research on phage-bacteria interactions has driven biotechnological advances and inspired clinical treatments against antibiotic-resistant pathogens. However, a substantial fraction of genes in most phage genomes have unknown functions, limiting our understanding of phage biology. To address this gap, the authors present HIDEN-SEQ, an adaptation of Tn-seq for phages. A key innovation is the use of an anti-CRISPR protein as a selectable marker, which allows for enrichment of transposon mutants on a bacterial host expressing the corresponding CRISPR-Cas system. To benchmark their method, the authors use HIDEN-SEQ to retrieve known essential and conditionally essential genes in the model phage T4. The authors then implement HIDEN-SEQ to map essential genes in phylogenetically distinct phages. Based on these analyses, they identify novel anti-host defense genes in multiple phages, highlighting the utility of the method for biological discovery. A particular strength of the manuscript is the development of a custom gene essentiality classification pipeline to account for the smaller size and lower complexity of phage genomes.

Overall, this manuscript is likely to be of high interest to the microbiology research community. Although two other recent pre-prints describe conceptually similar methodologies as HIDEN-SEQ, differences in the mutagenesis strategy and analytical pipeline of HIDEN-SEQ make it original and complementary to the other methodologies. In addition, all three groups have applied their methods to distinct phages, revealing novel information about their biology. Nevertheless, there are technical and conceptual issues that need to be addressed prior to publication. These issues primarily relate to a lack of validation of essentiality calls, a superficial treatment of potential polar effects, and limited characterization of some of the transposon mutant libraries, as further outlined below.

Major Comments
1. Lack of orthogonal validation of gene essentiality calls
The manuscript contains no direct validation of essentiality calls derived from HIDEN-SEQ, for neither T4, Bas37, nor Bas54. Although overall the pipeline appears to work robustly, orthogonal validation would instill confidence in the generality and accuracy of the method. Such validation would be particularly pertinent i) for the cases in which HIDEN-SEQ yields results that conflict with previous reports, such as for the six T4 genes that appeared essential by HIDEN-SEQ (lines 282-283) but were reported as not essential in previous studies (ref. 10) and ii) or for the differences in essentiality patterns between Bas37 and T4, despite the high degree of relatedness between these phages (Fig. 3A, Ext. Fig. 4D; lines 178-185). Validation could consist of targeting a subset of putative non-essential and essential genes by orthogonal methods, such as genetic knockout, Cas13a inhibition, or anti-sense oligos.

2. Insufficient evaluation of potential polar effects
A related concern is the superficial treatment of polar effects as potential confounders of essentiality calls. Such polar effects (both forward and reverse) are well-characterized confounders of Tn-seq in bacteria. Two recent pre-prints document that polar effects also affect essentiality calls from Tn-seq in phages. One pre-print (https://www.biorxiv.org/content/10.1101/2025.11.23.690004v2) explicitly notes some polar effects. Although the work in the pre-print uses a different transposon (that notably includes a transcriptional terminator, apparently by contrast to the transposon used in this manuscript) and involves a different phage, the precedent suggests that polar effects are important to consider. The second pre-print (https://www.biorxiv.org/content/10.64898/2025.12.19.695335v1) reported that insertions in some genes were enriched in the orientation that allowed the Acr to be transcribed in the same direction as phage genes, implicating polarity. Together, this precedent makes it important to rigorously assess the influence of polar effects on essentiality calls by HIDEN-SEQ.

In this manuscript, the discussion of polarity is largely confined to a statement that “insertions upstream of essential genes were usually well tolerated” (lines 301-303), with the conclusion that polar effects were negligible. However, the presence of insertions upstream of a subset of essential genes does not exclude the possibility of polar effects confounding essentiality calls at other loci. In addition, the HIDEN-SEQ analysis pipeline appears to not consider biases in orientation of insertion. It would increase

confidence in the accuracy of HIDEN-SEQ essentiality calls if the analysis pipeline differentiated insertions by orientation and factored such differences into essentiality calls. More broadly, we recommend that the authors conduct orthogonal validation of essentiality calls for a subset of genes within operons and expand their discussion of polar effects.

3. Characterization of generated transposon mutant libraries
In several instances, it would be helpful for readers to have additional information on the quality of the generated transposon mutant libraries. This information is critical to evaluate how robust and generalizable the mutagenesis pipeline is.

First, the authors do not directly measure how many transposon mutants are retrieved following mutagenesis. This knowledge would be helpful to infer whether all insertion sites in the genome were saturated prior to selection, which is a problem the authors ran into with their first Bas54 transposon library. A direct measurement of the number of mutants for some of their libraries (e.g. through plating of the phage lysate immediately after mutagenesis on permissive/restrictive hosts) would be helpful for readers.

Second, the authors note that escape mutants that do not carry a transposon but become CRISPR-resistant would reduce the efficiency with which transposon mutants are retrieved. They describe in their methods that they performed an experiment to estimate the frequency of escape mutants in their T4 transposon library (lines 537-539). However, they do not show data to support their claim that the frequency of escape mutants is low (lines 545-547). Please show these data.

Third, the authors generated two independent T4 transposon mutant libraries, with the only difference being deletion of rnlA in the host background. How well did essentiality calls by HIDEN-SEQ correlate between these datasets? This information could instill further confidence into the accuracy of the results.

Fourth, as part of this work the authors used HIDEN-SEQ to generate transposon mutant libraries for Bas37 and Bas54 but show only limited data that support the quality of the libraries. Do the authors have sequencing data from the transposon mutant libraries prior to selection, similar to T4 (Ext. Fig. 2A-B)? These data would increase confidence that the authors saturated mutagenesis of the Bas37 and Bas54 genomes, and that their pre-selection libraries were sufficiently complex in terms of insertions represented.

Minor Comments
General text
1. Several statements in the manuscript are inappropriately hyperbolic, such as the multiple mentions of the “unprecedented depth and resolution” of HIDEN-SEQ and referencing the “critical breakthrough” of HIDEN-SEQ. Similarly, the authors claim that HIDEN-SEQ has “near base-pair resolution”, but HIDEN-SEQ relies on mutagenesis via a mariner transposon that inserts specifically at TA dinucleotide sites, or roughly once every 8 base pairs at average GC content; the transposon itself causes a mutation that is much greater than a single base change; and the insertion sites are not distributed evenly through every phage genome. Describing the resolution of HIDEN-SEQ as “near base-pair” is misleading. Please modify the wording.
2. The authors make a broad claim about the utility of HIDEN-SEQ for identification of conditionally essential genes. Such identification does suffer from the issue that the transposon mutant library must be generated in a bacterial host, such that any genes that are conditionally essential in that host will become depleted in insertions during library generation. Thus, there is strong directionality in conditional essentiality; defining conditional essentiality for genes that are essential in the library generation host (a large number of genes) but not in other hosts is difficult by this method. The authors get around this issue by constructing a transposon mutant library of T4 in a ΔrnlA host to demonstrate that dmd is conditionally essential in wild-type E. coli K-12. This approach is confirmatory, in that it requires prior knowledge of a potential conditional essentiality. As written, it is not a scalable tool for discovery. We recommend including an explicit discussion of limitations of conditional essentiality analysis with HIDEN-SEQ in the main text.
3. Given that two other recent pre-prints describe methodologies that are conceptually similar to HIDEN-SEQ, it would be beneficial for the authors to include a brief comparison in the discussion, highlight aspects of their own methodology that are unique, and note overlapping or conflicting findings from the studies collectively.

Abstract and Introduction
4. It would be appropriate to introduce the HIDEN-SEQ acronym upon first mention in the abstract.
5. It would be helpful for readers to briefly note in the introduction the shortcomings of existing methods for mutagenesis of phage genomes, as a motivation for the development of HIDEN-SEQ. The authors have such statements in the discussion.

All Figures
6. For figures showing insertion site sequencing read counts across the genome, the authors cap the axis at 150 counts. Can the authors explain this choice? Is there any additional information encoded in higher counts?
7. Could the authors show quantifications of plaque-forming units per mL for their spot assays, for example as bar graphs, to make these results easier to interpret?

Figure 1
8. The authors mention in their discussion (lines 282-283) that T4 genes inh, hoc, segE, uvsW, segC, and wac appeared essential in their HIDEN-SEQ screen but were reported as not essential in previous studies (ref. 10). It would be helpful if this information was moved up in the main text (lines 98-100), so that readers are aware of these discrepancies when assessing the data in Fig. 1B.
9. In Fig. 1C, the authors show validation of the domain-specific essentiality of rnlA, but do not describe this experiment in the main text.
10. For the rnlA finding, it would be helpful to provide some context regarding the known activity of rnlA and the known roles of the N-terminal and C-terminal domains.
11. Line 107: Noun missing after “C-terminal” (or change to “C-terminus”).

Figure 2
12. The specificity with which the authors identified dmd as conditionally essential in wild-type K-12 is nicely documented through the volcano plot in Ext. Fig. 3B. The authors note that they also identified conditionally essential genes identified through infection of hosts that express RexAB, CBASS, DarTG1, and DarTG2. Please provide similar volcano plots to give readers a sense of the sensitivity and specificity of these conditional essentiality analyses.
13. For Fig. 2G, it is unclear how "probability = 99.71%, E-value = 2.6×10^{-14}" was calculated based on the description of the structural analysis in the caption and methods.
Figure 4
14. The authors state that the E. coli clinical isolates for their work in Figure 4 were obtained through the Swiss NCCR AntiResist consortium, with more information to be published elsewhere (lines 210-211). Are there any existing publications or pre-prints that could help the readers understand how these isolates were obtained?
15. Fig. 4 is difficult to follow while reading the main text, because the data for each finding is split across multiple panels. Please consider rearranging the volcano plot(s) and data from phage dilution assays such that they are paired by finding.
16. The authors claim that bas37_0154 is involved in evading the Mokosh type I defense system of E. coli strain CFE331, supported by the observation that bas37_0154 knockout inhibits Bas37 propagation in CFE331 (Fig. 4B). Can the authors explain why insertions in bas37_0154 were not significantly depleted when HIDEN-SEQ was applied to CFE331 (in particular the q-value appears low; Fig. 4A)?
17. For Fig. 4E, the phage dilution assay with K-12 ΔRM expressing the CFE311 Gao_Tmn_3 defense system is not discussed in the main text.
18. Ext. Fig. 8F caption: "N-terminal" should read "N-terminus".

Data Availability and Code
In general, raw sequencing data for this work should be uploaded to a publicly accessible database.

The authors cite all of the tools they used for read processing, alignment to transposons and phage genomes, and read subsampling (ref. 62-65). For essentiality analysis, the authors state that their custom code is available on Zenodo, with a digital object identifier that is yet to be released (lines 736-737). For conditional essentiality analysis, the authors cite the well-maintained Tn-seq analysis tool TRANSIT (ref. 63). They provide sufficient information on the parameters used for each of their tools that the analysis could theoretically be replicated. However, since we do not have access to the raw sequencing data, we were unable to reproduce the analyses.

Reviewer #2

(Remarks to the Author)
Humolli et al. developed a transposon mutagenesis screen for phages in E. coli. In the first step, a marina transposon system was established with an anti-Cas13a factor that can insert randomly at TA sites in the phage genome, the resulting phages are then selected and enriched in a second step via a host where only phages with the transposon are able to inhibit the Cas13a system that targets an essential late phage transcript. The authors validate the approach for T4 phage and show that insertions can discriminate also among defined domain essentiality at sub-gene resolution for one gene rnlA. In the next step, this technology was used to test conditionally essential genes connected to anti-defence functions that were validated by expression or deletion of anti-phage defence genes. The authors identified a new anti-defence gene tk.4 against DarTG2 anti-phage defence, and others. In addition, the conditional essentiality was tested in minimal media and resulted in the essentiality of the valyl-tRNA synthetase. The approach was also further validated through application on a T4 relative phage with highly similar outcome that further supports the reproducibility. In addition, the approach was applied to a V5-like phage. Then the authors turned to screen different hosts, and realized that for the V5-like phage that encodes multiple tail fibers these appeared to be responsible for fitness in specific clinical isolates. The authors point also out an intergenic region that appears essential and remains uncharacterised among many other factors, mentioning that non-coding regions can be also targeted and reveal essentiality. Screening clinical isolates with the phage library in combination with the prediction of anti-phage defence genes, allowed to authors to pinpoint a hand full of interactions with phage genes that can negate anti-phage defence systems.
The study utilizes latest available technologies and merges them into a novel system for phage research. The authors came up with a magnitude of blueprints how to make use of the phage transposon library in the context of conditional essentiality linked to anti-defence genes, nutrient availability, and uncharacterised regions. To put this into perspective, this represents one of the very first studies that makes use of transposon mutagenesis in phages coupling this to sequencing-based readouts. This study applies it e.g. to a well characterized T4 phage and shows many novel insights also highlighting the still large discovery space in a highly studied phage. With this technology in hand, the study of Escherichia and other species infecting DNA-phages, will accelerate and allow to map the host-phage interface in unpreceded ways. As exemplified, this study will also guide the discovery of hundreds of anti-defence genes that could be utilised for tailored phage therapy. There remain several major points to address to improve the manuscript.

Major comments

Claim: Portability beyond model phages
The authors illustrate impressively the applicability in T4, a closely related phage, and a V5-like phage. Still, these phages are T4/V5-like DNA-phages, that represents only two out of 34+ taxonomic families from the currently available BASEL phage collection that was also established by the Harms lab. In general, the system requires genetics in the host, transposon integration, and selection. The study would obviously benefit from including other taxonomic families, e.g. the authors included two different Cas13a systems, of which one would very likely work in Pseudomonas, too, as previously used for selection, that could be included or discussed. More important, there are additional challenges on the phage side, that should be covered in a systematic way to solidify the claim to be applicable in many phage-host pairs or to make the limitations clear.

(1) One requirement for the system to work is that the anti-CRISPR protein is expressed. In the current setting it is expressed from a host-like promoter by the host RNAP soon after infection. Does this promoter work also in other species, like Pseudomonas? Some evidence or references should be given, and explained that promoters may require adjustments for other species.

(2) Does the system work with phages like T5 and T7, that do shut down the host transcription that is required for the expression of the anti-CRISPR protein, or does it in this case require an early phage-derived promoter?

(3) Are there limitations related to the partial injection of e.g. the T5 genome? Would the selection step work still with transposon insertions in the complete genome?

(4) Some phages degrade the host genome rapidly, hence the transposon template should get degraded, too. Are there any limitations with the current system related to integration efficiency, e.g. in e.g. T5.

Claim: Near-base-pair resolution.
(1) The authors should take the coverage into account. What coverage would be required for a base-pair resolution claim, usually 5x coverage per base is considered, this is for T4 with 170 kbp about 800k insertions. But, Himar1 insert only into TA-sites, hence this is 14,474 TA sites x 5 is about 75k. How many transposition events were actually recorded? The method section gives an estimation that is purely based on theoretical assumptions. Which MOI was used, it is not stated in the method section? How many replication rounds were possible until the pre-selection library? The T4 library before selection was sequenced, this should be used to estimate the saturation quantitatively by comparing the number of unique insertions related to all predicted TA-sites and put into perspective to the number of events.
(2) In addition, the transposon insertions in the phage library before selection, can be quantified with quantitative PCR to give precise estimation about the integrated transposons in the phage particles before selection, and the number of phages is estimatable by a reference experiment, so quantitative numbers could be returned. This is essential to determine the coverage. It is also technologically important, as it provides the future users with a broadly accessible way to estimate the saturation of the library prior sequencing with defined primers for the transposon. Of note, the distribution is anyway not uniform, e.g. genes 20-23 show less insertions. If the coverage does not reach the initially mentioned number, the claim about base-pair resolution should be rephrased to sub-gene resolution, as this is sufficiently accurate for all claims, and well validated.

Claim: Mapping of conditional essentiality
(1) How are the insertions normalized, e.g. in T4 between 0-10,000 kbp there are only a few insertions before selection in e.g. gene 20, 22, still this is claimed to be essential, do these genes meet the criterion stated in the methods. In this respect showing the distribution of TA sites along the genome, as done in Fig. 2 for genomic loci, would be informative, this can be also done on a sliding window, as it would get otherwise very detailed. It is a common pitfall with transposon mutagenesis to interpret low-insertion counts as essentiality. The authors determined the transposon sites before selection but how is this considered further for the evaluation of essentiality in the analysis? In general, this can be used to add confidence values. This is even more important for the Bas54 experiment where the coverage was lower. This should be commented in the text, re-evaluated based on the library before selection and continuous colouring should be considered.

(2) What if a transposon inserts in reverse to the sense strand, then there is an antisense transcript transcribed that could interfere with close transcripts from the other strand through base-pairing that encode possibly for essential genes. In essence, this could lead to an overestimation of essential genes. The authors should investigate this by comparing the strand orientation of insertions, the impact of the selection step on the ratio, and discuss it.

Minor comments

In general, the manuscript should provide more context and details about the method starting from expression, selection mechanism of Cas13a, and so on, this is important and can be edited in the manuscript.

Please consider also to switch Fig. 2 and 3, as it goes a back and forth between phage essentiality screening and anti-defence/clinical isolate screening.

Ext. Data. Fig. 2b,c and other similar panels, please comment on the following observation: If before selection only about 5 genes had insertions in all TA-sites in panel b, and then there is selection and expansion performed, how can it be that then in panel c 150 genes have insertions in all TA-sites, where do these additional ones come from during the selection if these were not initially there?

A common reason for lack of insertions is blocking of loci by DNA-binding proteins, is there anything described for the region 0-10k in T4. The authors could dwell on this in the discussion to make also the reader aware that low-insertion counts can be possibly interpreted.

The prominently positioned rnlA example is intriguing to show sub-gene insertion effects, but ultimately the transposon breaks the open reading frame coding for the C-terminal domain, that was also ensured in general through stop codons in all three frames in the transposon. Would this work also for the N-terminal domains only? Would it require transcription/translation initiation sites in the transposon? This should be clarified in the text as a limitation related to sub-gene resolution claims.

Other phages do encode or inject RNAPs to transcribe their genome from distinct promoters where still the host RNAP could work. But, in cases like nucleus-forming phages or shutdown of the host-RNAP the anti-CRISPR protein must be transcribed from phage promoters. This scenario requires to add phage-own promoters for transposon expression, please discuss this in the

manuscript.

Again nucleus-forming phages, require the transposase to be imported into the phage nucleus to integrate into the amplified phage genome, so here fusion of the transposase to a localisation protein would be required, please discuss this in the manuscript.

Do DNA modifications impair the transposon insertion, usually cytosine modifications are reported for phages, the here selected system does insert at TA-sites, so no conflict, but this should be discussed as a potential limitation.

In general, a more continuous colour coding for essentiality would be interesting to see, as there are sometimes effects but these are omitted, e.g. with vs.1 in Fig. 2.

Please comment also on the two phenotypes related to anti-defence gene knockouts, why in some cases there are no transposons detected and in others there are still many detected at a lower rate, can this be related to the efficiency of the system and the number of selection rounds undertaken. Can this be shown based on a quantitative PCR readout of the locus that probes for insertions and no-insertions, where after more rounds there is an enrichment for the wt? It would be very helpful for the community, to show how defined loci can be probed without running a full sequencing experiment that would also allow to test the dynamics of defined systems and their essentiality.

Please discuss in the main text the impact of transposon insertions on transcriptional units and termination events, or if these can be excluded.

As many phages encode for anti-CRISPR proteins, this should be also discussed as a limitation.

p. 4 l. 55: Please give more information about the approach of these methods and the clear limitations. It will also make clear where the advantages are related to transposon-based approach.

p. 4 l. 59: It is not always a phenotypic selection required.

p. 4 l. 62: An efficient selection marker by an essential phage gene was reported, it should be rather universal markers.

p. 5 l. 78 and l. 80: The LseCas13a targets RNA not ultimately the gene, but by transcript please correct this as if a gene is not transcribed it will not be targeted. In addition, it would be good to mention that mRNA modifications are not really existing in phage transcripts, this adds to the point that the method is universal at the selection.

p. 5 l. 79: The reader may ask, why the targeting of a single mRNA from a phage (even possibly a non-essential one), is leading to a selection, please comment on the working mechanism of selection.

p. 5 l. 86f.: There are some intermediate steps to be considered that is important to be clarified here. It would be important to mention the promoter that was used here with the acrVIA1 gene and to comment that this must be expressed early in infection to allow for inactivation by Cas13a that may likely target later genes. In this context, the current version of the manuscript does not mention which transcript is targeted by Cas13a in the main text, please add this in this paragraph, and explain also why targeting a major capsid protein is an important choice for the method.

p. 6 l. 102f. add reference to Fig. 1b again.

Discussion: Please comment on the transferability to other species, the LseCas12a can be very likely transferred to Pseudomonas phages, as this system was used previously in Pseudomonas (PMID: 36316452).

Fig. 1b: I could not find a single gene with reduced fitness colouring, is this true, or is there a lack in colouring?

p. 28: please state the promoters used for the acr

p. 29, l. 532: Is here “clones” correct or should it be plaques.

p. 30: please state at which MOI the cells were infected with T4 to generate the library, also for the other phages.

Fig. 1a, copy numbers of the plasmids should be indicated, and in the selection step the plasmid is missing. The blue transposase indication vs regular black genome in the bottom line will never be visible in b/w printouts, please mark it more prominently. The blue lines indicating transposon insertions show gaps between the genes, but actually, the intergenic regions are much more prone towards transposon insertions. The transposon insertion is not well depicted as there is no wt phage genome illustrated in the upper left part. The mechanism of inhibition by anti-CRISPR protein does not become evident, e.g. if the yellow transcript part (target) is transcribed at the same time as the blue (acr) the Cas13a will get activated, time-dependent events should be depicted clearly.

Extended Data Fig. 2B and similar other panels, essential and non-essential labels do not play a role here, and should be removed.

Reviewer #3

(Remarks to the Author)
All my comments have been integrated into the other reviewer's submission.

Decision Letter:

24th February 2026

*Please ensure you delete the link to your author homepage in this e-mail if you wish to forward it to your co-authors.

Dear Alexander,

Thank you for your patience while your manuscript "Systematic mapping of bacteriophage gene essentiality with HIDEN-SEQ" was under peer-review at Nature Microbiology. It has now been seen by 3 referees, whose expertise and comments you will find at the end of this email. Although they find your work of some potential interest, they have raised a number of concerns that will need to be addressed before we can consider publication of the work in Nature Microbiology.

In particular, the referees independently brought up three major concerns. First, some issues with the lack of validation of essentiality, which will require additional analysis it seems. Second, there were also overlapping concerns about polar effects as potential confounders to essentiality, and this will also require some assessment. Finally, the referees also agree that some claims are overblown, and these will need to be toned down a bit in line with the conclusions as supported by the data.

Should further experimental data allow you to address these criticisms, we would be happy to look at a revised manuscript.

We are committed to providing a fair and constructive peer-review process. Please do not hesitate to contact us if there are specific requests from the reviewers that you believe are technically impossible or unlikely to yield a meaningful outcome.

We strongly support public availability of data. Please place the data used in your paper into a public data repository, if one exists, or alternatively, present the data as Source Data or Supplementary Information. If data can only be shared on request, please explain why in your Data Availability Statement, and also in the correspondence with your editor. For some data types, deposition in a public repository is mandatory - more information on our data deposition policies and available repositories can be found at https://www.nature.com/nature-research/editorial-policies/reporting-standards#availability-of-data.

Please include a data availability statement as a separate section after Methods but before references, under the heading "Data Availability". This section should inform readers about the availability of the data used to support the conclusions of your study. This information includes accession codes to public repositories (data banks for protein, DNA or RNA sequences, microarray, proteomics data etc…), references to source data published alongside the paper, unique identifiers such as URLs to data repository entries, or data set DOIs, and any other statement about data availability. At a minimum, you should include the following statement: “The data that support the findings of this study are available from the corresponding author upon request”, mentioning any restrictions on availability. If DOIs are provided, we also strongly encourage including these in the Reference list (authors, title, publisher (repository name), identifier, year). For more guidance on how to write this section please see:
http://www.nature.com/authors/policies/data/data-availability-statements-data-citations.pdf

If revising your manuscript:

* Include a “Response to referees” document detailing, point-by-point, how you addressed each referee comment. If no action was taken to address a point, you must provide a compelling argument. This response will be sent back to the referees along with the revised manuscript.

* If you have not done so already we suggest that you begin to revise your manuscript so that it conforms to our Article format instructions at http://www.nature.com/nmicrobiol/info/final-submission. Refer also to any guidelines provided in this letter.

* Include a revised version of any required reporting checklist. It will be available to referees (and, potentially, statisticians) to aid in their evaluation if the manuscript goes back for peer review. A revised checklist is essential for re-review of the paper.

When submitting the revised version of your manuscript, please pay close attention to our href="https://www.nature.com/nature-portfolio/editorial-policies/image-integrity">Digital Image Integrity Guidelines. and to the following points below:

-- that unprocessed scans are clearly labelled and match the gels and western blots presented in figures.
-- that control panels for gels and western blots are appropriately described as loading on sample processing controls
-- all images in the paper are checked for duplication of panels and for splicing of gel lanes.

EXTENDED DATA FIGURES

When re-submitting your manuscript, please ensure that any supplementary figures and tables that are crucial to the manuscript’s conclusions are converted into Extended Data figures and tables to increase visibility of these data. Extended Data figures and tables are online-only (present in the online PDF and full-text HTML versions of the paper), peer-reviewed display items that provide essential background to the article but are not included in the main article due to space constraints. A maximum of ten Extended Data display items (figures and tables) is permitted.

Finally, please ensure that you retain unprocessed data and metadata files after publication, ideally archiving data in perpetuity, as these may be requested during the peer review and production process or after publication if any issues arise.

Please use the link below to submit a revised paper:

Link Redacted

Note: This url links to your confidential homepage and associated information about manuscripts you may have submitted or be reviewing for us. If you wish to forward this e-mail to co-authors, please delete this link to your homepage first.

Nature Microbiology is committed to improving transparency in authorship. As part of our efforts in this direction, we are now requesting that all authors identified as 'corresponding author' on published papers create and link their Open Researcher and Contributor Identifier (ORCID) with their account on the Manuscript Tracking System (MTS), prior to acceptance. This applies to primary research papers only. ORCID helps the scientific community achieve unambiguous attribution of all scholarly contributions. You can create and link your ORCID from the home page of the MTS by clicking on 'Modify my Springer Nature account'. For more information please visit please visit www.springernature.com/orcid.

If you wish to submit a suitably revised manuscript we would hope to receive it within 6 months. If you cannot send it within this time, please let us know. We will be happy to consider your revision, even if a similar study has been accepted for publication at Nature Microbiology or published elsewhere (up to a maximum of 6 months).

In the meantime we hope that you find our referees' comments helpful.

Yours sincerely,

****

Reviewer Expertise:

Referee #1: genetics and genomics, CRISPR approaches, phage defense
Referee #2: functional genomics, phage biology
Referee #3: co-reviewer with R1

Reviewer Comments:

Reviewer #1 (Remarks to the Author):

Research on phage-bacteria interactions has driven biotechnological advances and inspired clinical treatments against antibiotic-resistant pathogens. However, a substantial fraction of genes in most phage genomes have unknown functions, limiting our understanding of phage biology. To address this gap, the authors present HIDEN-SEQ, an adaptation of Tn-seq for phages. A key innovation is the use of an anti-CRISPR protein as a selectable marker, which allows for enrichment of transposon mutants on a bacterial host expressing the corresponding CRISPR-Cas system. To benchmark their method, the authors use HIDEN-SEQ to retrieve known essential and conditionally essential genes in the model phage T4. The authors then implement HIDEN-SEQ to map essential genes in phylogenetically distinct phages. Based on these analyses, they identify novel anti-host defense genes in multiple phages, highlighting the utility of the method for biological discovery. A particular strength of the manuscript is the development of a custom gene essentiality classification pipeline to account for the smaller size and lower complexity of phage genomes.

Overall, this manuscript is likely to be of high interest to the microbiology research community. Although two other recent pre-prints describe conceptually similar methodologies as HIDEN-SEQ, differences in the mutagenesis strategy and analytical pipeline of HIDEN-SEQ make it original and complementary to the other methodologies. In addition, all three groups have applied their methods to distinct phages, revealing novel information about their biology. Nevertheless, there are technical and conceptual issues that need to be addressed prior to publication. These issues primarily relate to a lack of validation of essentiality calls, a superficial treatment of potential polar effects, and limited characterization of some of the transposon mutant libraries, as further outlined below.

Major Comments
1. Lack of orthogonal validation of gene essentiality calls
The manuscript contains no direct validation of essentiality calls derived from HIDEN-SEQ, for neither T4, Bas37, nor Bas54. Although overall the pipeline appears to work robustly, orthogonal validation would instill confidence in the generality and accuracy of the method. Such validation would be particularly pertinent i) for the cases in which HIDEN-SEQ yields results that conflict with previous reports, such as for the six T4 genes that appeared essential by HIDEN-SEQ (lines 282-283) but were reported as not essential in previous studies (ref. 10) and ii) or for the differences in essentiality patterns between Bas37 and T4, despite the high degree of relatedness between these phages (Fig. 3A, Ext. Fig. 4D; lines 178-185). Validation could consist of targeting a subset of putative non-essential and essential genes by orthogonal methods, such as genetic knockout, Cas13a inhibition, or anti-sense oligos.

2. Insufficient evaluation of potential polar effects

A related concern is the superficial treatment of polar effects as potential confounders of essentiality calls. Such polar effects (both forward and reverse) are well-characterized confounders of Tn-seq in bacteria. Two recent pre-prints document that polar effects also affect essentiality calls from Tn-seq in phages. One pre-print (https://www.biorxiv.org/content/10.1101/2025.11.23.690004v2) explicitly notes some polar effects. Although the work in the pre-print uses a different transposon (that notably includes a transcriptional terminator, apparently by contrast to the transposon used in this manuscript) and involves a different phage, the precedent suggests that polar effects are important to consider. The second pre-print (https://www.biorxiv.org/content/10.64898/2025.12.19.695335v1) reported that insertions in some genes were enriched in the orientation that allowed the Acr to be transcribed in the same direction as phage genes, implicating polarity. Together, this precedent makes it important to rigorously assess the influence of polar effects on essentiality calls by HIDEN-SEQ.

In this manuscript, the discussion of polarity is largely confined to a statement that "insertions upstream of essential genes were usually well tolerated" (lines 301-303), with the conclusion that polar effects were negligible. However, the presence of insertions upstream of a subset of essential genes does not exclude the possibility of polar effects confounding essentiality calls at other loci. In addition, the HIDEN-SEQ analysis pipeline appears to not consider biases in orientation of insertion. It would increase confidence in the accuracy of HIDEN-SEQ essentiality calls if the analysis pipeline differentiated insertions by orientation and factored such differences into essentiality calls. More broadly, we recommend that the authors conduct orthogonal validation of essentiality calls for a subset of genes within operons and expand their discussion of polar effects.

3. Characterization of generated transposon mutant libraries
In several instances, it would be helpful for readers to have additional information on the quality of the generated transposon mutant libraries. This information is critical to evaluate how robust and generalizable the mutagenesis pipeline is.

First, the authors do not directly measure how many transposon mutants are retrieved following mutagenesis. This knowledge would be helpful to infer whether all insertion sites in the genome were saturated prior to selection, which is a problem the authors ran into with their first Bas54 transposon library. A direct measurement of the number of mutants for some of their libraries (e.g. through plating of the phage lysate immediately after mutagenesis on permissive/restrictive hosts) would be helpful for readers.

Second, the authors note that escape mutants that do not carry a transposon but become CRISPR-resistant would reduce the efficiency with which transposon mutants are retrieved. They describe in their methods that they performed an experiment to estimate the frequency of escape mutants in their T4 transposon library (lines 537-539). However, they do not show data to support their claim that the frequency of escape mutants is low (lines 545-547). Please show these data.

Third, the authors generated two independent T4 transposon mutant libraries, with the only difference being deletion of rnlA in the host background. How well did essentiality calls by HIDEN-SEQ correlate between these datasets? This information could instill further confidence into the accuracy of the results.

Fourth, as part of this work the authors used HIDEN-SEQ to generate transposon mutant libraries for Bas37 and Bas54 but show only limited data that support the quality of the libraries. Do the authors have sequencing data from the transposon mutant libraries prior to selection, similar to T4 (Ext. Fig. 2A-B)? These data would increase confidence that the authors saturated mutagenesis of the Bas37 and Bas54 genomes, and that their pre-selection libraries were sufficiently complex in terms of insertions represented.

Minor Comments
General text
1. Several statements in the manuscript are inappropriately hyperbolic, such as the multiple mentions of the "unprecedented depth and resolution" of HIDEN-SEQ and referencing the "critical breakthrough" of HIDEN-SEQ. Similarly, the authors claim that HIDEN-SEQ has "near base-pair resolution", but HIDEN-SEQ relies on mutagenesis via a mariner transposon that inserts specifically at TA dinucleotide sites, or roughly once every 8 base pairs at average GC content; the transposon itself causes a mutation that is much greater than a single base change; and the insertion sites are not distributed evenly through every phage genome. Describing the resolution of HIDEN-SEQ as "near base-pair" is misleading. Please modify the wording.
2. The authors make a broad claim about the utility of HIDEN-SEQ for identification of conditionally essential genes. Such identification does suffer from the issue that the transposon mutant library must be generated in a bacterial host, such that any genes that are conditionally essential in that host will become depleted in insertions during library generation. Thus, there is strong directionality in conditional essentiality; defining conditional essentiality for genes that are essential in the library generation host (a large number of genes) but not in other hosts is difficult by this method. The authors get around this issue by constructing a transposon mutant library of T4 in a ΔrnlA host to demonstrate that dmd is conditionally essential in wild-type E. coli K-12. This approach is confirmatory, in that it requires prior knowledge of a potential conditional essentiality. As written, it is not a scalable tool for discovery. We recommend including an explicit discussion of limitations of conditional essentiality analysis with HIDEN-SEQ in the main text.
3. Given that two other recent pre-prints describe methodologies that are conceptually similar to HIDEN-SEQ, it would be beneficial for the authors to include a brief comparison in the discussion, highlight aspects of their own methodology that are unique, and note overlapping or conflicting findings from the studies collectively.

Abstract and Introduction
4. It would be appropriate to introduce the HIDEN-SEQ acronym upon first mention in the abstract.
5. It would be helpful for readers to briefly note in the introduction the shortcomings of existing methods for mutagenesis of phage genomes, as a motivation for the development of HIDEN-SEQ. The authors have such statements in the discussion.

All Figures

6. For figures showing insertion site sequencing read counts across the genome, the authors cap the axis at 150 counts. Can the authors explain this choice? Is there any additional information encoded in higher counts?
7. Could the authors show quantifications of plaque-forming units per mL for their spot assays, for example as bar graphs, to make these results easier to interpret?

Figure 1
8. The authors mention in their discussion (lines 282-283) that T4 genes inh, hoc, segE, uvsW, segC, and wac appeared essential in their HIDEN-SEQ screen but were reported as not essential in previous studies (ref. 10). It would be helpful if this information was moved up in the main text (lines 98-100), so that readers are aware of these discrepancies when assessing the data in Fig. 1B.
9. In Fig. 1C, the authors show validation of the domain-specific essentiality of rnlA, but do not describe this experiment in the main text.
10. For the rnlA finding, it would be helpful to provide some context regarding the known activity of rnlA and the known roles of the N-terminal and C-terminal domains.
11. Line 107: Noun missing after “C-terminal” (or change to “C-terminus”).

Figure 2
12. The specificity with which the authors identified dmd as conditionally essential in wild-type K-12 is nicely documented through the volcano plot in Ext. Fig. 3B. The authors note that they also identified conditionally essential genes identified through infection of hosts that express RexAB, CBASS, DarTG1, and DarTG2. Please provide similar volcano plots to give readers a sense of the sensitivity and specificity of these conditional essentiality analyses.
13. For Fig. 2G, it is unclear how “probability = 99.71%, E-value = 2.6×10^{-14}” was calculated based on the description of the structural analysis in the caption and methods.
Figure 4
14. The authors state that the E. coli clinical isolates for their work in Figure 4 were obtained through the Swiss NCCR AntiResist consortium, with more information to be published elsewhere (lines 210-211). Are there any existing publications or pre-prints that could help the readers understand how these isolates were obtained?
15. Fig. 4 is difficult to follow while reading the main text, because the data for each finding is split across multiple panels. Please consider rearranging the volcano plot(s) and data from phage dilution assays such that they are paired by finding.
16. The authors claim that bas37_0154 is involved in evading the Mokosh type I defense system of E. coli strain CFE331, supported by the observation that bas37_0154 knockout inhibits Bas37 propagation in CFE331 (Fig. 4B). Can the authors explain why insertions in bas37_0154 were not significantly depleted when HIDEN-SEQ was applied to CFE331 (in particular the q-value appears low; Fig. 4A)?
17. For Fig. 4E, the phage dilution assay with K-12 ΔRM expressing the CFE311 Gao_Tmn_3 defense system is not discussed in the main text.
18. Ext. Fig. 8F caption: “N-terminal” should read “N-terminus”.

Data Availability and Code
In general, raw sequencing data for this work should be uploaded to a publicly accessible database.

The authors cite all of the tools they used for read processing, alignment to transposons and phage genomes, and read subsampling (ref. 62-65). For essentiality analysis, the authors state that their custom code is available on Zenodo, with a digital object identifier that is yet to be released (lines 736-737). For conditional essentiality analysis, the authors cite the well-maintained Tn-seq analysis tool TRANSIT (ref. 63). They provide sufficient information on the parameters used for each of their tools that the analysis could theoretically be replicated. However, since we do not have access to the raw sequencing data, we were unable to reproduce the analyses.

Reviewer #2 (Remarks to the Author):

Humolli et al. developed a transposon mutagenesis screen for phages in E. coli. In the first step, a marina transposon system was established with an anti-Cas13a factor that can insert randomly at TA sites in the phage genome, the resulting phages are then selected and enriched in a second step via a host where only phages with the transposon are able to inhibit the Cas13a system that targets an essential late phage transcript. The authors validate the approach for T4 phage and show that insertions can discriminate also among defined domain essentiality at sub-gene resolution for one gene rnlA. In the next step, this technology was used to test conditionally essential genes connected to anti-defence functions that were validated by expression or deletion of anti-phage defence genes. The authors identified a new anti-defence gene tk.4 against DarTG2 anti-phage defence, and others. In addition, the conditional essentiality was tested in minimal media and resulted in the essentiality of the valyl-tRNA synthetase. The approach was also further validated through application on a T4 relative phage with highly similar outcome that further supports the reproducibility. In addition, the approach was applied to a V5-like phage. Then the authors turned to screen different hosts, and realized that for the V5-like phage that encodes multiple tail fibers these appeared to be responsible for fitness in specific clinical isolates. The authors point also out an intergenic region that appears essential and remains uncharacterised among many other factors, mentioning that non-coding regions can be also targeted and reveal essentiality. Screening clinical isolates with the phage library in combination with the prediction of anti-phage defence genes, allowed to authors to pinpoint a hand full of interactions with phage genes that can negate anti-phage defence systems.
The study utilizes latest available technologies and merges them into a novel system for phage research. The authors came up with a magnitude of blueprints how to make use of the phage transposon library in the context of conditional essentiality linked to anti-defence genes, nutrient availability, and uncharacterised regions. To put this into perspective, this represents one of the very first studies that makes use of transposon mutagenesis in phages coupling this to sequencing-based readouts. This study applies it e.g. to a well characterized T4 phage and shows many novel insights also highlighting the still large discovery space

in a highly studied phage. With this technology in hand, the study of Escherichia and other species infecting DNA-phages, will accelerate and allow to map the host-phage interface in unpreceded ways. As exemplified, this study will also guide the discovery of hundreds of anti-defence genes that could be utilised for tailored phage therapy. There remain several major points to address to improve the manuscript.

Major comments

Claim: Portability beyond model phages
The authors illustrate impressively the applicability in T4, a closely related phage, and a V5-like phage. Still, these phages are T4/V5-like DNA-phages, that represents only two out of 34+ taxonomic families from the currently available BASEL phage collection that was also established by the Harms lab. In general, the system requires genetics in the host, transposon integration, and selection. The study would obviously benefit from including other taxonomic families, e.g. the authors included two different Cas13a systems, of which one would very likely work in Pseudomonas, too, as previously used for selection, that could be included or discussed. More important, there are additional challenges on the phage side, that should be covered in a systematic way to solidify the claim to be applicable in many phage-host pairs or to make the limitations clear.
(1) One requirement for the system to work is that the anti-CRISPR protein is expressed. In the current setting it is expressed from a host-like promoter by the host RNAP soon after infection. Does this promoter work also in other species, like Pseudomonas? Some evidence or references should be given, and explained that promoters may require adjustments for other species.

(2) Does the system work with phages like T5 and T7, that do shut down the host transcription that is required for the expression of the anti-CRISPR protein, or does it in this case require an early phage-derived promoter?

(3) Are there limitations related to the partial injection of e.g. the T5 genome? Would the selection step work still with transposon insertions in the complete genome?

(4) Some phages degrade the host genome rapidly, hence the transposon template should get degraded, too. Are there any limitations with the current system related to integration efficiency, e.g. in e.g. T5.

Claim: Near-base-pair resolution.
(1) The authors should take the coverage into account. What coverage would be required for a base-pair resolution claim, usually 5x coverage per base is considered, this is for T4 with 170 kbp about 800k insertions. But, Himar1 insert only into TA-sites, hence this is 14,474 TA sites x 5 is about 75k. How many transposition events were actually recorded? The method section gives an estimation that is purely based on theoretical assumptions. Which MOI was used, it is not stated in the method section? How many replication rounds were possible until the pre-selection library? The T4 library before selection was sequenced, this should be used to estimate the saturation quantitatively by comparing the number of unique insertions related to all predicted TA-sites and put into perspective to the number of events.
(2) In addition, the transposon insertions in the phage library before selection, can be quantified with quantitative PCR to give precise estimation about the integrated transposons in the phage particles before selection, and the number of phages is estimatable by a reference experiment, so quantitative numbers could be returned. This is essential to determine the coverage. It is also technologically important, as it provides the future users with a broadly accessible way to estimate the saturation of the library prior sequencing with defined primers for the transposon. Of note, the distribution is anyway not uniform, e.g. genes 20-23 show less insertions. If the coverage does not reach the initially mentioned number, the claim about base-pair resolution should be rephrased to sub-gene resolution, as this is sufficiently accurate for all claims, and well validated.

Claim: Mapping of conditional essentiality
(1) How are the insertions normalized, e.g. in T4 between 0-10,000 kbp there are only a few insertions before selection in e.g. gene 20, 22, still this is claimed to be essential, do these genes meet the criterion stated in the methods. In this respect showing the distribution of TA sites along the genome, as done in Fig. 2 for genomic loci, would be informative, this can be also done on a sliding window, as it would get otherwise very detailed. It is a common pitfall with transposon mutagenesis to interpret low-insertion counts as essentiality. The authors determined the transposon sites before selection but how is this considered further for the evaluation of essentiality in the analysis? In general, this can be used to add confidence values. This is even more important for the Bas54 experiment where the coverage was lower. This should be commented in the text, re-evaluated based on the library before selection and continuous colouring should be considered.

(2) What if a transposon inserts in reverse to the sense strand, then there is an antisense transcript transcribed that could interfere with close transcripts from the other strand through base-pairing that encode possibly for essential genes. In essence, this could lead to an overestimation of essential genes. The authors should investigate this by comparing the strand orientation of insertions, the impact of the selection step on the ratio, and discuss it.

Minor comments

In general, the manuscript should provide more context and details about the method starting from expression, selection mechanism of Cas13a, and so on, this is important and can be edited in the manuscript.

Please consider also to switch Fig. 2 and 3, as it goes a back and forth between phage essentiality screening and anti-defence/clinical isolate screening.

Ext. Data. Fig. 2b,c and other similar panels, please comment on the following observation: If before selection only about 5 genes had insertions in all TA-sites in panel b, and then there is selection and expansion performed, how can it be that then in

panel c 150 genes have insertions in all TA-sites, where do these additional ones come from during the selection if these were not initially there?

A common reason for lack of insertions is blocking of loci by DNA-binding proteins, is there anything described for the region 0-10k in T4. The authors could dwell on this in the discussion to make also the reader aware that low-insertion counts can be possibly interpreted.

The prominently positioned rnlA example is intriguing to show sub-gene insertion effects, but ultimately the transposon breaks the open reading frame coding for the C-terminal domain, that was also ensured in general through stop codons in all three frames in the transposon. Would this work also for the N-terminal domains only? Would it require transcription/translation initiation sites in the transposon? This should be clarified in the text as a limitation related to sub-gene resolution claims.

Other phages do encode or inject RNAPs to transcribe their genome from distinct promoters where still the host RNAP could work. But, in cases like nucleus-forming phages or shutdown of the host-RNAP the anti-CRISPR protein must be transcribed from phage promoters. This scenario requires to add phage-own promoters for transposon expression, please discuss this in the manuscript.

Again nucleus-forming phages, require the transposase to be imported into the phage nucleus to integrate into the amplified phage genome, so here fusion of the transposase to a localisation protein would be required, please discuss this in the manuscript.

Do DNA modifications impair the transposon insertion, usually cytosine modifications are reported for phages, the here selected system does insert at TA-sites, so no conflict, but this should be discussed as a potential limitation.

In general, a more continuous colour coding for essentiality would be interesting to see, as there are sometimes effects but these are omitted, e.g. with vs.1 in Fig. 2.

Please comment also on the two phenotypes related to anti-defence gene knockouts, why in some cases there are no transposons detected and in others there are still many detected at a lower rate, can this be related to the efficiency of the system and the number of selection rounds undertaken. Can this be shown based on a quantitative PCR readout of the locus that probes for insertions and no-insertions, where after more rounds there is an enrichment for the wt? It would be very helpful for the community, to show how defined loci can be probed without running a full sequencing experiment that would also allow to test the dynamics of defined systems and their essentiality.

Please discuss in the main text the impact of transposon insertions on transcriptional units and termination events, or if these can be excluded.

As many phages encode for anti-CRISPR proteins, this should be also discussed as a limitation.

p. 4 l. 55: Please give more information about the approach of these methods and the clear limitations. It will also make clear where the advantages are related to transposon-based approach.

p. 4 l. 59: It is not always a phenotypic selection required.

p. 4 l. 62: An efficient selection marker by an essential phage gene was reported, it should be rather universal markers.

p. 5 l. 78 and l. 80: The LseCas13a targets RNA not ultimately the gene, but by transcript please correct this as if a gene is not transcribed it will not be targeted. In addition, it would be good to mention that mRNA modifications are not really existing in phage transcripts, this adds to the point that the method is universal at the selection.

p. 5 l. 79: The reader may ask, why the targeting of a single mRNA from a phage (even possibly a non-essential one), is leading to a selection, please comment on the working mechanism of selection.

p. 5 l. 86f.: There are some intermediate steps to be considered that is important to be clarified here. It would be important to mention the promoter that was used here with the acrVIA1 gene and to comment that this must be expressed early in infection to allow for inactivation by Cas13a that may likely target later genes. In this context, the current version of the manuscript does not mention which transcript is targeted by Cas13a in the main text, please add this in this paragraph, and explain also why targeting a major capsid protein is an important choice for the method.

p. 6 l. 102f. add reference to Fig. 1b again.

Discussion: Please comment on the transferability to other species, the LseCas12a can be very likely transferred to Pseudomonas phages, as this system was used previously in Pseudomonas (PMID: 36316452).

Fig. 1b: I could not find a single gene with reduced fitness colouring, is this true, or is there a lack in colouring?

p. 28: please state the promoters used for the acr

p. 29, l. 532: Is here “clones” correct or should it be plaques.

p. 30: please state at which MOI the cells were infected with T4 to generate the library, also for the other phages.

Fig. 1a, copy numbers of the plasmids should be indicated, and in the selection step the plasmid is missing. The blue transposase indication vs regular black genome in the bottom line will never be visible in b/w printouts, please mark it more prominently. The blue lines indicating transposon insertions show gaps between the genes, but actually, the intergenic regions are much more prone towards transposon insertions. The transposon insertion is not well depicted as there is no wt phage genome illustrated in the upper left part. The mechanism of inhibition by anti-CRISPR protein does not become evident, e.g. if the yellow transcript part (target) is transcribed at the same time as the blue (acr) the Cas13a will get activated, time-dependent events should be depicted clearly.

Extended Data Fig. 2B and similar other panels, essential and non-essential labels do not play a role here, and should be removed.

Reviewer #3 (Remarks to the Author):

All my comments have been integrated into the other reviewer's submission.

****

Version 1:

Reviewer comments:

Reviewer #1

(Remarks to the Author)
Many thanks to the authors for addressing our comments through additional experiments and edits to the manuscript and figures. The additional validation of essentiality calls in T4 and Bas54 and the additional characterization of the transposon libraries substantially strengthen the manuscript and support the general utility of HIDEN-SEQ, and the edits have made the manuscript easier to follow. In total we congratulate the authors on developing HIDEN-SEQ, which we expect will be a valuable resource for the research community.

Our remaining comment regards the discussion of polar effects. We appreciate the edits and the additional data in the revised manuscript but we still find the descriptions to be somewhat misleading. The authors appear to insist on their point that HIDEN-SEQ is not subject to polar effects, but this argument is 1) implausible given all the possible mechanisms that can give rise to polar effects (effects on transcript stability, transcript interference, translational coupling, and more); and 2) essentially impossible to prove especially without extensive documentation of a complete lack of polar effects across all phages. The experiment with a modified transposon that includes a transcriptional terminator is informative and provides strong evidence that the lack of a transcriptional terminator in the transposon used for HIDEN-SEQ mitigates some types of polar effects. It does not, however, allow for the inference that the remaining essentiality calls are not affected by polar effects. In addition, polar effects may particularly confound some of the intermediate fitness effects.

We recognize that it is not necessary for the authors to entirely rule out the possibility of polar effects. Indeed, HIDEN-SEQ has substantial utility regardless, particularly as a tool to rapidly nominate genes for further study, and therefore we feel it would in no way diminish the impact of this study to accurately describe their findings and limitations with regards to polar effects. Statements such as “polar effects are not a major confounder” are much too vague. We recommend that the authors further modify their language, specifically the statements in lines 136-138 of the results section (“These results strongly suggest that polar effects do not significantly distort the HIDEN-SEQ data generated with our transposon that lacks a transcriptional terminator.”) and lines 323-332 of the discussion (especially “…our data indicate that polar effects are not a major confounder of HIDEN-SEQ results…”). For example, lines 136-138 could be a statement that the results suggest that the use of a transposon without a transcriptional terminator mitigates some types of polar effects and improves the accuracy of essential gene identification, and the discussion could state that the authors accurately recall essential genes of T4, aided in part by the lack of a transcriptional terminator in their transposon, but that polar effects can nonetheless influence fitness effects of insertions as well as results in other phages.

(In case the authors do want to continue making the claim that HIDEN-SEQ is not confounded by polar effects, the burden would be on them to prove it. Specifically, they would have to demonstrate that each call that could in principle arise from a polar effect is accurate. For example, from an inspection of the Bas54 essentiality map, many essentiality/partial essentiality “calls” have patterns that would be consistent with polar effects in principle, including 26-27, 86, 100-103, 134, 181-183, and 196, among others. We did not cross-reference these genes against known databases of essential genes in related phages, but none of these genes were included in the orthogonal validation that the authors provided.)

On a minor note, the authors describe the insertion bias alongside their Bas54 transposon library. Might it be more appropriate to introduce this effect earlier, during the characterization of the T4 library?

Reviewer #2

(Remarks to the Author)
I appreciate the authors' revisions. My previous concerns have been adequately addressed.

Reviewer #3

(Remarks to the Author)
All of my comments have been incorporated into the submission by the other reviewer.

Decision Letter:

Our ref: NMICROBIOL-25124570A

17th June 2026

Dear Dr. Harms, dear Alexander,

Thank you for submitting your revised manuscript "Systematic mapping of bacteriophage gene essentiality with HIDEN-SEQ" (NMICROBIOL-25124570A). It has now been seen by the original referees and their comments are below. The reviewers find that the paper has improved in revision, and therefore we'll be happy in principle to publish it in Nature Microbiology, pending minor revisions to satisfy the referees' final requests and to comply with our editorial and formatting guidelines.

If the current version of your manuscript is in a PDF format, please email us a copy of the file in an editable format (Microsoft Word or LaTex)-- we can not proceed with PDFs at this stage.

We are now performing detailed checks on your paper and will send you a checklist detailing our editorial and formatting requirements in a couple of weeks. Please do not upload the final materials and make any revisions until you receive this additional information from us.

Thank you again for your interest in Nature Microbiology Please do not hesitate to contact me if you have any questions.

Sincerely,

Reviewer #1 (Remarks to the Author):

Many thanks to the authors for addressing our comments through additional experiments and edits to the manuscript and figures. The additional validation of essentiality calls in T4 and Bas54 and the additional characterization of the transposon libraries substantially strengthen the manuscript and support the general utility of HIDEN-SEQ, and the edits have made the manuscript easier to follow. In total we congratulate the authors on developing HIDEN-SEQ, which we expect will be a valuable resource for the research community.

Our remaining comment regards the discussion of polar effects. We appreciate the edits and the additional data in the revised manuscript but we still find the descriptions to be somewhat misleading. The authors appear to insist on their point that HIDEN-SEQ is not subject to polar effects, but this argument is 1) implausible given all the possible mechanisms that can give rise to polar effects (effects on transcript stability, transcript interference, translational coupling, and more); and 2) essentially impossible to prove especially without extensive documentation of a complete lack of polar effects across all phages. The experiment with a modified transposon that includes a transcriptional terminator is informative and provides strong evidence that the lack of a transcriptional terminator in the transposon used for HIDEN-SEQ mitigates some types of polar effects. It does not, however, allow for the inference that the remaining essentiality calls are not affected by polar effects. In addition, polar effects may particularly confound some of the intermediate fitness effects.

We recognize that it is not necessary for the authors to entirely rule out the possibility of polar effects. Indeed, HIDEN-SEQ has substantial utility regardless, particularly as a tool to rapidly nominate genes for further study, and therefore we feel it would in no way diminish the impact of this study to accurately describe their findings and limitations with regards to polar effects. Statements such as "polar effects are not a major confounder" are much too vague. We recommend that the authors further modify their language, specifically the statements in lines 136-138 of the results section ("These results strongly suggest that polar effects do not significantly distort the HIDEN-SEQ data generated with our transposon that lacks a transcriptional terminator.") and lines 323-332 of the discussion (especially "…our data indicate that polar effects are not a major confounder of HIDEN-SEQ results…"). For example, lines 136-138 could be a statement that the results suggest that the use of a transposon without a transcriptional terminator mitigates some types of polar effects and improves the accuracy of essential gene identification, and the discussion could state that the authors accurately recall essential genes of T4, aided in part by the lack of a transcriptional terminator in their transposon, but that polar effects can nonetheless influence fitness effects of insertions as well as results in other phages.

(In case the authors do want to continue making the claim that HIDEN-SEQ is not confounded by polar effects, the burden would be on them to prove it. Specifically, they would have to demonstrate that each call that could in principle arise from a polar effect is accurate. For example, from an inspection of the Bas54 essentiality map, many essentiality/partial essentiality "calls" have

patterns that would be consistent with polar effects in principle, including 26-27, 86, 100-103, 134, 181-183, and 196, among others. We did not cross-reference these genes against known databases of essential genes in related phages, but none of these genes were included in the orthogonal validation that the authors provided.)

On a minor note, the authors describe the insertion bias alongside their Bas54 transposon library. Might it be more appropriate to introduce this effect earlier, during the characterization of the T4 library?

Reviewer #2 (Remarks to the Author):

I appreciate the authors’ revisions. My previous concerns have been adequately addressed.

Reviewer #3 (Remarks to the Author):

All of my comments have been incorporated into the submission by the other reviewer.

INTERNAL USE ONLY AIP modules required: ["Life Science"]

Version 2:

Decision Letter:

14th July 2026

Dear Professor Harms, dear Alexander

I am pleased to accept your Article "Systematic mapping of bacteriophage gene essentiality with HIDEN-SEQ" for publication in Nature Microbiology. Thank you for having chosen to submit your work to us and many congratulations.

Please note that though this paper has been officially accepted, we will hold off on publication until the associated companion paper is also ready to go. I am trying my hardest to keep the ball moving on that one so there's not too much of a delay. Thanks for understanding and for the patience.

Over the next few weeks, your paper will be copyedited to ensure that it conforms to Nature Microbiology style. We look particularly carefully at the titles of all papers to ensure that they are relatively brief and understandable.

Once your paper is typeset, you will receive an email with a link to choose the appropriate publishing options for your paper and our Author Services team will be in touch regarding any additional information that may be required. Once your paper has been scheduled for online publication, the Nature press office will be in touch to confirm the details.

You may wish to make your media relations office aware of your accepted publication, in case they consider it appropriate to organize some internal or external publicity. Once your paper has been scheduled you will receive an email confirming the publication details. This is normally 3-4 working days in advance of publication. If you need additional notice of the date and time of publication, please let the production team know when you receive the proof of your article to ensure there is sufficient time to coordinate. Further information on our embargo policies can be found here:
https://www.nature.com/authors/policies/embargo.html

After the grant of rights is completed, you will receive a link to your electronic proof via email with a request to make any corrections within 48 hours. If, when you receive your proof, you cannot meet this deadline, please inform us at rjsproduction@springernature.com immediately. You will not receive your proofs until the publishing agreement has been received through our system

Due to the importance of these deadlines, we ask you please us know now whether you will be difficult to contact over the next month. If this is the case, we ask you provide us with the contact information (email, phone and fax) of someone who will be able to check the proofs on your behalf, and who will be available to address any last-minute problems.

Acceptance of your manuscript is conditional on all authors' agreement with our publication policies (see https://www.nature.com/nmicrobiol/editorial-policies). In particular your manuscript must not be published elsewhere.

Authors may need to take specific actions to achieve compliance with funder and institutional open access mandates. If your research is supported by a funder that requires immediate open access (e.g. according to Plan S principles or the NIH public access policy) then you

should select the gold OA route, and we will direct you to the compliant route where possible. Because authors warrant under our subscription licensing terms that they haven't committed to licensing any version of their article under a licence inconsistent with the terms of our agreement – including the applicable embargo period – publication under the subscription model isn't suitable for authors whose funders require no embargo.

If you have any questions about our publishing options, costs, Open Access requirements, or our legal forms, please contact ASJournals@springernature.com

An online order form for reprints of your paper is available at https://www.nature.com/reprints/author-reprints.html. All co-authors, authors' institutions and authors' funding agencies can order reprints using the form appropriate to their geographical region.

We welcome the submission of potential cover material (including a short caption of around 40 words) related to your manuscript; suggestions should be sent to Nature Microbiology as electronic files (the image should be 300 dpi at 210 x 297 mm in either TIFF or JPEG format). Please note that such pictures should be selected more for their aesthetic appeal than for their scientific content, and that colour images work better than black and white or grayscale images. Please do not try to design a cover with the Nature Microbiology logo etc., and please do not submit composites of images related to your work. I am sure you will understand that we cannot make any promise as to whether any of your suggestions might be selected for the cover of the journal.

You can now use a single sign-on for all your accounts, view the status of all your manuscript submissions and reviews, access usage statistics for your published articles and download a record of your refereeing activity for the Nature journals.

To assist our authors in disseminating their research to the broader community, our SharedIt initiative provides you with a unique shareable link that will allow anyone (with or without a subscription) to read the published article. Recipients of the link with a subscription will also be able to download and print the PDF.

As soon as your article is published, you will receive an automated email with your shareable link.

With kind regards,

P.S. Click on the following link if you would like to recommend Nature Microbiology to your librarian http://www.nature.com/subscriptions/recommend.html#forms

** Visit the Springer Nature Editorial and Publishing website at www.springernature.com/editorial-and-publishing-jobs for more information about our career opportunities. If you have any questions please click here.**

Open Access This Peer Review File is licensed under a Creative Commons Attribution 4.0 International License, which permits use, sharing, adaptation, distribution and reproduction in any medium or format, as long as you give appropriate credit to the original author(s) and the source, provide a link to the Creative Commons license, and indicate if changes were made.
In cases where reviewers are anonymous, credit should be given to 'Anonymous Referee' and the source.
The images or other third party material in this Peer Review File are included in the article's Creative Commons license, unless indicated otherwise in a credit line to the material. If material is not included in the article's Creative Commons license and your intended use is not permitted by statutory regulation or exceeds the permitted use, you will need to obtain permission directly from the copyright holder.
To view a copy of this license, visit https://creativecommons.org/licenses/by/4.0/

Response to reviewers:
"Systematic mapping of bacteriophage gene essentiality with HIDEN-SEQ" (NMICROBIOL-25124570-T)

We are grateful for the helpful comments that we have addressed when composing our revised manuscript as described in the point-by-point response below. All line numbers refer to the clean revised manuscript.

Response to editorial comments

- In particular, the referees independently brought up three major concerns. First, some issues with the lack of validation of essentiality, which will require additional analysis it seems. Second, there were also overlapping concerns about polar effects as potential confounders to essentiality, and this will also require some assessment. Finally, the referees also agree that some claims are overblown, and these will need to be toned down a bit in line with the conclusions as supported by the data.

Response: We are grateful for this editorial summary of the reviewer comments that was an important guidance for us when revising the manuscript. All concerns have been addressed as described in this point-by-point response to the reviewer comments.

Response to Reviewers #1/3

- Research on phage-bacteria interactions has driven biotechnological advances and inspired clinical treatments against antibiotic-resistant pathogens. However, a substantial fraction of genes in most phage genomes have unknown functions, limiting our understanding of phage biology. To address this gap, the authors present HIDEN-SEQ, an adaptation of Tn-seq for phages. A key innovation is the use of an anti-CRISPR protein as a selectable marker, which allows for enrichment of transposon mutants on a bacterial host expressing the corresponding CRISPR-Cas system. To benchmark their method, the authors use HIDEN-SEQ to retrieve known essential and conditionally essential genes in the model phage T4. The authors then implement HIDEN-SEQ to map essential genes in phylogenetically distinct phages. Based on these analyses, they identify novel anti-host defense genes in multiple phages, highlighting the utility of the method for biological discovery. A particular strength of the manuscript is the development of a custom gene essentiality classification pipeline to account for the smaller size and lower complexity of phage genomes.
- Overall, this manuscript is likely to be of high interest to the microbiology research community. Although two other recent pre-prints describe conceptually similar methodologies as HIDEN-SEQ, differences in the mutagenesis strategy and analytical pipeline of HIDEN-SEQ make it original and complementary to the other methodologies. In addition, all three groups have applied their methods to distinct phages, revealing novel information about their biology. Nevertheless, there are technical and conceptual issues that need to be addressed prior to publication. These issues primarily relate to a lack of validation of essentiality calls, a superficial treatment of potential polar effects, and limited characterization of some of the transposon mutant libraries, as further outlined below.

Response: We thank these reviewers for their positive assessment of our work and have addressed the issues as described below.

- Major Comments
  1. Lack of orthogonal validation of gene essentiality calls
  The manuscript contains no direct validation of essentiality calls derived from HIDEN-SEQ, for neither T4, Bas37, nor Bas54. Although overall the pipeline appears to work robustly, orthogonal validation would instill confidence in the generality and accuracy of the method. Such validation would be particularly pertinent i) for the cases in which HIDEN-SEQ yields results that conflict with previous reports, such as for the six T4 genes that appeared essential by HIDEN-SEQ (lines 282-283) but were reported as not essential in previous studies (ref. 10) and ii) or for the differences in essentiality patterns between Bas37 and T4, despite the high degree of relatedness between these phages (Fig. 3A, Ext. Fig. 4D; lines 178-185). Validation could consist of targeting a subset of putative non-essential and essential genes by orthogonal methods, such as genetic knockout, Cas13a inhibition, or anti-sense oligos.

Response: We thank these reviewers for their overall judgement that HIDEN-SEQ "appears to work robustly". In addition, we agree that additional validation of gene essentiality calls made by a new technology like HIDEN-SEQ is important to show the reliability of the approach. Importantly, we like to highlight that already our original submission contained a lot of validation of gene essentiality calls when we verified the *conditional* essentiality of specific genes as predicted by HIDEN-SEQ. For this we had knocked out more than a dozen genes across the three phages T4, Bas37, and Bas54 which HIDEN-SEQ suggested were not essential to infect *E. coli* K-12 in LB medium at 37°C. Indeed, we readily obtained these knockouts. HIDEN-SEQ had also predicted that these genes would be required for viral growth under specific conditions which we could also confirm (Figs. 2f and 4, Extended Data Figs. 3d, 8). Three *additional* genes of Bas54 were conditionally essential for growth only on the CFE311 strain (*bas54_0151*, *bas54_0150*, *bas54_0063*) and one in CFE303 strain (*bas54_0108*) but not included in the manuscript because we are still studying the underlying biology. The knockouts of all these genes also show a severe growth defect on CFE311/CFE303 compared to the K-12 control:

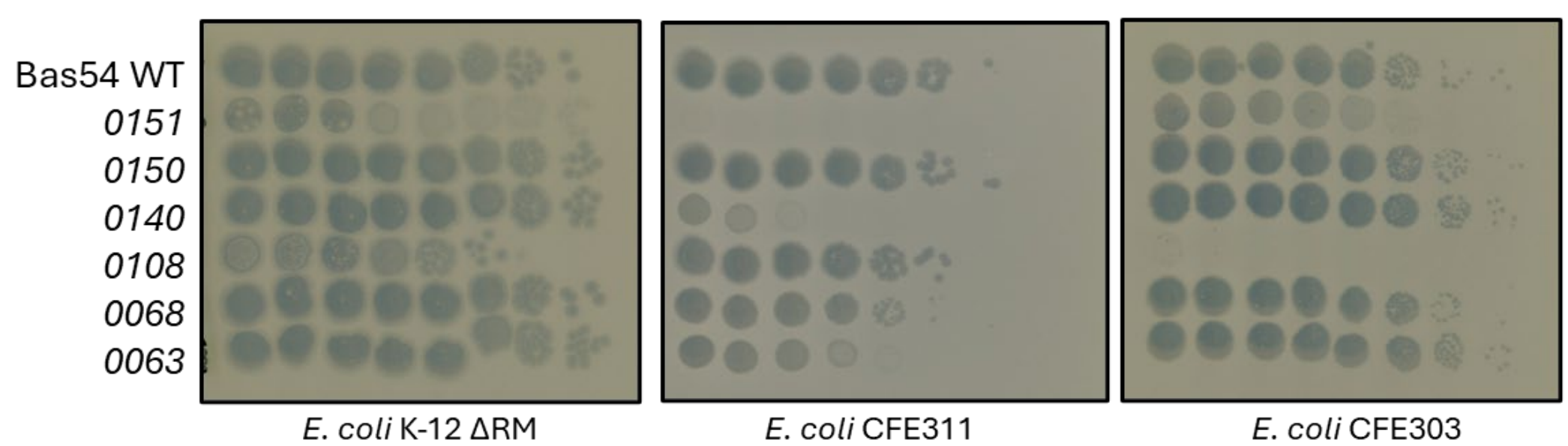

Regarding the very few genes where previous work reported no essentiality but HIDEN-SEQ detected few transposon insertions: We had focused on the *wac* gene that was most prominent among these. No knockout of *wac* is possible, also not with complementation *in trans*, suggesting that the integrity of this locus is critical for viral viability (Supplementary Fig. 2). The previous work highlighted by these reviewers had not truly knocked out this gene but studied an amber mutant, i.e., a conditional translational inhibition. This is different because it leaves the targeted locus intact as a whole and is also prone to residual translation which could result in the observed minor discrepancy. This had been mentioned in the main text already in the first submission (now lines 312-322). It may

thus not be a true discrepancy but rather a reflection of the different genetic methodology that has been used to study gene essentiality.

Regarding the two differences observed between Bas37 and T4: HIDEN-SEQ detects that only the 5' part of *rnlA* is essential in phage T4 while the orthologous gene in Bas37 (*bas37_0061*) is completely essential (Fig. 3a). We have now attempted targeted disruption of *bas37_0061* at a site where a targeted disruption was viable in T4 (site 1 in Fig. 1c; new data in Extended Data Fig. 5a). Only very few plaques for the *bas37_0061* disruption were obtained, and those clones with a true disruption (1/3/4 in the agarose gel) have a growth defect (spotting series on the left). This indicates that disruption of *bas37_0061* may originally be tolerated but that transposon mutants are than rapidly depleted during outgrowth of the library. Similar to *rnlA*, the *regA* gene was found to be not essential in T4 but its ortholog (*bas37_0161*) was essential in Bas37 (Fig. 3a). We have now attempted targeted disruptions of this gene at a site that was tolerated in HIDEN-SEQ for T4 but not Bas37 (Extended Data Fig. 5b). Plaques for disruption mutants were obtained in both cases, but the true disruption mutants of Bas37 (red; clones 2-4 on the gel and the plate) had a severe growth defect while those in T4 did not. Like for *rnlA*, this suggests that the Bas37 mutants in *bas37_0161* in HIDEN-SEQ would be rapidly depleted. Our results with targeted gene disruption thus confirm the HIDEN-SEQ results and suggest true biological differences between T4 and Bas37.

Finally, it seemed most important to us to independently validate the gene essentiality predicted by HIDEN-SEQ in Bas54 rather than in T4 / Bas37 because our results in those T-even phages were already almost perfectly (with tiny differences like *wac*; see above) congruent with the literature. Previous work on other technologies like CRISPRi-ART had significantly lower fidelity and – in case of this mentioned example – missed 25% of the known essential genes of phage T4 (Adler et al., Nature Microbiology (2025)). Conversely, Bas54 belongs to a viral group that had previously been barely studied. We therefore generated five new CRISPR-Cas9-selected knockouts with parallel *in-trans* complementation as an orthogonal approach to investigate gene essentiality for various Bas54 genes including two genes where essentiality was intuitively expected (e.g., the endolysin – *bas54_0202*) and genes coding for hypothetical proteins. In all five cases, the gene could be knocked out with induced *in-trans*-complementation but the viral mutants showed a very severe growth defect when the induction of complementation was abolished (Extended Data Fig. 6e). This fully confirms the gene essentiality predicted by HIDEN-SEQ using an orthogonal methodology.

- 2. Insufficient evaluation of potential polar effects
  A related concern is the superficial treatment of polar effects as potential confounders of essentiality calls. Such polar effects (both forward and reverse) are well-characterized confounders of Tn-seq in bacteria. Two recent pre-prints document that polar effects also affect essentiality calls from Tn-seq in phages. One pre-print (https://www.biorxiv.org/content/10.1101/2025.11.23.690004v2) explicitly notes some polar effects. Although the work in the pre-print uses a different transposon (that notably includes a transcriptional terminator, apparently by contrast to the transposon used in this manuscript) and involves a different phage, the precedent suggests that polar effects are important to consider. The second pre-print (https://www.biorxiv.org/content/10.64898/2025.12.19.695335v1) reported that insertions in some genes were enriched in the orientation that allowed the Acr to be transcribed in the same direction as phage genes, implicating polarity. Together, this precedent makes it important to rigorously assess the influence of polar effects on essentiality calls by HIDEN-SEQ.

- In this manuscript, the discussion of polarity is largely confined to a statement that "insertions upstream of essential genes were usually well tolerated" (lines 301-303), with the conclusion that polar effects were negligible. However, the presence of insertions upstream of a subset of essential genes does not exclude the possibility of polar effects confounding essentiality calls at other loci. In addition, the HIDEN-SEQ analysis pipeline appears to not consider biases in orientation of insertion. It would increase confidence in the accuracy of HIDEN-SEQ essentiality calls if the analysis pipeline differentiated insertions by orientation and factored such differences into essentiality calls. More broadly, we recommend that the authors conduct orthogonal validation of essentiality calls for a subset of genes within operons and expand their discussion of polar effects.

Response: We comment on polar effects and transposon directionality separately.

Generally, we agree that polar effects are a major consideration for any genetic technique based on gene disruption and recognize that this has been an important issue in the parallel preprint by Chan, Yee, et al. (bioRxiv, 2025) which used a transposon with transcriptional terminator. Given the small size of viral genomes (enabling manual analysis or insertion site patterns) and extensive prior knowledge of gene essentiality at least for T4, we tend to not completely agree on the dismissive comment of these reviewers that "the presence of insertions upstream of a subset of essential genes does not exclude the possibility of polar effects confounding essentiality calls at other loci". With our transposon, we simply do not observe any relevant number of genes possibly wrongly predicted to be essential that could be caused by effects of their gene disruption on neighbouring, truly essential genes. However, we now experimentally tested this by generating a transposon variant *with* transcriptional terminator to deliberately cause polar effects. Indeed, we readily observed a depletion of insertions for the transposon *with* terminator upstream of essential genes that was not observed for the transposon *without* terminator (new data in Extended Data Fig. 2a). For additional validation, we attempted to directly generate such insertions upstream of essential genes for the transposons with vs. without terminator by targeted genetic engineering (new data in Extended Data Fig. 2b). These data confirmed predictions by HIDEN-SEQ that only insertions without terminator were viable at these sites, likely due to interference of the terminator with transcription of the downstream essential genes. Based on these data and the lack of significant discrepancies between our work and prior knowledge on gene essentiality for T4, we thus conclude that polar effects do not greatly confound our results. We now show these results and highlight the importance of this topic at several positions in the manuscript, once in the results section (lines 128-138) and once in the discussion (lines 323-332). We also note that the small number of discrepancies (*inh*, *hoc*, *segE*, *uvsW*, *segC*, and *wac*) observed for T4 are unlikely to be explained by polar effects. In particular, for *hoc*, *uvsW*, and *wac*, genomic context argues strongly against polarity as the underlying cause. Specifically, *hoc* and *uvsW* are not upstream of essential genes, while *wac* is located at the end of a five-gene cluster with evidence of translational coupling (Miller et al., *Microbiol Mol Biol Rev* (2003)). As such, insertions on *wac* would not exert downstream polar effects.

Regarding the directionality of observed transposon insertions, these reviewers are right that a strong bias has been reported in the parallel preprint by Kyte et al. (bioRxiv, 2025). We also observe such a bias when sequencing transposon libraries after outgrowth and now show these data for both T4 and Bas54 (new data in Supplementary Fig. 4). However, when sequencing the initial libraries *before* outgrowth, such a bias is not observed (ibid.). This proves that the observed directionality is not due to a transposition bias but due to strong depletion of insertions that are not aligned with local gene directionality, possibly indeed due to detrimental effects of reverse transcription. Importantly, this directionality bias does in no way compromise the potency of HIDEN-SEQ to analyze transposons

at any possible insertion site because sufficient transposon mutants in either direction are always available since a high saturation of input libraries can always be obtained (shown with new data for the input libraries in Extended Data Fig. 1b, 4b, 6b and discussed below). We have now followed the reviewers' suggestion to report genome-wide data of transposon directionality for all phages (new data in Extended Data Tables 6-8), in the indicated figure (Supplementary Fig. 4), and in the main text (lines 217-220). Since the transposon orientation has no effect on the analysis of gene essentiality (because effectively all insertions after outgrowth are always aligned with gene directionality), we now highlight in the *Methods* (section on *Gene essentiality analysis*) that the orientation of transposon insertions was not considered as a parameter in our analyses.

3. Characterization of generated transposon mutant libraries

In several instances, it would be helpful for readers to have additional information on the quality of the generated transposon mutant libraries. This information is critical to evaluate how robust and generalizable the mutagenesis pipeline is.

- First, the authors do not directly measure how many transposon mutants are retrieved following mutagenesis. This knowledge would be helpful to infer whether all insertion sites in the genome were saturated prior to selection, which is a problem the authors ran into with their first Bas54 transposon library. A direct measurement of the number of mutants for some of their libraries (e.g. through plating of the phage lysate immediately after mutagenesis on permissive/restrictive hosts) would be helpful for readers.

Response: We fully agree with these reviewers that these are important (and related) points. In the revised manuscript, we now include the insertion density of relevant HIDEN-SEQ libraries in Extended Data Tables 6-8 (mentioned now also in the *Methods* section lines 700f). Furthermore, we now show data of input transposon mutant libraries before outgrowth or selection for T4 and – previously not available – Bas37 and Bas54 (all new data; shown in Extended Data Figs. 1, 4, and 6. These input libraries show extremely high saturation of possible transposon insertion sites (97.2% for T4, 87.1% for Bas37, and 91.6% for Bas54) as highlighted in those figures. Our results therefore unambiguously show that the efficiency of transposition is not at all a limiting factor for HIDEN-SEQ. We now refer to these new results at different positions in the manuscript, e.g., in the results section (lines 103-105, lines 216f), and in the different methods sections on the construction and analysis of HIDEN-SEQ libraries. Notably, we have also added a short paragraph to the methods section reporting on the technical difficulty of sequencing these input libraries due to extensive background (lines 678-686).

The requested plating of lysates after mutagenesis is shown for T4 in Supplementary Fig. 1a (Extended Data Fig. 1b in original submission), albeit not with a quantification of the number of transposon mutant plaques. We feel that the direct deep sequencing of the input libraries (see above) is far stronger evidence of high saturation than the plating could be, primarily because the plating can not give information on how many *different* transposon insertion mutants are in the pool which is at the end the critical point rather than their mere number. We have also removed the first Bas54 library (mentioned by these reviewers) from the revised manuscript because it did not provide further biological insight and showed that we had collected insufficient mutants after selection (not because we had too low saturation prior to selection). Instead, we now show the Bas54 input library which is more informative because it directly demonstrates high insertion density before outgrowth (Extended Data Fig. 6b).

- Second, the authors note that escape mutants that do not carry a transposon but become CRISPR-resistant would reduce the efficiency with which transposon mutants are retrieved.

They describe in their methods that they performed an experiment to estimate the frequency of escape mutants in their T4 transposon library (lines 537-539). However, they do not show data to support their claim that the frequency of escape mutants is low (lines 545-547). Please show these data.

Response: This is indeed an important point. However, we already show in Supplementary Fig. 1a (Extended Data Fig. 1b in original submission) that virtually no escape mutants are observed in a typical selection experiment for phage T4 with the selected crRNA (almost no plaques on the central plate). Similar data with a quantitative spotting in three replicates are shown for T4 in Extended Data Fig. 1A, for Bas37 in Extended Data Fig. 4A, and for Bas54 in Extended Data Fig. 6A. No escaper plaques can be observed in these experiments.

- Third, the authors generated two independent T4 transposon mutant libraries, with the only difference being deletion of rnlA in the host background. How well did essentiality calls by HIDEN-SEQ correlate between these datasets? This information could instill further confidence into the accuracy of the results.

Response: We thank these reviewers for highlighting this important point. However, this aspect is already covered in the main text in section "HIDEN-SEQ identifies known and novel conditionally essential genes in phage T4" (lines 155-158): "Importantly, when comparing the two independently generated T4 libraries, no other gene showed significant differences, highlighting both the specificity of bacterial immunity and the reproducibility of HIDEN-SEQ (Extended Data Fig. 3b)."

- Fourth, as part of this work the authors used HIDEN-SEQ to generate transposon mutant libraries for Bas37 and Bas54 but show only limited data that support the quality of the libraries. Do the authors have sequencing data from the transposon mutant libraries prior to selection, similar to T4 (Ext. Fig. 2A-B)? These data would increase confidence that the authors saturated mutagenesis of the Bas37 and Bas54 genomes, and that their pre-selection libraries were sufficiently complex in terms of insertions represented.

Response: These is indeed an important comment. In the revised manuscript, we now include data of input transposon mutant libraries before outgrowth or selection for all three phages (see also above). Briefly, we show such an input library for T4 as well as – previously not included – Bas37 and Bas54 (all new data; shown in Extended Data Figs. 1, 4, and 6). These input libraries show extremely high saturation of possible transposon insertion sites (97.2% for T4, 87.1% for Bas37, and 91.6% for Bas54) as highlighted in those figures. Our results therefore unambiguously show that the efficiency of transposition is not at all a limiting factor for HIDEN-SEQ. We now refer to these new results at different positions in the manuscript, e.g., in the results section (lines 103-105, lines 216f), and in the different methods sections on the construction and analysis of HIDEN-SEQ libraries. Notably, we have also added a short paragraph reporting to the methods section reporting on the technical difficulty of sequencing these input libraries due to extensive background (lines 678-686).

- Minor Comments
  General text
  1. Several statements in the manuscript are inappropriately hyperbolic, such as the multiple mentions of the "unprecedented depth and resolution" of HIDEN-SEQ and referencing the "critical breakthrough" of HIDEN-SEQ. Similarly, the authors claim that HIDEN-SEQ has "near base-pair resolution", but HIDEN-SEQ relies on mutagenesis via a mariner transposon that inserts specifically at TA dinucleotide sites, or roughly once every

8 base pairs at average GC content; the transposon itself causes a mutation that is much greater than a single base change; and the insertion sites are not distributed evenly through every phage genome. Describing the resolution of HIDEN-SEQ as "near base-pair" is misleading. Please modify the wording.

Response: We thank these reviewers for this point about the language used to describe potency and impact of HIDEN-SEQ. The formulation "near base-pair" (or similar) has been removed from the manuscript. In the revised manuscript, the only time "critical breakthrough" is used is to describe the relevance of molecular cloning (as an example for the importance of phage research for biotechnology, not referring to HIDEN-SEQ). However, compared to previously available technologies HIDEN-SEQ does provide "unprecedented depth and resolution", and this is the primary reason why we feel that this work is going to be interesting for a broad audience. We describe the specific advance provided by HIDEN-SEQ compared to previously available technologies in detail in the discussion section (lines 342-364).

- 2. The authors make a broad claim about the utility of HIDEN-SEQ for identification of conditionally essential genes. Such identification does suffer from the issue that the transposon mutant library must be generated in a bacterial host, such that any genes that are conditionally essential in that host will become depleted in insertions during library generation. Thus, there is strong directionality in conditional essentiality; defining conditional essentiality for genes that are essential in the library generation host (a large number of genes) but not in other hosts is difficult by this method. The authors get around this issue by constructing a transposon mutant library of T4 in a ΔrnlA host to demonstrate that dmd is conditionally essential in wild-type E. coli K-12. This approach is confirmatory, in that it requires prior knowledge of a potential conditional essentiality. As written, it is not a scalable tool for discovery. We recommend including an explicit discussion of limitations of conditional essentiality analysis with HIDEN-SEQ in the main text.

Response: We see the point made by these reviewers, but this is an inherent limitation of approaches based on genomic mutagenesis *in vivo*. To highlight this to readers, we now explicitly write in the discussion section that "Accordingly, conditional essentiality with HIDEN-SEQ can only be assessed for genes that are not essential in the host used to generate the library unless transposition would be performed *in vitro*" (lines 349-351). In addition, we added to a matching passage in the results section the formulation (new highlighted by underline) "This allowed us to directly evaluate whether insertions in known anti-defense genes, <u>which are not essential in the host used to generate the library</u>, would be selectively depleted when challenged by the targeted defense system" (lines 143-145).

However, we disagree that this means HIDEN-SEQ would not be "a scalable tool for discovery" because the majority of non-structural genes in most tailed phages are not essential under standard laboratory conditions (as shown also in our work and the two preprints by Chan, Yee, et al., *bioRxiv* (2025) or Kyte et al., *bioRxiv* (2025)). Within this accessible fraction, we identified many conditionally essential genes (for which we had no prior knowledge) and in some cases could also assign new functions to these genes with follow-up experiments.

- 3. Given that two other recent pre-prints describe methodologies that are conceptually similar to HIDEN-SEQ, it would be beneficial for the authors to include a brief comparison in the discussion, highlight aspects of their own methodology that are unique, and note overlapping or conflicting findings from the studies collectively.

Response: We agree with these reviewers that a comparison to the other two preprints (which had not been out when our work was submitted) would be interesting. However, the discussion section is already very long, so we focused on the main difference beyond technicalities which is the use of different host organisms and different phages: "During the revision of this manuscript, two independent studies reported related transposon sequencing approaches applied to one *Pseudomonas* phage and two *Prodigiosinella* phages" (lines 358-360).

- Abstract and Introduction
  4. It would be appropriate to introduce the HIDEN-SEQ acronym upon first mention in the abstract.

Response: Indeed, it's now included – thank you for highlighting this!

- 5. It would be helpful for readers to briefly note in the introduction the shortcomings of existing methods for mutagenesis of phage genomes, as a motivation for the development of HIDEN-SEQ. The authors have such statements in the discussion.

Response: We thank these reviewers for this suggestion. In the introduction we just briefly highlight that previously used methods "have offered valuable insights but remain laborious and not broadly applicable [references]". We had covered the topic with only this short sentence in the introduction because we are providing much more information in the discussion section (i.e., *after* we have described and shown HIDEN-SEQ) in direct comparison to our technology. There, a whole paragraph is dedicated only to this topic (lines 342-364). To avoid paraphrasing and doublings between introduction and discussion section, we have decided to leave the passage largely as it is (has been slightly expanded in the revised manuscript).

- All Figures
  6. For figures showing insertion site sequencing read counts across the genome, the authors cap the axis at 150 counts. Can the authors explain this choice? Is there any additional information encoded in higher counts?

Response: This limit to 150 counts was purely a choice for visualization to make the plots readable without losing the biological information. Higher counts are not discarded for the downstream analyses. We have now made this clear in figure legends etc. by highlighting that read counts are "shown up to a maximum of 150" (with "shown" as a new word).

- 7. Could the authors show quantifications of plaque-forming units per mL for their spot assays, for example as bar graphs, to make these results easier to interpret?

Response: We agree with these reviewers that the accessibility of illustrations is very important, but we feel that adding bar graphs to all phage spot assays would overload the figures and not add significant clarity. Spot tests also show important information such as the "lysis from without" (i.e., lysis zones without plaques) caused by viral adsorption and genome injection without significant replication. Bar graphs inherently mask these features and are thus also not commonly used in the field for this kind of assays (e.g., in the work by Adler et al., *Nature Microbiology* (2022)).

- Figure 1
  8. The authors mention in their discussion (lines 282-283) that T4 genes inh, hoc, segE, uvsW, segC, and wac appeared essential in their HIDEN-SEQ screen but were reported as not essential in previous studies (ref. 10). It would be helpful if this information was moved

up in the main text (lines 98-100), so that readers are aware of these discrepancies when assessing the data in Fig. 1B.

Response: Yes, we have added this information now at the indicated passage of the main text (now lines 110-114). Notably, targeted attempts to knock out *wac* failed even in the presence of *in trans* complementation, suggesting that this gene can indeed not be knocked out.

- 9. In Fig. 1C, the authors show validation of the domain-specific essentiality of rnlA, but do not describe this experiment in the main text.
- 10. For the rnlA finding, it would be helpful to provide some context regarding the known activity of rnlA and the known roles of the N-terminal and C-terminal domains.

Response: Indeed, these aspects have now been added to the main text (lines 120-126).

- 11. Line 107: Noun missing after "C-terminal" (or change to "C-terminus").

Response: Fixed, thank you for highlighting this oversight (changed to "C-terminal domain").

- Figure 2
  12. The specificity with which the authors identified dmd as conditionally essential in wild-type K-12 is nicely documented through the volcano plot in Ext. Fig. 3B. The authors note that they also identified conditionally essential genes identified through infection of hosts that express RexAB, CBASS, DarTG1, and DarTG2. Please provide similar volcano plots to give readers a sense of the sensitivity and specificity of these conditional essentiality analyses.

Response: We agree with these reviewers that showing volcano plots also for the results previously highlighted in a heatmap in Extended Data Fig. 3A (with single-locus zoom-ins in Fig. 2) would be more transparent and make the data more accessible. We have therefore now replaced the heatmap with volcano plots, i.e., for the data of RexAB, RnlAB, DarTG1, and DarTG2. No volcano plot is shown for CBASS (and there has also not been a heatmap entry before) due to the noisy data of this experiment caused by toxicity – this was mentioned in Methods and is now also mentioned in the legend of Extended Data Fig. 3a.

Notably, the data in Extended Data Figure. 3b highlighted by the reviewers are different because they do not compare the T4 library in presence vs. absence of ectopic RnlAB expression – this would be volcano plot for RnlAB in Extended Data Fig. 3a. Instead, Extended Data Fig. 3b compares the T4 library generated on the *rnlA* knockout to the T4 library generated on a wildtype host, i.e., it shows the effect of native *rnlAB* expression on *dmd* essentiality (and is a control for the HIDEN-SEQ reproducibility).

- 13. For Fig. 2G, it is unclear how "probability = 99.71%, E-value = 2.6 × 10^{-14}" was calculated based on the description of the structural analysis in the caption and methods.
  Figure 4

Response: Indeed, thank you! We now describe this result in more detail as "HHPred probability score 99.71%, *e*-value 2.6 × 10^{-14}."

- 14. The authors state that the E. coli clinical isolates for their work in Figure 4 were obtained through the Swiss NCCR AntiResist consortium, with more information to be

published elsewhere (lines 210-211). Are there any existing publications or pre-prints that could help the readers understand how these isolates were obtained?

Response: These reviewers are making an important point. Unfortunately, the originally planned publication of the NCCR AntiResist consortium is delayed and we thus now publish the mentioned uropathogenic strains with this manuscript. Their isolation and sequencing is now described in the methods section (lines 516-530 "Isolation of new uropathogenic *E. coli* strains", lines 759-770 "Genome sequencing and assembly of new uropathogenic *E. coli* strains"). The genomes have been uploaded to the ENA database under accession numbers mentioned in the *Data Availability* statement (lines 857-863) and will be available upon publication of our manuscript. We have updated the data reporting file with relevant information about the clinical work linked to the isolation of these strains.

- 15. Fig. 4 is difficult to follow while reading the main text, because the data for each finding is split across multiple panels. Please consider rearranging the volcano plot(s) and data from phage dilution assays such that they are paired by finding.

Response: We thank these reviewers for this helpful suggestion to improve the accessibility of Fig. 4. In the revised manuscript we have rearranged the data by finding as suggested.

- 16. The authors claim that bas37_0154 is involved in evading the Mokosh type I defense system of E. coli strain CFE331, supported by the observation that bas37_0154 knockout inhibits Bas37 propagation in CFE331 (Fig. 4B). Can the authors explain why insertions in bas37_0154 were not significantly depleted when HIDEN-SEQ was applied to CFE331 (in particular the q-value appears low; Fig. 4A)?

Response: This is indeed an interesting observation. *bas37_0154* was conditionally essential for infection of the CFE336 clinical isolate which is why we had made the knockout (see Extended Data Fig. 7a). The mutant was then included in the screening against cloned defense systems from multiple strains which also revealed the Mokosh type I phenotype mentioned by these reviewers. Similarly, the T4 *a-gt* gene which is important for growth on *E. coli* K-12 expressing Mokosh I of CFE331 is also not conditionally essential on CFE331 itself. We interpret this finding as suggesting that the Mokosh type I system is not limiting for T-even phage replication in the original context of CFE331 but becomes limiting upon ectopic expression in *E. coli* K-12. This could be, e.g., due to insufficient expression in CFE331, due to inhibition by anti-defense factors encoded by prophages in CFE331, due to synergy with a defense system of *E. coli* K-12, or due to enhanced potency of ectopic expression compared to the native genetic context. This is now included in a dedicated chapter of the Supplementary Discussion (and shortly in the legend of Fig. 4b).

- 17. For Fig. 4E, the phage dilution assay with K-12 ΔRM expressing the CFE311 Gao_Tmn_3 defense system is not discussed in the main text.

Response: Yes, indeed. We had initially shown this construct as a growth control for the viral knockout mutants, but this is also apparent from the *E. coli* K-12 ΔRM control. This construct has thus been removed from the revised manuscript.

- 18. Ext. Fig. 8F caption: "N-terminal" should read "N-terminus".

Response: Thank you – corrected to "N-terminus".

- Data Availability and Code

  In general, raw sequencing data for this work should be uploaded to a publicly accessible

database.
The authors cite all of the tools they used for read processing, alignment to transposons and phage genomes, and read subsampling (ref. 62-65). For essentiality analysis, the authors state that their custom code is available on Zenodo, with a digital object identifier that is yet to be released (lines 736-737). For conditional essentiality analysis, the authors cite the well-maintained Tn-seq analysis tool TRANSIT (ref. 63). They provide sufficient information on the parameters used for each of their tools that the analysis could theoretically be replicated. However, since we do not have access to the raw sequencing data, we were unable to reproduce the analyses.

Response: We thank these reviewers for highlighting this aspect of transparent data sharing. The raw reads have now been uploaded to the NCBI SRA database as BioProject PRJNA1454991 (to be released upon publication). The custom code is already available on Zenodo under digital object identifier https://doi.org/10.5281/zenodo.20179451.

Response to Reviewer #2

- Humolli et al. developed a transposon mutagenesis screen for phages in E. coli. In the first step, a marina transposon system was established with an anti-Cas13a factor that can insert randomly at TA sites in the phage genome, the resulting phages are then selected and enriched in a second step via a host where only phages with the transposon are able to inhibit the Cas13a system that targets an essential late phage transcript. The authors validate the approach for T4 phage and show that insertions can discriminate also among defined domain essentiality at sub-gene resolution for one gene rnlA. In the next step, this technology was used to test conditionally essential genes connected to anti-defence functions that were validated by expression or deletion of anti-phage defence genes. The authors identified a new anti-defence gene tk.4 against DarTG2 anti-phage defence, and others. In addition, the conditional essentiality was tested in minimal media and resulted in the essentiality of the valyl-tRNA synthetase. The approach was also further validated through application on a T4 relative phage with highly similar outcome that further supports the reproducibility. In addition, the approach was applied to a V5-like phage. Then the authors turned to screen different hosts, and realized that for the V5-like phage that encodes multiple tail fibers these appeared to be responsible for fitness in specific clinical isolates. The authors point also out an intergenic region that appears essential and remains uncharacterised among many other factors, mentioning that non-coding regions can be also targeted and reveal essentiality. Screening clinical isolates with the phage library in combination with the prediction of anti-phage defence genes, allowed to authors to pinpoint a hand full of interactions with phage genes that can negate anti-phage defence systems.
  The study utilizes latest available technologies and merges them into a novel system for phage research. The authors came up with a magnitude of blueprints how to make use of the phage transposon library in the context of conditional essentiality linked to anti-defence genes, nutrient availability, and uncharacterised regions. To put this into perspective, this represents one of the very first studies that makes use of transposon mutagenesis in phages coupling this to sequencing-based readouts. This study applies it e.g.

to a well characterized T4 phage and shows many novel insights also highlighting the still large discovery space in a highly studied phage. With this technology in hand, the study of Escherichia and other species infecting DNA-phages, will accelerate and allow to map the host-phage interface in unpreceded ways. As exemplified, this study will also guide the discovery of hundreds of anti-defence genes that could be utilised for tailored phage therapy. There remain several major points to address to improve the manuscript.

Response: We thank reviewer #2 for their positive assessment of our work and will address the different major points as described below.

- Major comments

  Claim: Portability beyond model phages

  The authors illustrate impressively the applicability in T4, a closely related phage, and a V5-like phage. Still, these phages are T4/V5-like DNA-phages, that represents only two out of 34+ taxonomic families from the currently available BASEL phage collection that was also established by the Harms lab. In general, the system requires genetics in the host, transposon integration, and selection. The study would obviously benefit from including other taxonomic families, e.g. the authors included two different Cas13a systems, of which one would very likely work in Pseudomonas, too, as previously used for selection, that could be included or discussed. More important, there are additional challenges on the phage side, that should be covered in a systematic way to solidify the claim to be applicable in many phage-host pairs or to make the limitations clear.

  (1) One requirement for the system to work is that the anti-CRISPR protein is expressed. In the current setting it is expressed from a host-like promoter by the host RNAP soon after infection. Does this promoter work also in other species, like Pseudomonas? Some evidence or references should be given, and explained that promoters may require adjustments for other species.

Response: We thank reviewer #2 for highlighting the importance of being able to use HIDEN-SEQ also in other phages or host organisms. In our work we show already the use in two very different kinds of *E. coli* phages (*Tevenvirinae* and *Vequintavirinae*), and recent preprints by other labs showed that similar technologies work in *P. aeruginosa* (Chan, Yee, et al., *bioRxiv* (2025)) or *Prodigiosinella* (Kyte et al., *bioRxiv* (2025)). Similar to the adaptation of TnSeq technologies to different bacterial organisms or specific biological setups, we thus anticipate that future studies will use HIDEN-SEQ also in other phages and host organisms. We now highlight this broad applicability more specifically in our discussion section and also mention specific challenges such as the possible need to adjust promoters for certain phages (lines 358-364).

- (2) Does the system work with phages like T5 and T7, that do shut down the host transcription that is required for the expression of the anti-CRISPR protein, or does it in this case require an early phage-derived promoter?
- (3) Are there limitations related to the partial injection of e.g. the T5 genome? Would the selection step work still with transposon insertions in the complete genome?
- (4) Some phages degrade the host genome rapidly, hence the transposon template should get degraded, too. Are there any limitations with the current system related to integration efficiency, e.g. in e.g. T5.

Response: Reviewer #2 is highlighting that the specific biology of a few phages might require specific adaptations of the anti-CRISPR expression. We agree on that, but we find that the exploration of such

specific adaptations for individual cases is beyond the scope of this current study that focuses on the development and applicability of HIDEN-SEQ in general. In our discussion section, we have now added a passage that highlights the possible requirement to make phage-specific adjustments to the technology in some specific cases (lines 358-364). We additionally like to highlight that also phage T4 degrades the host chromosome very early during the infection process (see, e.g., the review by Kutter et al., *Viruses* (2018)) which does not seem to affect the applicability of HIDEN-SEQ.

- Claim: Near-base-pair resolution.
  (1) The authors should take the coverage into account. What coverage would be required for a base-pair resolution claim, usually 5x coverage per base is considered, this is for T4 with 170 kbp about 800k insertions. But, Himar1 insert only into TA-sites, hence this is 14,474 TA sites x 5 is about 75k. How many transposition events were actually recorded? The method section gives an estimation that is purely based on theoretical assumptions. Which MOI was used, it is not stated in the method section? How many replication rounds were possible until the pre-selection library? The T4 library before selection was sequenced, this should be used to estimate the saturation quantitatively by comparing the number of unique insertions related to all predicted TA-sites and put into perspective to the number of events.

Response: Reviewer #2 is right that the coverage of transposon sites prior to selection is a critical consideration because it determines the maximal resolution that can be obtained with HIDEN-SEQ (see also related points of reviewer #1 above). In the revised manuscript, we now include the insertion density of relevant HIDEN-SEQ libraries in Extended Data Tables 6-8 (mentioned now also in the *Methods* section lines 698ff). Furthermore, we now show data of input transposon mutant libraries before outgrowth or selection for T4 and – previously not included – Bas37 and Bas54 (all new data; shown in Extended Data Figs. 1, 4, and 6. These input libraries show extremely high saturation of possible transposon insertion sites (97.2% for T4, 87.1% for Bas37, and 91.6% for Bas54) as highlighted in those figures. Our results therefore unambiguously show that the efficiency of transposition is not at all a limiting factor for HIDEN-SEQ. We now refer to these new results at different positions in the manuscript, e.g., in the results section (lines 103-105, lines 216f), and in the different methods sections on the construction and analysis of HIDEN-SEQ libraries. Notably, we have also added a short paragraph to the methods section reporting on the technical difficulty of sequencing these input libraries due to extensive background (lines 678-686).

- (2) In addition, the transposon insertions in the phage library before selection, can be quantified with quantitative PCR to give precise estimation about the integrated transposons in the phage particles before selection, and the number of phages is estimatable by a reference experiment, so quantitative numbers could be returned. This is essential to determine the coverage. It is also technologically important, as it provides the future users with a broadly accessible way to estimate the saturation of the library prior sequencing with defined primers for the transposon. Of note, the distribution is anyway not uniform, e.g. genes 20-23 show less insertions. If the coverage does not reach the initially mentioned number, the claim about base-pair resolution should be rephrased to sub-gene resolution, as this is sufficiently accurate for all claims, and well validated.

Response: Reviewer #2 is right to highlight the importance of high-coverage transposon libraries also with this second comment. We have already responded to a similar comment right above and highlighted new data reporting the saturation of possible transposon sites in our input libraries to be

97.2% for T4, 87.1% for Bas37, and 91.6% for Bas54 (now shown in Extended Data Figs. 1, 4, and 6). This would in principle certainly be enough to reach near-basepair resolution, but depending on the phage genome TA dinucleotide sites are typically present at a spacing of 5-10bp. We have thus removed the claim of “near-basepair resolution” from our manuscript. One could in theory also try to quantify the transposon copies in the experiment by quantitative PCR, but in our view this would not provide similarly useful information. Since only the number of transposon copies and not their position in the genome could be quantified, this approach would be not informative about the saturation of TA dinucleotide sites, i.e., the actually different transposon insertions in the library. Any quantitative PCR would also suffer from background caused by leftover plasmid fragments, transposition into the host chromosome, etc. which would be indistinguishable from transposon copies in the viral genome.

- Claim: Mapping of conditional essentiality
  (1) How are the insertions normalized, e.g. in T4 between 0-10,000 kbp there are only a few insertions before selection in e.g. gene 20, 22, still this is claimed to be essential, do these genes meet the criterion stated in the methods. In this respect showing the distribution of TA sites along the genome, as done in Fig. 2 for genomic loci, would be informative, this can be also done on a sliding window, as it would get otherwise very detailed. It is a common pitfall with transposon mutagenesis to interpret low-insertion counts as essentiality. The authors determined the transposon sites before selection but how is this considered further for the evaluation of essentiality in the analysis? In general, this can be used to add confidence values. This is even more important for the Bas54 experiment where the coverage was lower. This should be commented in the text, re-evaluated based on the library before selection and continuous colouring should be considered.

Response: Reviewer #2 is making several very important points in this comment to which we are responding separately, but there are also some misunderstandings about our procedure that need to be clarified.

About the relationship between observed transposon insertions and gene essentiality calls: As highlighted by this reviewer, we have a clear procedure for gene essentiality calls described in the respective part of the methods section (lines 703-735). Briefly, we did not interpret low insertion counts alone as evidence of essentiality (as suggested by this reviewer for a specific region encoding virion genes of the T4 genome). Instead, we considered several features together, including the number of TA dinucleotide sites in each gene, the fraction of those TA sites that carried insertions (saturation), the distribution of insertions across the gene, and the corresponding read counts. In addition, genes with $\leqslant$5 TA sites were excluded from classification because they do not provide enough insertion sites for a confident call. These values are provided for all libraries in the Extended Data Tables 6-8 with the data for the mentioned genes in the T4 genome in Extended Data Table 6. These tables also contain information about all TA sites in the different genomes as requested by this reviewer. Displaying them in some kind of illustration in a genome-wide manner would indeed not be possible due to their large numbers and quite consistent distribution across the genomes.

About the role of the input libraries for our analyses: The distribution of transposon sites before selection was not considered further in the evaluation of essentiality, only to determine the saturation of transposition. No gene essentiality calling has been done on the input/pre-selection libraries (despite the genes showing in blue in the original submission, now changed into gray to avoid confusion with HIDEN-SEQ classification categories). The input library is used ONLY to assess

transposition efficiency and genome-wide insertion distribution before outgrowth. The actual gene essentiality classification is based on comparison of libraries obtained after outgrowth/selection. To clarify this, we have now added this sentence in the Methods section "These libraries were sequenced only to assess transposition efficiency and overall library saturation before outgrowth. All analyses for gene essentiality were based on libraries obtained after outgrowth / selection and not these input libraries" (lines 683-686).

About the first Bas54 experiment with lower saturation: We have removed this library from the revised manuscript because it did not provide additional biological insight and showed that we had collected insufficient mutants after selection (not because we had too low saturation prior to selection). Instead, we now show the Bas54 input library which is more informative because it directly demonstrates high insertion density before outgrowth (Extended Data Fig. 6).

- (2) What if a transposon inserts in reverse to the sense strand, then there is an antisense transcript transcribed that could interfere with close transcripts from the other strand through base-pairing that encode possibly for essential genes. In essence, this could lead to an overestimation of essential genes. The authors should investigate this by comparing the strand orientation of insertions, the impact of the selection step on the ratio, and discuss it.

Response: We thank reviewer #2 for highlighting the importance of the relative orientation of transposon insertions to the directionality of the local genetic environment (see also the first major comment of reviewer #1). Briefly, we do not think that there is an "overestimation of essential genes" or other effects of directionality effects because there is an extreme bias in all libraries *after outgrowth* in a way that transposon insertions are almost always aligned with the transcription of local genes and almost never against it. We now show these data for both T4 and Bas54 (new data in Supplementary Fig. 4). However, when sequencing the initial libraries *before* outgrowth, such a bias is not observed (ibid.). This proves that the observed directionality is not due to a transposition bias but due to strong depletion of insertions that are not aligned with local gene directionality, possibly indeed due to detrimental effects of reverse transcription. Importantly, this directionality bias does in no way compromise the potency of HIDEN-SEQ to analyze transposons at any possible insertion site because sufficient transposon mutants in either direction are always available since a high saturation of input libraries can always be obtained (shown with new data for the input libraries in Extended Data Figs. 1b, 4b, 6b and discussed below). We now also report genome-wide data of transposon directionality for all phages (new data in Extended Data Tables 6-8), in the indicated figure (Supplementary Fig. 4), and in the main text (lines 217-220). Since the transposon orientation has no effect on the analysis of gene essentiality (because effectively all insertions after outgrowth are always aligned with gene directionality), we now highlight in the *Methods* (section on *Gene essentiality analysis*) that this that the orientation of transposon insertions was not considered as a parameter in our analyses. We also performed targeted insertions of the anti-CRISPR cassette in the reverse orientation relative to the gene at two independent loci (see below). In both cases, almost no plaques were obtained. We decided to not include these data in the revised manuscript to not overload it as the HIDEN-SEQ results alone already clearly demonstrate strong selection against the reverse-oriented insertions.

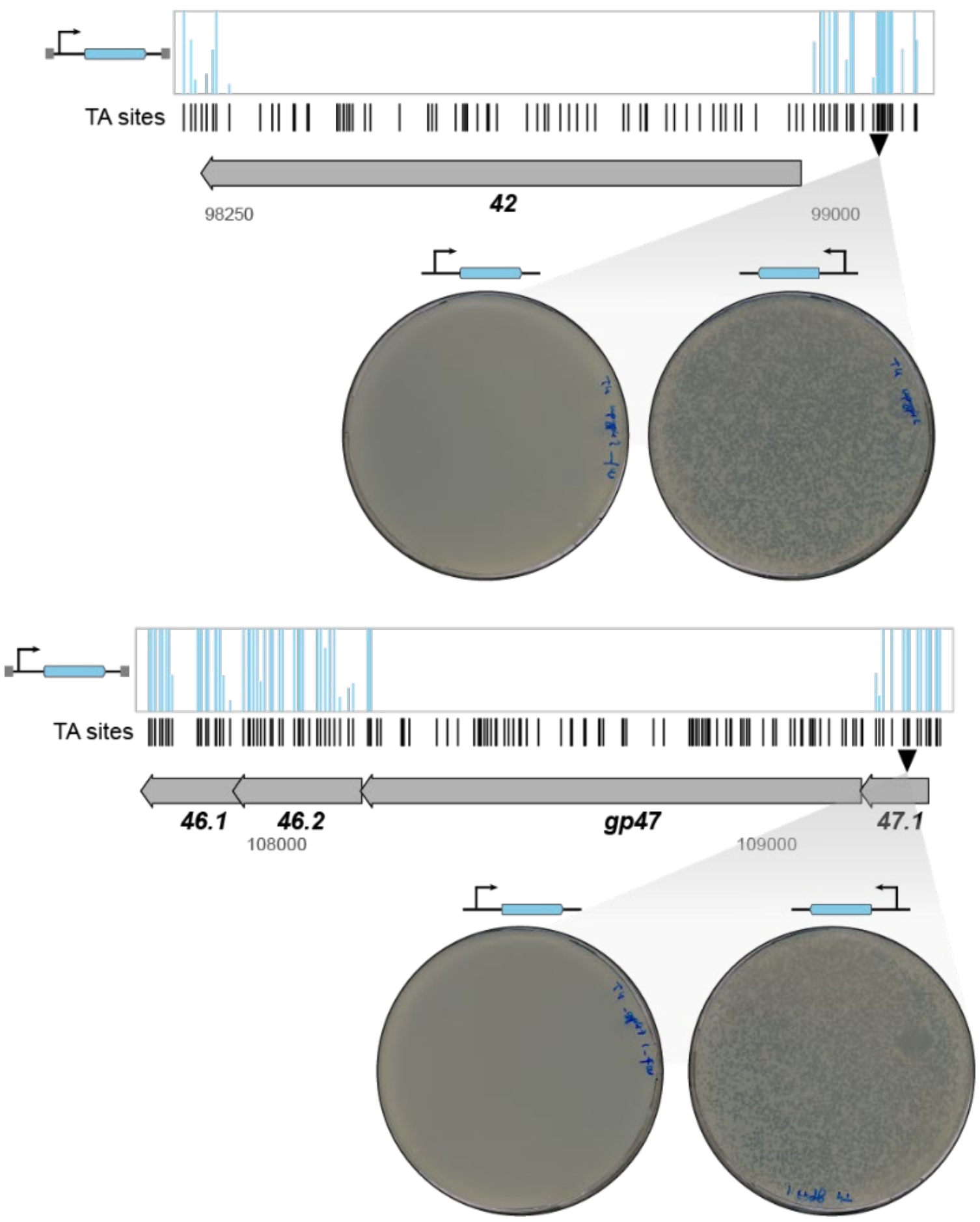

- Minor comments
  In general, the manuscript should provide more context and details about the method starting from expression, selection mechanism of Cas13a, and so on, this is important and can be edited in the manuscript.

Response: We thank reviewer #2 for this helpful comment. The revised manuscript now contains much more information about the details of the technology also in the main text (in the paragraph spanning lines 77-93).

- Please consider also to switch Fig. 2 and 3, as it goes a back and forth between phage essentiality screening and anti-defence/clinical isolate screening.

Response: Reviewer #2 is right that one could switch the order of figures 2 and 3. However, we decided to keep the order as is because it seems useful to us to first benchmark the technology on phage T4 (including conditional essentiality; Figures 1 and 2) before moving on to other phages.

- Ext. Data. Fig. 2b,c and other similar panels, please comment on the following observation: If before selection only about 5 genes had insertions in all TA-sites in panel b, and then there is selection and expansion performed, how can it be that then in panel c 150 genes have insertions in all TA-sites, where do these additional ones come from during the selection if these were not initially there?

Response: This reviewer is making a very good point – at first glance, it is indeed counter-intuitive that the outgrowth libraries after selection can show transposon insertions that were seemingly absent in the pre-selection and pre-outgrowth "input" library. Notably, we have now provided new data of the T4 input library (and sequenced analogous libraries for Bas37 and Bas54) for the revised manuscript which provide a much better view on the transposon mutants that were actually present before selection and outgrowth. There are two layers in our response to this point:

Firstly, the input libraries were technically much more difficult to sequence because transposon-specific amplification also captured residual donor plasmid DNA which we think underestimates the real insertion density. This explains why, after selection and outgrowth, many genes appear to have 100% of TA sites hit even though the input library did not show this. This means that these insertions were already present at low abundance in the starting pool but were not captured by sequencing. We have now also added a short paragraph reporting to the methods section reporting on the technical difficulty of sequencing these input libraries due to extensive background (lines 678-686).

Compared to the initial submission, we have now also added now the non-preprocessed data (meaning not removing low read counts) to better show the presence of low-abundance mutants. The new adjustment on the DNA library preparation improved the coverage also for T4 and we now updated the figure highlighted by this reviewer (Extended Data Figs 1c and 1d). Unlike before, the vast majority of genes now show (before selection + outgrowth) a very high saturation of TA sites with transposon insertions of >90%. Importantly, these data have been generated with exactly the same T4 DNA sample as before that has now been sequenced and analyzed again in a more sensitive way.

- A common reason for lack of insertions is blocking of loci by DNA-binding proteins, is there anything described for the region 0-10k in T4. The authors could dwell on this in the discussion to make also the reader aware that low-insertion counts can be possibly interpreted.

Response: We thank reviewer #2 for this interesting comment. The region mentioned by this reviewer is known to be essential (encoding structural genes of phage T4) and is not targeted by DNA-binding proteins in any specific way. However, the tight binding of proteins to DNA sequences could indeed generally affect transposition like many other biological phenomena in individual phages. To highlight the importance of this more generally, we have now added a sentence to the discussion that mentions the specific biology of individual phages as a hurdle for efficient transposon insertion-site sequencing (lines 360-364).

- The prominently positioned rnlA example is intriguing to show sub-gene insertion effects, but ultimately the transposon breaks the open reading frame coding for the C-terminal domain, that was also ensured in general through stop codons in all three frames in the transposon. Would this work also for the N-terminal domains only? Would it require transcription/translation initiation sites in the transposon? This should be clarified in the text as a limitation related to sub-gene resolution claims.

Response: This reviewer is right about these limitations of sub-gene resolution. We have now edited the text accordingly both in the results section ("sub-gene resolution of fitness analyses <u>in some cases</u>", line 120 with new text underlined in the quote) and the methods section (lines 714-719).

- Other phages do encode or inject RNAPs to transcribe their genome from distinct promoters where still the host RNAP could work. But, in cases like nucleus-forming phages

or shutdown of the host-RNAP the anti-CRISPR protein must be transcribed from phage promoters. This scenario requires to add phage-own promoters for transposon expression, please discuss this in the manuscript.

- Again nucleus-forming phages, require the transposase to be imported into the phage nucleus to integrate into the amplified phage genome, so here fusion of the transposase to a localisation protein would be required, please discuss this in the manuscript.
- Do DNA modifications impair the transposon insertion, usually cytosine modifications are reported for phages, the here selected system does insert at TA-sites, so no conflict, but this should be discussed as a potential limitation.

Response: We thank reviewer #2 for these suggestions and share the enthusiasm of this reviewer for nucleus-forming jumbo phages. In the revised manuscript we now explicitly highlight that phage-specific promoters or altered transposon setups to overcome DNA modifications could sometimes be required to adapt HIDEN-SEQ to new phages (lines 360-364). Regarding the localisation protein, we feel that this is a very specialized case relevant to a limited number of phages and thus did not explicitly highlight it. However, we find that such adaptations are clearly implied when we write "that the biology of individual phages may in some cases require tailored adjustments of the technology" in this sentence (lines 360-364).

- In general, a more continuous colour coding for essentiality would be interesting to see, as there are sometimes effects but these are omitted, e.g. with vs.1 in Fig. 2.

Response: Reviewer #2 is certainly right that a consistent and intuitive color coding is important for the accessibility of our illustrations. However, this comment is mostly about the classification that has been used for gene essentiality. We find that our five-level classification from "non-essential" to "essential" is already quite fine-grained because it contains four separate categories for genes where less insertions than randomly expected are observed, "reduced fitness", "intermediate", "strong fitness defect", and "essential. In our view, this is sufficient. The underlying calculations and considerations are described in detail in the two subsections of "Data analysis of HIDEN-SEQ libraries" within the *Methods* section. In the specific case of *vs.1* in Fig. 2, the gene was classified as non-essential in the reference library because insertions were detected at 88% of its TA sites. Accordingly, the blue colouring in Fig. 2 reflects essentiality in the reference library only, whereas red indicates genes identified as conditionally essential. We agree that this may have been confusing and have therefore revised the figures so that, when libraries from different conditions are compared, only the conditional essential genes are highlighted and the neighboring genes are shown in gray.

- Please comment also on the two phenotypes related to anti-defence gene knockouts, why in some cases there are no transposons detected and in others there are still many detected at a lower rate, can this be related to the efficiency of the system and the number of selection rounds undertaken. Can this be shown based on a quantitative PCR readout of the locus that probes for insertions and no-insertions, where after more rounds there is an enrichment for the wt? It would be very helpful for the community, to show how defined loci can be probed without running a full sequencing experiment that would also allow to test the dynamics of defined systems and their essentiality.

Response: Reviewer #2 is right to notice the slight differences in depletion of insertions in *bona fide* anti-defense genes in phages challenged with their targeted defense system. In some cases (*rIIAB* vs. RexAB, *adfN* vs. DarTG1) insertions are almost completely depleted, while the depletion is less strong (but significant in our calculations) in the other cases. We feel that this is largely a consequence of the

different strengths of the defense systems and, consequently, of the fitness defects caused by the disruption of the specific *bona fide* anti-defense genes. Reviewer #2 is certainly correct that one could try to quantify the rate of depletion for each defense / anti-defense pair using techniques like qPCR. However, we feel that this would be beyond the scope of this study and would not significantly strengthen our manuscript that is largely focused on the basics of HIDEN-SEQ and its application, e.g., to identify anti-defense genes. Future studies more focused on individual defense / anti-defense pairs could zoom in on individual cases more intensively and try to set up experiments as proposed by this reviewer.

- Please discuss in the main text the impact of transposon insertions on transcriptional units and termination events, or if these can be excluded.

Response: We interpret this comment of reviewer 2 as referring to possible polar effect of transposon insertions – it is certainly true that transposon insertions can in principle disrupt transcriptional units and termination events in viral genomes. The first major comment of reviewer #1 had focused on the topic in more detail, and there we describe several lines of evidence (including several sets of new data) which demonstrate that polar effects are unlikely to be a significant confounding factor for our HIDEN-SEQ setup. In line with this request of reviewer #2, we now highlight the importance of this topic at several positions in the revised manuscript, once in the results section (lines 128-138) and once in the discussion (lines 323-332).

- As many phages encode for anti-CRISPR proteins, this should be also discussed as a limitation.

Response: Reviewer #2 is correct that – in theory – phage-encoded anti-CRISPR proteins could be a problem if they target the CRISPR-Cas system used for the selection of transposition. However, anti-CRISPR proteins targeting CRISPR-Cas13a are extremely rare (Adler et al., *Nature Microbiology* (2022) and one of the two anti-CRISPR proteins used in our study (AIcrVIA3) has been AI-generated due to the lack of known natural options against the respective variant of CRISPR-Cas13a. We thus think that other limitations requiring phage-specific tailoring of the approach (promoter efficiency, DNA modifications, etc.) are much more important than these extremely rare anti-CRISPRs. To not overload the discussion section, we have thus decided to not specifically mention phage-encoded anti-CRISPR proteins and chose other examples when highlighting the occasional need for phage-specific adaptation (lines 360-364).

- p. 4 l. 55: Please give more information about the approach of these methods and the clear limitations. It will also make clear where the advantages are related to transposon-based approach.

Response: We thank reviewer #2 for highlighting that we should make the limitations of previously available techniques more clear to highlight the advantages of HIDEN-SEQ. This comment refers to a sentence in the introduction where we said that previously used methods "have offered valuable insights but remain laborious and not broadly applicable [references]". We had covered the topic with only this short sentence in the introduction because we are providing much more information in the discussion section (i.e., *after* we have described and shown HIDEN-SEQ) in direct comparison to our technology. There, a whole paragraph is dedicated only to this topic (lines 342-364). To avoid paraphrasing and doublings between introduction and discussion section, we have decided to leave the passage largely as it is (has been slightly expanded in the revised manuscript).

- p. 4 l. 59: It is not always a phenotypic selection required..

Response: Done – we have edited the text to say "before and after growth under a given condition" rather than "before and after phenotypic selection".

- p. 4 l. 62: An efficient selection marker by an essential phage gene was reported, it should be rather universal markers.

Response: Done – we have edited the passage to say "broadly applicable genetic selection markers".

- p. 5 l. 78 and l. 80: The LseCas13a targets RNA not ultimately the gene, but by transcript please correct this as if a gene is not transcribed it will not be targeted. In addition, it would be good to mention that mRNA modifications are not really existing in phage transcripts, this adds to the point that the method is universal at the selection.
- p. 5 l. 79: The reader may ask, why the targeting of a single mRNA from a phage (even possibly a non-essential one), is leading to a selection, please comment on the working mechanism of selection.

Response: Done – we have now added a more detailed description of CRISPR-Cas13a functionality to the text at this position of the introduction that highlights the targeting of *transcripts* (rather than genes), that these are typically not modified, and that target recognition triggers broad non-specific degradation of RNAs in the cell.

p. 5 l. 86f.: There are some intermediate steps to be considered that is important to be clarified here. It would be important to mention the promoter that was used here with the acrVIA1 gene and to comment that this must be expressed early in infection to allow for inactivation by Cas13a that may likely target later genes. In this context, the current version of the manuscript does not mention which transcript is targeted by Cas13a in the main text, please add this in this paragraph, and explain also why targeting a major capsid protein is an important choice for the method.

Response: We agree with this reviewer that these information of CRISPR-Cas13a selection are important and have edited the manuscript text accordingly, but we feel that adding too many technical details into the main text may make it less accessible to the broad audience of Nature Microbiology. The main text has now been edited to highlight that *acrVIA1* was expressed from "a strong constitutive promoter" right before the text explains selection "with a potent crRNA targeting the transcripts of an essential T4 gene coding for the major capsid protein" (lines 81-85), now highlighting the targeted transcript. Exact details like the formal names of the promoter are included in the methods section (e.g., under "Plasmids and plasmid construction" for the promoter, lines 557-560). We do not claim that targeting the major capsid protein is important for the method, it just has to be a potent crRNA to have sufficient selection (which is what the text says). We also do not know if it is important that the *acr* gene has to be expressed early and that the crRNA would need to target a late-expressed phage transcript. While one could speculate that there may or may not be such a bias, no consistent pattern regarding transposon positioning is apparent in our data and therefore also not mentioned. Notably, the Bas54 library has been selected with a crRNA targeting a DNA polymerase transcript (see line 647) – likely not a late gene – and still gave clear results with transposons in rather late genes like those coding for the tail fibers (Extended Data Fig. 7c). We look forward to future studies that could use transposon-encoded *acr* genes to study the role of expression timing for anti-CRISPR efficacy.

- p. 6 l. 102f. add reference to Fig. 1b again.

Response: Done – thank you for this helpful suggestion.

- Discussion: Please comment on the transferability to other species, the LseCas12a can be very likely transferred to Pseudomonas phages, as this system was used previously in Pseudomonas (PMID: 36316452).

Response: The transferability to other host bacteria is indeed an important point. Recent preprints by other labs already showed that similar technologies work in *P. aeruginosa* (Chan, Yee, et al., *bioRxiv* (2025)) or *Prodigiosinella* (Kyte et al., *bioRxiv* (2025)). We thus anticipate that HIDEN-SEQ will be applied in diverse host organisms in future studies. This is now explicitly highlighted in the discussion section (lines 355-364).

- Fig. 1b: I could not find a single gene with reduced fitness colouring, is this true, or is there a lack in colouring?

Response: Indeed, reviewer #2 is correct that no gene was classified as "reduced fitness" in case of T4. We considered changing the legend to remove this category, but then decided to leave it as is to avoid confusion for readers if maybe a different classification system (without this category) had been used specifically for this experiment.

- p. 28: please state the promoters used for the acr

Response: We have now added this information to the methods section on plasmid cloning ("[… cloned] under the control of constitutive promoters from the Anderson library (J23110 for AIcrVIA3; J23119 for AcrVIA1").

- p. 29, l. 532: Is here "clones" correct or should it be plaques.

Response: Indeed, should be "plaques" – we changed it. Thank you!

- p. 30: please state at which MOI the cells were infected with T4 to generate the library, also for the other phages.

Response: Cells were infected on plate by streaking out the phage with a loop, so no specific MOI was used. This is described in the methods section on "Construction of the T4 HIDEN-SEQ library": "To generate a transposon pool, phage T4 was grown to confluency on top agar plates of the transposon donor strain supplemented with IPTG for transposase induction." The same approach was used for the other phages (as indicated in their respective parts of the methods section).

- Fig. 1a, copy numbers of the plasmids should be indicated, and in the selection step the plasmid is missing. The blue transposase indication vs regular black genome in the bottom line will never be visible in b/w printouts, please mark it more prominently. The blue lines indicating transposon insertions show gaps between the genes, but actually, the intergenic regions are much more prone towards transposon insertions. The transposon insertion is not well depicted as there is no wt phage genome illustrated in the upper left part. The mechanism of inhibition by anti-CRISPR protein does not become evident, e.g. if the yellow transcript part (target) is transcribed at the same time as the blue (acr) the Cas13a will get activated, time-dependent events should be depicted clearly.

Response: We thank reviewer #2 for this detailed comment about Figure 1a. Based on this input, we have filled the gaps between insertion sites in the right part of the figure. However, we feel that the

requested details of plasmid copy number or a repeated depiction of the plasmids also in the right bacterial cell would unnecessarily overcrowd the figure. The procedure is described in extensive details in the methods section and also in the figure legend. We also prefer to not implement the other suggested changes regarding the coloring / stroke weight or the representation of CRISPR-Cas13a activity to avoid making the figure more busy and complicated.

- Extended Data Fig. 2B and similar other panels, essential and non-essential labels do not play a role here, and should be removed.

Response: We disagree with this assessment of reviewer #2 because the indicated labels for essential and non-essential genes enable readers to compare the plots before and after selection more clearly. While before selection the vast majority of genes carry many insertions and would thus *after selection* be classified as "non-essential", while after selection the category of "essential genes" with only few insertions becomes clearly visible.

Response to reviewers:
“Systematic mapping of bacteriophage gene essentiality with HIDEN-SEQ” (NMICROBIOL-25124570A)

Response to editorial / journal requests

In our revised manuscript, we have shortened the main manuscript down to <3’700 words and implemented different requests from the author checklist, e.g, regarding the number of extended data files, the setup of source data files, et cetera. The author checklist contains dedicated responses to each row of the table indicating which changes we have made.

Response to Reviewers #1/3

- Many thanks to the authors for addressing our comments through additional experiments and edits to the manuscript and figures. The additional validation of essentiality calls in T4 and Bas54 and the additional characterization of the transposon libraries substantially strengthen the manuscript and support the general utility of HIDEN-SEQ, and the edits have made the manuscript easier to follow. In total we congratulate the authors on developing HIDEN-SEQ, which we expect will be a valuable resource for the research community.
- Our remaining comment regards the discussion of polar effects. We appreciate the edits and the additional data in the revised manuscript but we still find the descriptions to be somewhat misleading. The authors appear to insist on their point that HIDEN-SEQ is not subject to polar effects, but this argument is 1) implausible given all the possible mechanisms that can give rise to polar effects (effects on transcript stability, transcript interference, translational coupling, and more); and 2) essentially impossible to prove especially without extensive documentation of a complete lack of polar effects across all phages. The experiment with a modified transposon that includes a transcriptional terminator is informative and provides strong evidence that the lack of a transcriptional terminator in the transposon used for HIDEN-SEQ mitigates some types of polar effects. It does not, however, allow for the inference that the remaining essentiality calls are not affected by polar effects. In addition, polar effects may particularly confound some of the intermediate fitness effects.
- We recognize that it is not necessary for the authors to entirely rule out the possibility of polar effects. Indeed, HIDEN-SEQ has substantial utility regardless, particularly as a tool to rapidly nominate genes for further study, and therefore we feel it would in no way diminish the impact of this study to accurately describe their findings and limitations with regards to polar effects. Statements such as “polar effects are not a major confounder” are much too vague. We recommend that the authors further modify their language, specifically the statements in lines 136-138 of the results section (“These results strongly suggest that polar effects do not significantly distort the HIDEN-SEQ data generated with our transposon that lacks a transcriptional terminator.”) and lines 323-332 of the discussion (especially “…our data indicate that polar effects are not a major confounder of HIDEN-SEQ results…”). For example, lines 136-138 could be a statement that the results suggest that the use of a transposon without a transcriptional terminator mitigates some types of polar effects and improves the accuracy of essential gene identification, and the discussion could state that the authors accurately recall essential genes of T4, aided in part by the lack of a transcriptional terminator in their transposon, but that polar effects can nonetheless influence fitness effects of insertions as well as results in other phages.

- (In case the authors do want to continue making the claim that HIDEN-SEQ is not confounded by polar effects, the burden would be on them to prove it. Specifically, they would have to demonstrate that each call that could in principle arise from a polar effect is accurate. For example, from an inspection of the Bas54 essentiality map, many essentiality/partial essentiality "calls" have patterns that would be consistent with polar effects in principle, including 26-27, 86, 100-103, 134, 181-183, and 196, among others. We did not cross-reference these genes against known databases of essential genes in related phages, but none of these genes were included in the orthogonal validation that the authors provided.)

Response: We thank reviewers #1 and #3 for their additional helpful comments regarding polar effects. Importantly, the need to significantly shorten the manuscript from ca. 4'500 words to below 3'700 words has resulted in a move of most technicalities regarding polar effects to a dedicated section in the supplementary discussion (in agreement with editorial feedback about this). Both in the main text and in the supplementary discussion, we have dampened our statements as suggested by this reviewer. As an example, the main text now avoids any direct statement regarding the extent of polar effects besides the observation that our results for T4 are largely in line with previous literature and that polar effects can be strong if they are deliberately provoked by introducing a transcriptional terminator to the transposon (see lines 102-106 of the clean main text).

In the supplementary discussion, we now discuss polar effects and other confounding factors in more detail and focus particularly on phage T4 where HIDEN-SEQ results can be compared to previous literature. In this section, we write that "the large agreement of our HIDEN-SEQ results for phage T4 with previous work suggests that polar effects do not greatly affect the results obtained with our technology" which we find is fully justified. Subsequently, we follow the suggestion of this reviewer and write: "However, it is clear that polar effects can nonetheless influence fitness effects of insertions and gene essentiality calls in other phages. It is therefore important to validate key results with orthogonal approaches such as clean knockouts, transcriptional knockdowns, or conditional translational inhibition through amber codons."

- On a minor note, the authors describe the insertion bias alongside their Bas54 transposon library. Might it be more appropriate to introduce this effect earlier, during the characterization of the T4 library?

Response: We agree with this reviewer that the description and discussion of the insertion bias had been positioned a bit awkwardly with the Bas54 transposon library. This observation is now (in a slightly more expanded way) included in the supplementary discussion.

Response to Reviewer #2

- I appreciate the authors' revisions. My previous concerns have been adequately addressed.

Response: Thank you for the positive feedback. We appreciate your careful review of our manuscript and are glad that you find our revisions have adequately addressed your previous concerns.
